# Supplementary material for: Exploring the combined impact of hepatitis B antibody status and systemic immune-inflammation index on mortality risk: A population-based study
Source: PLoS One. 2025 Jul 21;20(7):e0328400. doi: 10.1371/journal.pone.0328400 (PMC12279158; doi:10.1371/journal.pone.0328400)
Supplement: Attachment — Submitted filename: PONE-D-25-05613_R1 reviewed again on 21st April 2025.pdf [file pone.0328400.s003.pdf]

PLOS ONE

Exploring the Combined Impact of Hepatitis B Antibody Status and Systemic Immune-Inflammation Index on Mortality Risk: A Population-Based Study

--Manuscript Draft--

**Manuscript Number:**	PONE-D-25-05613R1
**Article Type:**	Research Article
**Full Title:**	Exploring the Combined Impact of Hepatitis B Antibody Status and Systemic Immune-Inflammation Index on Mortality Risk: A Population-Based Study
**Short Title:**	Impact of SII and HBV Antibody Status on Mortality Risk
**Corresponding Author:**	Jiong Lu<br>West China Hospital of Sichuan University<br>chengdu, CHINA
**Keywords:**	Systemic immune-inflammation index; Hepatitis B virus; Immunology; all-cause mortality
**Abstract:**	Background<br><br>Chronic hepatitis B virus (HBV) infection is a significant global health issue, leading to liver-related morbidity and mortality. The systemic immune-inflammation index (SII), a marker of systemic inflammation and immune response, may predict disease outcomes. However, its role in HBV infection and its relationship with HBV surface antibody (HBsAb) status is not well understood. This study investigates the association between SII, HBsAb status, and their combined effects on all-cause and cardiovascular disease (CVD) mortality.<br><br>Methods<br><br>We analyzed data from 43,539 participants in the National Health and Nutrition Examination Survey (NHANES), categorizing them into four groups based on SII and HBsAb status: high/low SII with HBsAb-negative/positive. Mortality outcomes were assessed using Cox proportional hazards models adjusted for age, sex, race/ethnicity, BMI, and comorbidities.<br><br>Results<br><br>In the analysis of 43,539 participants, the fully adjusted model revealed that SII was significantly associated with both all-cause mortality (HR = 1.138, p < 0.001) and cardiovascular disease mortality (HR = 1.402, p < 0.0001), indicating that higher SII independently increases the risk of both outcomes. While the crude model showed a protective effect of HBV surface antibody on all-cause mortality (HR = 0.491, p < 0.0001) and cardiovascular disease mortality (HR = 0.478, p < 0.0001), this effect diminished after full adjustment. Additionally, the combined effect of SII and HBV surface antibody on both mortality outcomes remained significant in the fully adjusted model (HR = 1.402, p < 0.0001).<br><br>Conclusion<br><br>Higher SII is independently associated with increased risks of all-cause and cardiovascular disease mortality. The protective effect of HBV surface antibody on mortality diminished after adjustment for confounders. The combined effect of SII and HBV surface antibody on mortality highlights the complex interaction between inflammation and immune response in chronic HBV infection. SII may serve as a useful predictor of long-term health risks in HBV-infected individuals.
**Order of Authors:**	Di Zeng
	Shaofeng Wang
	Jiong Lu

Powered by Editorial Manager® and ProduXion Manager® from Aries Systems Corporation

	Bei Li
**Response to Reviewers:**	Response letter to Reviewers<br>Dear Editors and Reviewers,<br>We would like to thank you for reading our paper and sharing very valuable comments, concerning the manuscript we submitted entitled “Exploring the Combined Impact of Hepatitis B Antibody Status and Systemic Immune-Inflammation Index on Mortality Risk: A Population-Based Study”.<br>We have carefully looked through the points where you have made a comment, and a revision was made based on the original manuscript which we hope to meet with approval. Here, we would like to address point-by-point concerning the details (the replies are highlighted in blue).<br><br>Reviewer #1<br><br>Comment 1: What were the other covariates considered for full adjustment outside those listed in the results?<br><br>Reply 1:We sincerely thank the reviewer for this critical question. Our multivariable models were adjusted in a stepwise manner as follows:<br>Crude Model: Adjusted only for HBV surface antibody status.<br>Model 1: Adjusted for HBV surface antibody status, age, and sex.<br>Model 2: Added socioeconomic and demographic covariates:<br>Race/ethnicity (eth1)<br>Family income (Family_income)<br>Education level (edu)<br>Marital status (marital)<br>Model 3 (Fully Adjusted): Further included metabolic, behavioral, and clinical covariates:<br>Anthropometric: Body mass index (BMI)<br>Lipid Profile: Total cholesterol, LDL, HDL, triglycerides (TG)<br>Renal Function: CKD-EPI estimated glomerular filtration rate (CKD_EPI_Scr_2009)<br>Liver Function: Albumin<br>Behavioral Factors: Smoking status (smoke), alcohol use (alcohol.user)<br>Comorbidities: Chronic kidney disease (CKD), hypertension, chronic obstructive pulmonary disease (COPD)<br><br>These covariates were selected based on their established roles as confounders in mortality risk and prior literature on HBV and systemic inflammation. The stepwise adjustment approach ensures transparency in evaluating how additional covariates influence the association between HBV antibody status, SII, and mortality outcomes.<br><br>Changes in the text:<br>Added to the Statistical Analysis subsection in Methods:<br>"Cox proportional hazards models were adjusted in three sequential tiers:<br>Model 1: Age and sex; Model 2: Model 1 + race/ethnicity, family income, education, and marital status; Model 3: Model 2 + BMI, lipid profile (total cholesterol, LDL, HDL, TG), albumin, renal function (CKD-EPI eGFR), smoking, alcohol use, and comorbidities (CKD, hypertension, COPD)."<br><br>Comment 2: Was the nullification of the effect of HBVAb more pronounced with certain "other" covariates, if so which ones and to what extent? Was the effect due to a full combination of all covariates or just a combination of some and if so what combination(s)?<br>Reply 2: Thank you for raising this important question. In our analysis, the attenuation of HBVAb’s protective effect on mortality outcomes was most notably observed after adjusting for age, comorbidities (hypertension, CKD, COPD), and metabolic factors (BMI, lipid profile). While socioeconomic covariates (e.g., income, education) had minimal impact on the association, the inclusion of clinical and metabolic variables in Model 3 substantially contributed to the nullification of the effect. This attenuation was not attributable to any single covariate but rather emerged from the combined adjustment of age, metabolic status, and comorbidities. For example, age and comorbidities likely reflect the cumulative burden of chronic disease and frailty, which may dominate mortality risk in older populations, thereby overshadowing the modest protective effects of HBVAb. Similarly, metabolic factors such as BMI and lipid levels

Powered by Editorial Manager® and ProduXion Manager® from Aries Systems Corporation

may confound the relationship by linking systemic inflammation to both HBV immunity and mortality.
Changes in the text: Based on comment 2, we have added the following paragraph to Discussion part: "The observed attenuation of HBVAb's association with mortality after full adjustment likely reflects the dominant influence of age-related comorbidities (e.g., hypertension, CKD, COPD) and metabolic dysfunction (e.g., elevated BMI, dyslipidemia) on mortality risk. These factors collectively contribute to systemic inflammatory states and physiological frailty, which may overshadow the independent protective effects of HBV immunity. For instance, aging is strongly associated with chronic low-grade inflammation ("inflammaging") and reduced immune resilience, while comorbidities such as CKD and hypertension exacerbate oxidative stress and endothelial dysfunction—pathways that independently drive mortality risk. Furthermore, metabolic disturbances like obesity and dyslipidemia are linked to persistent immune activation and pro-inflammatory cytokine production, which may mask the modest anti-inflammatory benefits conferred by HBV surface antibodies. This aligns with prior studies demonstrating that metabolic syndrome and chronic inflammation attenuate vaccine-induced immune responses in older adults. Our findings suggest that in populations with high baseline inflammatory burden or multimorbidity, the protective role of HBVAb may be diluted by competing risk factors. Clinically, this underscores the importance of contextualizing HBV immune status within a patient's broader metabolic and comorbidity profile when assessing mortality risk. Future studies should explore whether targeted interventions to reduce systemic inflammation or manage comorbidities could unmask or amplify the protective effects of HBV immunity in specific subpopulations.
."

Comment 3: What is the unit of time measurement on the x-axes of the graphs? months, years, age?
Reply 3: We sincerely thank the reviewer for highlighting this omission. The x-axis of all survival curves (Figures 1a–3c) represents follow-up time in weeks. We have revised the figure captions and corresponding descriptions in the Results section to explicitly clarify the unit of time measurement.
Changes in the text: Based on Comment 3 , We have added to the Results section :Survival analyses of all-cause mortality, cardiovascular disease (CVD)-related mortality, and non-CVD-related mortality in the high and low systemic immune-inflammation index (SII) groups are presented in Figure 1a, 1b, and 1c, respectively, with follow-up time measured in weeks.

Comment 4: The conclusion is misleading, should state that the correlation of HBVAb with mortality was not apparent when fully adjusted for by other factors.

Reply 4: We sincerely thank the reviewer for this crucial correction. We agree that the original conclusion could be misinterpreted and have revised it to explicitly clarify that the association between HBV surface antibody (HBVAb) status and mortality was no longer apparent after full adjustment for covariates.
Changes in the text: Based on Comment 4, we have revised Conclusion section:: This large-scale study demonstrates that elevated systemic immune-inflammation index (SII) independently predicts increased risks of all-cause and cardiovascular mortality, persisting after rigorous adjustment for demographic, metabolic, and clinical confounders. In contrast, the apparent protective association of hepatitis B surface antibody (HBVAb) positivity observed in unadjusted analyses was fully attenuated when accounting for age, comorbidities, and metabolic dysfunction, suggesting no direct causal link between HBVAb status and mortality. Critically, the joint effect analysis revealed that individuals with high SII and HBVAb-negative serostatus faced the highest mortality risk, underscoring the clinical value of integrating inflammatory and immune markers for risk stratification. Our findings advance the understanding of systemic inflammation as a central driver of mortality, transcending traditional risk factors. The robustness of SII across adjustment models supports its utility as a low-cost prognostic biomarker, particularly in resource-limited settings. Conversely, the nullification of HBVAb's association highlights the need to contextualize HBV immunity within populations' baseline inflammatory and comorbidity burdens.
Reviewer #2

Powered by Editorial Manager® and ProduXion Manager® from Aries Systems Corporation

Comment 1: What were the authors' cutoff values for high and low PNI, SII, and FIB-4 values? How do these cutoffs compare to those from previous studies?

Reply 1: We sincerely thank the reviewer for catching this oversight. The phrasing has been revised to “2010 to 2020” throughout the manuscript to ensure clarity and consistency.
Changes in the text: Revised in Methods section (Data Source subsection):
"Data from 2010 to 2020 were utilized to examine the relationship between hepatitis B virus (HBV) antibody status and the systemic immune-inflammation index (SII)."

Comment 2: Page 11: BMI should be written in full.
Reply 2: We thank the reviewer for pointing this out. We have expanded BMI to Body Mass Index (BMI) at its first mention in the manuscript to ensure clarity and adherence to academic writing standards.
Changes in the text: Based on Comment 2, we have made the following revisions:
Additionally, participants with complete survival data, as well as detailed information on covariates (such as age, sex, race/ethnicity, Body Mass Index (BMI), smoking history, diabetes, and hypertension), were included to ensure robust analysis.
Comment 3:Page 11: Why was data on alcohol intake omitted?
Reply 3:We have added the data on alcohol intake in the relevant section. Thank you for highlighting this.
Changes in the text: We have added the data on alcohol intake in the relevant section:
Statistical Analysis
Descriptive statistics were calculated for all included variables. Continuous variables were expressed as means ± standard deviations, while categorical variables were presented as frequencies and percentages. Differences in clinical characteristics between HBV antibody-positive and negative groups were evaluated using independent t-tests for continuous variables and chi-square tests for categorical variables. To assess the relationship between SII and HBV antibody status, logistic regression analysis was employed. Three models were constructed: the unadjusted model (without covariate adjustment), the minimally adjusted model (adjusting for age, sex, race/ethnicity, BMI, smoking, diabetes, and hypertension), and the fully adjusted model (adjusting for all covariates, including dietary factors and other health behaviors). Cox proportional hazards models were adjusted in three sequential tiers: Model 1: Age and sex; Model 2: Model 1 + race/ethnicity, family income, education, and marital status; Model 3: Model 2 + BMI, lipid profile (total cholesterol, LDL, HDL, TG), albumin, renal function (CKD-EPI eGFR), smoking, alcohol use, and comorbidities (CKD, hypertension, COPD).The association between SII and clinical outcomes, including liver function, was further evaluated using survival analysis techniques, such as Kaplan-Meier curves and Cox proportional hazards models.

Comment 4:Statistical analysis:
Was a bivariate analysis done? If yes, please include the results.
The dietary factors and health behaviors assessed should be clearly stated.
Reply 4:Thank you for highlighting these points. We conducted a bivariate analysis, and the results have been included. Our analysis models were structured as follows: crude model: HBV_surface_antibody; model 1: HBV_surface_antibody, age, sex; model 2: HBV_surface_antibody, age, sex, eth1, family_income, edu, marital; model 3: HBV_surface_antibody, age, sex, eth1, marital, family_income, edu, BMI, total_cholesterol, LDL, HDL, TG, albumin, CKD_EPI_Scr_2009, smoke, alcohol_user, CKD, hypertension, COPD. The relevant results are presented in the table; you may have missed them as they were not clearly displayed. We will ensure this is addressed in the revised manuscript.
Changes in the text: We have presented the relevant results in the table here. We will ensure that the table is visible in the revised version:

Comment 5:Page 12: The respective proportions of the four groups of patients should be included.

Reply 5:We thank the reviewer for highlighting this important point. The proportions of the four patient groups (high/low SII with HBsAb-negative/positive) are indeed presented in Table 1 of the manuscript. To ensure clarity, we have explicitly stated

Powered by Editorial Manager® and ProduXion Manager® from Aries Systems Corporation

these proportions in the Results section and confirmed that all tables are included in the revised submission.
Changes in the text: We have presented the relevant results in the table here. We will ensure that the table is visible in the revised version:

Comment 6:Page 12: The spelling of "included" should be corrected
Reply 6:We thank the reviewer for pointing out this mistake. We have corrected the spelling of "included" on page 12
Changes in the text:
This study included 43,539 patients, categorized into four groups based on systemic immune-inflammation index (SII) and hepatitis B surface antibody (HBsAb) status: high SII and HBsAb-negative (17,677), high SII and HBsAb-positive (4,090), low SII and HBsAb-negative (16,656), and low SII and HBsAb-positive (5,116). The mean age was 46.99 years, with older patients in the high SII groups (high SII/HBsAb-negative: 49.33 years) and younger patients in the low SII/HBsAb-positive group (39.80 years). Mean BMI was 28.80 kg/m², highest in the high SII/HBsAb-negative group (29.55 kg/m²) and lowest in the low SII/HBsAb-positive group (27.09 kg/m²). Lipid profiles showed higher triglycerides and total cholesterol in high SII groups, while HDL levels were higher in the low SII/HBsAb-positive group (1.42 mmol/L). Renal function, assessed by CKD-EPI eGFR, was lower in high SII groups (92.17 mL/min/1.73 m²) and higher in HBsAb-positive groups (100.73 mL/min/1.73 m²). Females predominated in the high SII/HBsAb-positive group (62.08%), while males were more common in the low SII/HBsAb-negative group (54.13%). Non-Hispanic Whites comprised the majority (69.81%), with higher diversity in the low SII/HBsAb-positive group. Education levels were higher in the high SII/HBsAb-positive group (69.63% above high school). Hypertension and CKD prevalence were higher in high SII groups, while COPD prevalence was lowest in the low SII/HBsAb-positive group (1.75%). In summary, high SII groups exhibited higher cardiometabolic risk profiles, whereas HBsAb-positive groups were younger with more favorable metabolic indicators. Details of included patients are shown in Table1.

Comment 7:No table was sighted. Could you kindly make available all the tables listed in the body of the manuscript so that the review process can be completed. Also make available the standard deviation value for age and BMI
Reply 7: We will ensure that all the tables listed in the manuscript are properly included and visible in the revised submission. Data related to standard deviation could be found in the table.
Changes in the text: We have included all the tables in the manuscript. Please refer to the end of the revised manuscript for details.

Reviewer #3

Comment 1: Some conclusions weaken after adjustment and the manuscript does not provide sufficient explanation for the biological mechanisms underlying these changes. The author should clarify the reproducibility of the study and consider providing additional explanations or sensitivity analysis to strengthen the interpretation of results

Reply 1: We have addressed this comment by expanding the discussion to explain the attenuation of HBVAb's protective effect after full adjustment. Specifically, the weakening of HBVAb's association with mortality likely reflects the dominant influence of age-related comorbidities (e.g., hypertension, CKD) and metabolic dysfunction (e.g., dyslipidemia, obesity) in driving mortality risk. These factors are closely linked to systemic inflammation ("inflammaging") and immune senescence, which may obscure the modest protective effects of HBV immunity. Chronic inflammation in populations with high comorbidity burdens can disrupt immune regulation, reducing the potential benefits of HBVAb. We have incorporated the following expanded discussion in the revised manuscript:

"The attenuation of HBVAb's association with mortality after full adjustment may be attributed to the interplay between chronic inflammation and immune exhaustion in populations with high comorbidity burdens. Aging and metabolic disorders promote a pro-inflammatory milieu (e.g., elevated IL-6, TNF-α) that overwhelms the anti-inflammatory effects of HBV surface antibodies. This aligns with evidence that

metabolic syndrome accelerates immunosenescence, reducing the efficacy of vaccine-induced immunity in older adults. Thus, HBVAb's protective role may be context-dependent, limited to populations with preserved metabolic health and lower inflammatory activity."

We have also refined the conclusion to align with these findings:

"This large-scale study demonstrates that elevated systemic immune-inflammation index (SII) independently predicts increased risks of all-cause and cardiovascular mortality, persisting after rigorous adjustment for demographic, metabolic, and clinical confounders. In contrast, the apparent protective association of hepatitis B surface antibody (HBVAb) positivity observed in unadjusted analyses was fully attenuated when accounting for age, comorbidities, and metabolic dysfunction, suggesting no direct causal link between HBVAb status and mortality. Critically, the joint effect analysis revealed that individuals with high SII and HBVAb-negative serostatus faced the highest mortality risk, underscoring the clinical value of integrating inflammatory and immune markers for risk stratification. Our findings advance the understanding of systemic inflammation as a central driver of mortality, transcending traditional risk factors. The robustness of SII across adjustment models supports its utility as a low-cost prognostic biomarker, particularly in resource-limited settings. Conversely, the nullification of HBVAb's association highlights the need to contextualize HBV immunity within populations' baseline inflammatory and comorbidity burdens."
Changes in the text: Based on comment1, we added this paragraph in discussion section: The observed attenuation of HBVAb's association with mortality after full adjustment likely reflects the dominant influence of age-related comorbidities (e.g., hypertension, CKD, COPD) and metabolic dysfunction (e.g., elevated BMI, dyslipidemia) on mortality risk. These factors collectively contribute to systemic inflammatory states and physiological frailty, which may overshadow the independent protective effects of HBV immunity. For instance, aging is strongly associated with chronic low-grade inflammation ("inflammaging") and reduced immune resilience, while comorbidities such as CKD and hypertension exacerbate oxidative stress and endothelial dysfunction—pathways that independently drive mortality risk. Furthermore, metabolic disturbances like obesity and dyslipidemia are linked to persistent immune activation and pro-inflammatory cytokine production, which may mask the modest anti-inflammatory benefits conferred by HBV surface antibodies. This aligns with prior studies demonstrating that metabolic syndrome and chronic inflammation attenuate vaccine-induced immune responses in older adults. Our findings suggest that in populations with high baseline inflammatory burden or multimorbidity, the protective role of HBVAb may be diluted by competing risk factors. Clinically, this underscores the importance of contextualizing HBV immune status within a patient's broader metabolic and comorbidity profile when assessing mortality risk. Future studies should explore whether targeted interventions to reduce systemic inflammation or manage comorbidities could unmask or amplify the protective effects of HBV immunity in specific subpopulations.
We revised the Conlusion section as follows: This large-scale study demonstrates that elevated systemic immune-inflammation index (SII) independently predicts increased risks of all-cause and cardiovascular mortality, persisting after rigorous adjustment for demographic, metabolic, and clinical confounders. In contrast, the apparent protective association of hepatitis B surface antibody (HBVAb) positivity observed in unadjusted analyses was fully attenuated when accounting for age, comorbidities, and metabolic dysfunction, suggesting no direct causal link between HBVAb status and mortality. Critically, the joint effect analysis revealed that individuals with high SII and HBVAb-negative serostatus faced the highest mortality risk, underscoring the clinical value of integrating inflammatory and immune markers for risk stratification. Our findings advance the understanding of systemic inflammation as a central driver of mortality, transcending traditional risk factors. The robustness of SII across adjustment models supports its utility as a low-cost prognostic biomarker, particularly in resource-limited settings. Conversely, the nullification of HBVAb's association highlights the need to contextualize HBV immunity within populations' baseline inflammatory and comorbidity burdens.

Comment 2: Need to explain more clearly the criteria for classifying high/low SII, and how to stratify based on clinical risk groups.

Reply 2: The classification of SII into high and low groups was performed based on the status of HBsAb. First, participants were divided into two groups based on their HBsAb status: positive and negative. In the HBsAb-positive group, the Systemic Immune-Inflammation Index (SII) was categorized into high and low groups using the R package surv_cutpoint. Similarly, in the HBsAb-negative group, SII was also categorized into high and low groups using the same R package. This approach resulted in four distinct groups based on both HBsAb status and SII levels.

Changes in the text: Based on Comment 2, we have added a clarification on the grouping criteria in the relevant section of the Results: The classification of SII into high and low groups was performed based on the status of HBsAb. First, participants were divided into two groups based on their HBsAb status: positive and negative. In the HBsAb-positive group, the Systemic Immune-Inflammation Index (SII) was categorized into high and low groups using the R package surv_cutpoint. Similarly, in the HBsAb-negative group, SII was also categorized into high and low groups using the same R package.

Comment 3: Authors may consider providing more details about the specific NHANES datasets and variables used to enhance the reproducibility of the study.

Reply 3: We have addressed this comment by providing more details about the specific NHANES datasets and variables used in our study. These details are now clearly presented in Table 1, which includes relevant information for enhancing the reproducibility of the study

Comment 4: Write concisely, avoid redundancy. Some sentences are lengthy and repeat ideas from the Results section. The author should focus on the key findings of the research instead of explaining each numerical detail.
Reply 4: We appreciate the reviewer's valuable feedback. We acknowledge the concern regarding redundancy and overly lengthy sentences. In response, we have revised the manuscript to be more concise, focusing on the key findings of the research. We have removed repetitive explanations and ensured that the Results section remains succinct while still conveying the necessary information. We hope that these revisions improve the clarity and readability of the manuscript. Thank you for your helpful suggestion.
Changes in the text: Based on Comment 4, we revised the results paragraphs:
HBV Surface Antibody and All-Cause Mortality:
HBV antibody positivity showed a lower risk of all-cause mortality in the crude model (HR = 0.491, $p < 0.0001$). After adjusting for age and sex (Model 1), the HR increased and became non-significant (HR = 0.909, $p = 0.072$). Further adjustments in Models 2 and 3 also showed no significant association (HR = 0.878, $p = 0.018$ and HR = 0.906, $p = 0.086$, respectively), indicating that the protective effect of HBV antibody positivity diminishes after controlling for other factors. Details are provided in Table 3A.

HBV Surface Antibody and Cardiovascular Mortality:
HBV antibody positivity showed a protective effect against cardiovascular mortality in the crude model (HR = 0.478, $p < 0.0001$). However, after adjustments for age and sex (Model 1), the HR became non-significant (HR = 0.981, $p = 0.829$). Models 2 and 3 also showed no significant association (HR = 0.920, $p = 0.347$ and HR = 0.946, $p = 0.551$, respectively). Details are provided in Table 3B. All-cause mortality, CVD death, and non-CVD death for HBV antibody-negative and -positive groups are shown in Figures 2a, 2b, and 2c.

Combined Effect of SII and HBV Surface Antibody on All-Cause Mortality:
The combination of high SII and HBV antibody-negative status showed a strong association with increased all-cause mortality. In the crude model, the HR was 1.575 ($p < 0.0001$), and after adjustments for age and sex, it remained significant (HR = 1.590, $p < 0.0001$). Models 2 and 3 confirmed the continued association between high SII and higher mortality risk (HR = 1.655, $p < 0.0001$ in Model 2 and HR = 1.402, $p < 0.0001$ in

Model 3). Details are provided in Table 4A.

Combined Effect of SII and HBV Surface Antibody on Cardiovascular Mortality:
The combination of high SII and HBV antibody-negative status showed the highest cardiovascular mortality risk. In the crude model, the HR was 1.575 ($p < 0.0001$), and after age and sex adjustments, it remained significant (HR = 1.590, $p < 0.0001$). Models 2 and 3 continued to show a strong association between high SII and poor cardiovascular outcomes, with higher HR values in the highest SII group. Details are provided in Table 4B. The all-cause mortality, CVD death, and non-CVD death for the combined SII and HBV antibody status groups are shown in Figures 3a, 3b, and 3c.

Comment 5: Clearly state the biological mechanisms and clinical significance behind the observed reduction in the protective effect of HbsAb after adjusting for confounders.

Reply 5: We thank the reviewer for emphasizing the importance of clarifying the biological and clinical underpinnings of our findings. As suggested, we have expanded the Discussion section to explicitly address the mechanisms and significance of the attenuated HBsAb-mortality association
Changes in the text: Based on comment 5, we have added the following paragraph to Discussion part: "The observed attenuation of HBVAb's association with mortality after full adjustment likely reflects the dominant influence of age-related comorbidities (e.g., hypertension, CKD, COPD) and metabolic dysfunction (e.g., elevated BMI, dyslipidemia) on mortality risk. These factors collectively contribute to systemic inflammatory states and physiological frailty, which may overshadow the independent protective effects of HBV immunity. For instance, aging is strongly associated with chronic low-grade inflammation ("inflammaging") and reduced immune resilience, while comorbidities such as CKD and hypertension exacerbate oxidative stress and endothelial dysfunction—pathways that independently drive mortality risk. Furthermore, metabolic disturbances like obesity and dyslipidemia are linked to persistent immune activation and pro-inflammatory cytokine production, which may mask the modest anti-inflammatory benefits conferred by HBV surface antibodies. This aligns with prior studies demonstrating that metabolic syndrome and chronic inflammation attenuate vaccine-induced immune responses in older adults. Our findings suggest that in populations with high baseline inflammatory burden or multimorbidity, the protective role of HBVAb may be diluted by competing risk factors. Clinically, this underscores the importance of contextualizing HBV immune status within a patient's broader metabolic and comorbidity profile when assessing mortality risk. Future studies should explore whether targeted interventions to reduce systemic inflammation or manage comorbidities could unmask or amplify the protective effects of HBV immunity in specific subpopulations.

Comment 6: Compare findings with previous studies to clarify how the results on SII and HbsAb in this manuscript complement or differ from existing literature?

Reply 6: We appreciate the reviewer's insightful comment. Our study addresses a novel research question, as no previous literature has specifically examined the combined impact of SII and HBsAb status on mortality risk. However, our findings on the prognostic value of SII align with and extend emerging evidence from HBV-related liver disease research. For instance, a study by Xue et al. demonstrated that elevated SII (>1024) independently predicts cirrhosis progression in CHB patients with comorbid NAFLD (HR=1.885, p=0.010), highlighting the role of systemic inflammation in hepatic fibrogenesis. Similarly, a 2024 study reported that SII effectively distinguishes immune-active from immune-tolerant phases in untreated CHB patients, outperforming qHBsAg in HBeAg-positive subgroups. While our study focuses on mortality rather than disease phase classification, these findings reinforce SII's sensitivity to immune-inflammatory dynamics in HBV infection. In advanced HBV-HCC cohorts, Yao et al. (2024) identified SII ≤539.47 as an independent protective factor for progression-free survival during immunotherapy, with reductions in SII correlating with improved survival outcomes. Although our study does not involve cancer patients, this supports the broader clinical relevance of SII in HBV-associated diseases. Notably, our work diverges by evaluating

Powered by Editorial Manager® and ProduXion Manager® from Aries Systems Corporation

SII in conjunction with HBsAb status—a novel approach that identified high SII/HBsAb-negative individuals as the highest-risk subgroup, a finding not reported in prior literature. These insights position SII as a transdiagnostic biomarker, bridging hepatic and systemic inflammatory risk assessment in HBV management.
Changes in the text: Based on comment 6, we have added the following paragraph to Discussion part:
While our study pioneers the exploration of SII and HBsAb as joint predictors of mortality in a general population, our findings align with emerging evidence on SII's prognostic role in HBV-related pathologies. For instance, a recent study by Guo et al. (2023) in Frontiers in Oncology demonstrated that elevated SII predicts early recurrence in HBV-related hepatocellular carcinoma (HCC) patients after surgery, underscoring its utility in oncology settings. Similarly, our results confirm SII's broader relevance as a marker of systemic inflammation and mortality risk, extending its prognostic value beyond cancer to all-cause and cardiovascular mortality in the general population. Our findings on the prognostic value of SII align with and extend emerging evidence from HBV-related liver disease research. A recent study by Xue et al. demonstrated that elevated SII (cutoff >1024) independently predicts cirrhosis progression in CHB patients with comorbid NAFLD (HR=1.885, p=0.010), emphasizing its role in hepatic fibrogenesis through systemic inflammation(26). This complements our observation that SII remains a robust mortality predictor even after adjusting for metabolic comorbidities, suggesting its broad utility across both liver-specific and systemic outcomes. Furthermore, a 2024 study reported that SII effectively distinguishes immune-active (EPCH/ENCH) from immune-tolerant (EPCI/ENCI) phases in untreated CHB patients, with higher accuracy than qHBsAg in HBeAg-positive subgroups(27). While our study focuses on mortality rather than disease phase classification, these results collectively highlight SII's sensitivity to immune-inflammatory dynamics in HBV infection. Notably, our work diverges by evaluating SII in conjunction with HBsAb status—a novel approach that revealed high SII/HBsAb-negative individuals as the highest-risk subgroup, a finding unreported in prior literature. Importantly, in advanced HBV-HCC cohorts, Yao et al. (2024) identified SII ≤539.47 as an independent protective factor for progression-free survival (HR=0.54, p=0.03) during immunotherapy, with longitudinal SII reductions correlating with improved survival(28). Though our population lacks cancer diagnoses, this reinforces the clinical relevance of SII monitoring across HBV disease spectra. Together, these studies position SII as a transdiagnostic biomarker, bridging hepatic and systemic inflammatory risk assessment in HBV management.

Reviewer #4

Comment 1: I suggest that the authors review the existing literature on the SII in hepatitis B-related outcomes. Several published papers have assessed the prediction of SII in patients with hepatitis B (e.g., DOI: 10.1007/s12094-024-03596-0, DOI: 10.1097/MEG.0000000000002737, DOI: https://doi.org/10.3855/jidc.19636), and emphasizing the novel contributions of your study.

Reply 1: We thank the reviewer for this valuable suggestion. We have carefully reviewed the recommended literature and incorporated relevant insights into the revised Discussion section.
Changes in the text: Based on Comment 1, we have added this paragraph to Discussion section:
Our findings on the prognostic value of SII align with and extend emerging evidence from HBV-related liver disease research. A recent study by Xue et al. demonstrated that elevated SII (cutoff >1024) independently predicts cirrhosis progression in CHB patients with comorbid NAFLD (HR=1.885, p=0.010), emphasizing its role in hepatic fibrogenesis through systemic inflammation. This complements our observation that SII remains a robust mortality predictor even after adjusting for metabolic comorbidities, suggesting its broad utility across both liver-specific and systemic outcomes. Furthermore, a 2024 study reported that SII effectively distinguishes immune-active

Powered by Editorial Manager® and ProduXion Manager® from Aries Systems Corporation

(EPCH/ENCH) from immune-tolerant (EPCI/ENCI) phases in untreated CHB patients, with higher accuracy than qHBsAg in HBeAg-positive subgroups. While our study focuses on mortality rather than disease phase classification, these results collectively highlight SII's sensitivity to immune-inflammatory dynamics in HBV infection. Notably, our work diverges by evaluating SII in conjunction with HBsAb status—a novel approach that revealed high SII/HBsAb-negative individuals as the highest-risk subgroup, a finding unreported in prior literature. Importantly, in advanced HBV-HCC cohorts, Yao et al. (2024) identified SII ≤539.47 as an independent protective factor for progression-free survival (HR=0.54, p=0.03) during immunotherapy, with longitudinal SII reductions correlating with improved survival. Though our population lacks cancer diagnoses, this reinforces the clinical relevance of SII monitoring across HBV disease spectra. Together, these studies position SII as a transdiagnostic biomarker, bridging hepatic and systemic inflammatory risk assessment in HBV management.

Comment 2: To better align with the authors' statement, I suggest replacing reference number 8 with another source, such as this one (DOI: 10.4103/tjem.tjem_198_23)

Reply 2: We appreciate the reviewer's suggestion. We have replaced reference number 8 with the recommended source to better align with the statement.
Changes in the text: Based on Comment 2, we have replaced reference number 8 with the recommended source (DOI: 10.4103/tjem.tjem_198_23).

Comment 3: I suggest that the authors clarify how they categorized the SII quartile from Q1 to Q4 in the method section to improve readers' comprehension.

Reply 3: We thank the reviewer for highlighting the need for methodological clarity. In our study, SII was categorized into quartiles (Q1–Q4) based on its distribution within the NHANES population. Quartile thresholds were defined as Q1 (<25th percentile), Q2 (25th–50th percentile), Q3 (50th–75th percentile), and Q4 (≥75th percentile), and participants were assigned to the corresponding group accordingly.
Changes in the text: Based on Comment 3, we have revised the Methods section: SII was categorized into quartiles (Q1–Q4) based on its distribution in the NHANES population: Q1 (<25th percentile), Q2 (25th–<50th percentile), Q3 (50th–<75th percentile), and Q4 (≥75th percentile)

Comment 4: I think it would be more comprehensive to provide proposed explanations based on existing literature or the authors' insights into why the protective effect of HBV surface antibody positivity decreased after adjusting for other factors in their study.
Reply 4: Thank you for the valuable suggestion. We have expanded the discussion to provide a more detailed explanation for the attenuation of HBV surface antibody's protective effect after full adjustment. The decrease in protective effect likely reflects the dominant influence of age-related comorbidities (e.g., hypertension, CKD) and metabolic dysfunction (e.g., dyslipidemia, obesity) on mortality risk. Chronic inflammation and immune senescence associated with these conditions may mask the modest protective effect of HBV surface antibodies. For instance, elevated pro-inflammatory cytokines (e.g., IL-6, TNF-α) in metabolically compromised populations may impair immune regulation, reducing the protective impact of HBV immunity. This aligns with evidence that metabolic syndrome accelerates immunosenescence, diminishing the efficacy of vaccine-induced immunity in older adults. We have incorporated this discussion in the revised manuscript to enhance clarity and depth.
Changes in the text: Based on Comment 4, we have added the following paragraph to Discussion part: "The observed attenuation of HBVAb's association with mortality after full adjustment likely reflects the dominant influence of age-related comorbidities (e.g., hypertension, CKD, COPD) and metabolic dysfunction (e.g., elevated BMI, dyslipidemia) on mortality risk. These factors collectively contribute to systemic inflammatory states and physiological frailty, which may overshadow the independent protective effects of HBV immunity. For instance, aging is strongly associated with chronic low-grade inflammation ("inflammaging") and reduced immune resilience, while comorbidities such as CKD and hypertension exacerbate oxidative stress and endothelial dysfunction—pathways that independently drive mortality risk. Furthermore, metabolic disturbances like obesity and dyslipidemia are linked to persistent immune

	activation and pro-inflammatory cytokine production, which may mask the modest anti-inflammatory benefits conferred by HBV surface antibodies. This aligns with prior studies demonstrating that metabolic syndrome and chronic inflammation attenuate vaccine-induced immune responses in older adults. Our findings suggest that in populations with high baseline inflammatory burden or multimorbidity, the protective role of HBVAb may be diluted by competing risk factors. Clinically, this underscores the importance of contextualizing HBV immune status within a patient's broader metabolic and comorbidity profile when assessing mortality risk. Future studies should explore whether targeted interventions to reduce systemic inflammation or manage comorbidities could unmask or amplify the protective effects of HBV immunity in specific subpopulations.<br><br><br>Comment 5: References numbered 18, 23, and 24 are not relevant to the authors' discussion in the text. The authors should review these references to ensure they are cited appropriately in relation to their discussion.<br>Reply 5:We appreciate the reviewer's attention to detail. We have carefully reviewed references 18, 23, and 24 and agree that they were not appropriately aligned with the discussion. These references have been either revised or removed to ensure consistency and relevance in the revised manuscript.<br>Comment 6: I do not see any result tables (Table 1 to Table 4) in the submitted manuscript; therefore, I could not review and comment thoroughly on these results.<br>Reply 6: We will ensure that all the tables listed in the manuscript are properly included and visible in the revised submission. Data related to standard deviation could be found in the table.<br>Changes in the text: We have included all the tables in the manuscript. Please refer to the end of the revised manuscript for details.<br><br>Reviewer #5<br><br>Comment 1: The authors explored the combined impact of Hepatitis B antibody status and Systemic Immune-Inflammation Index on Mortality Risk. The author categorised the participants into groups based on the presence or absence of HBs antibodies and analysed the resulting data. As the author states, the HBs antibody-positive group includes both those who have previously been infected with hepatitis B and those who have received vaccination. The inability to distinguish between these two groups is a limitation of the study. This is a limitation that should be stated explicitly.<br><br>Reply 1: We appreciate the reviewer's insightful comment. We agree that the inability to distinguish between individuals with prior HBV infection and those with vaccine-induced immunity is a notable limitation. This distinction could have important implications for interpreting the prognostic value of HBsAb positivity. We have explicitly acknowledged this limitation in the revised manuscript and discussed its potential impact on the study's findings.<br>Changes in the text: Based on Comment 1, we have explicitly stated in the limitations section that the inability to distinguish between past HBV infection and vaccine-induced immunity is a limitation of the study and discussed its potential impact on the findings: The inability to distinguish between past HBV infection and vaccine-induced immunity in the HBs antibody-positive group is a limitation that may influence the interpretation of the results.<br>We would like to sincerely thank the editor and reviewers for their thoughtful and constructive feedback. Your insights have been invaluable in improving the clarity and quality of the manuscript. We appreciate the time and effort you have dedicated to reviewing our work, and we believe the revisions made have strengthened the manuscript. We are grateful for your support and look forward to your continued guidance throughout the publication process.
**Additional Information:**	
**Question**	**Response**
**Financial Disclosure**<br><br><br>Enter a financial disclosure statement that describes the sources of funding for the	The author(s) received no specific funding for this work.

work included in this submission. Review the submission guidelines for detailed requirements. View published research articles from *PLOS ONE* for specific examples.<br><br>This statement is required for submission and **will appear in the published article** if the submission is accepted. Please make sure it is accurate.<br><br>**Funded studies**<br>Enter a statement with the following details:<br>• Initials of the authors who received each award<br>• Grant numbers awarded to each author<br>• The full name of each funder<br>• URL of each funder website<br>• Did the sponsors or funders play any role in the study design, data collection and analysis, decision to publish, or preparation of the manuscript?<br><br>Did you receive funding for this work?	
**Competing Interests**<br><br>Use the instructions below to enter a competing interest statement for this submission. On behalf of all authors, disclose any competing interests that could be perceived to bias this work—acknowledging all financial support and any other relevant financial or non-financial competing interests.<br><br>This statement is required for submission and **will appear in the published article** if the submission is accepted. Please make sure it is accurate and that any funding sources listed in your Funding Information later in the submission form are also declared in your Financial Disclosure statement.<br><br>View published research articles from *PLOS ONE* for specific examples.	The authors have declared that no competing interests exist.

<table>
<tr><td>

NO authors have competing interests

Enter: *The authors have declared that no competing interests exist.*

Authors with competing interests

Enter competing interest details beginning with this statement:

I have read the journal's policy and the authors of this manuscript have the following competing interests: [insert competing interests here]

* typeset

</td><td></td></tr>
<tr><td>

Ethics Statement

Enter an ethics statement for this submission. This statement is required if the study involved:

- Human participants
- Human specimens or tissue
- Vertebrate animals or cephalopods
- Vertebrate embryos or tissues
- Field research

Write "N/A" if the submission does not require an ethics statement.

General guidance is provided below. Consult the submission guidelines for detailed instructions. **Make sure that all information entered here is included in the Methods section of the manuscript.**

</td><td>

This study utilized data from the National Health and Nutrition Examination Survey (NHANES), a publicly available dataset provided by the Centers for Disease Control and Prevention (CDC). All data were anonymized prior to analysis, and NHANES participants provided informed consent at the time of data collection. The NHANES protocol is reviewed and approved by the National Center for Health Statistics (NCHS) Research Ethics Review Board, and all procedures are conducted in accordance with the Declaration of Helsinki.

</td></tr>
</table>

**Format for specific study types**<br><br>**Human Subject Research (involving human participants and/or tissue)**<br>• Give the name of the institutional review board or ethics committee that approved the study<br>• Include the approval number and/or a statement indicating approval of this research<br>• Indicate the form of consent obtained (written/oral) or the reason that consent was not obtained (e.g. the data were analyzed anonymously)<br><br>**Animal Research (involving vertebrate animals, embryos or tissues)**<br>• Provide the name of the Institutional Animal Care and Use Committee (IACUC) or other relevant ethics board that reviewed the study protocol, and indicate whether they approved this research or granted a formal waiver of ethical approval<br>• Include an approval number if one was obtained<br>• If the study involved *non-human primates*, add *additional details* about animal welfare and steps taken to ameliorate suffering<br>• If anesthesia, euthanasia, or any kind of animal sacrifice is part of the study, include briefly which substances and/or methods were applied<br><br>**Field Research**<br><br>Include the following details if this study involves the collection of plant, animal, or other materials from a natural setting:<br>• Field permit number<br>• Name of the institution or relevant body that granted permission	
**Data Availability**<br><br>Authors are required to make all data underlying the findings described fully available, without restriction, and from the time of publication. PLOS allows rare exceptions to address legal and ethical concerns. See the PLOS Data Policy and FAQ for detailed information.	Yes - all data are fully available without restriction

A Data Availability Statement describing where the data can be found is required at submission. Your answers to this question constitute the Data Availability Statement and **will be published in the article**, if accepted.<br><br>**Important:** Stating 'data available on request from the author' is not sufficient. If your data are only available upon request, select 'No' for the first question and explain your exceptional situation in the text box.<br><br>Do the authors confirm that all data underlying the findings described in their manuscript are fully available without restriction?	
**Describe where the data may be found in full sentences. If you are copying our sample text, replace any instances of XXX with the appropriate details.**<br><br>• If the data are **held or will be held in a public repository**, include URLs, accession numbers or DOIs. If this information will only be available after acceptance, indicate this by ticking the box below. For example: *All XXX files are available from the XXX database (accession number(s) XXX, XXX.*).<br>• If the data are all contained **within the manuscript and/or Supporting Information files**, enter the following: *All relevant data are within the manuscript and its Supporting Information files.*<br>• If neither of these applies but you are able to provide **details of access elsewhere**, with or without limitations, please do so. For example:<br><br>*Data cannot be shared publicly because of [XXX]. Data are available from the XXX Institutional Data Access / Ethics Committee (contact via XXX) for researchers who meet the criteria for access to confidential data.*<br><br>*The data underlying the results presented in the study are available from (include the name of the third party*	This study utilized data from the National Health and Nutrition Examination Survey (NHANES), a publicly available dataset provided by the Centers for Disease Control and Prevention (CDC). All data were anonymized prior to analysis, and NHANES participants provided informed consent at the time of data collection. The NHANES protocol is reviewed and approved by the National Center for Health Statistics (NCHS) Research Ethics Review Board, and all procedures are conducted in accordance with the Declaration of Helsinki. (https://www.cdc.gov/nchs/nhanes/index.htm).

and contact information or URL).<br>• This text is appropriate if the data are owned by a third party and authors do not have permission to share the data.<br><br>* typeset	
Additional data availability information:	

Powered by Editorial Manager® and ProduXion Manager® from Aries Systems Corporation

manuskript Click here to access/download;Manuscript;manuskript.docx

Exploring the Combined Impact of Hepatitis B Antibody Status and Systemic Immune-Inflammation Index on Mortality Risk: A Population-Based Study

TITLE PAGE

Article type
Original article

Full title of manuscript

Exploring the Combined Impact of Hepatitis B Antibody Status and Systemic Immune-Inflammation Index on Mortality Risk: A Population-Based Study

Running head

Impact of SII and HBV Antibody Status on Mortality Risk

List of authors
Di Zeng (B.S.)[1,2] , Shaofeng Wang(B.S.)[1,2], Nansheng Cheng (M.D.)[1,2]*, Bei Li (M.D.)[1,2]*

[1]Division of Biliary Surgery, Department of General Surgery, West China Hospital, Sichuan University, Chengdu 610041, Sichuan, China
[2]Research Center for Biliary Diseases, West China Hospital, Sichuan University, Chengdu 610041, Sichuan, China

Di Zeng (B.S.)1,2 , Shaofeng Wang(B.S.)1,2 contributed equally to this work as co-first authors.

Nansheng Cheng (M.D.)1,2*, Bei Li (M.D.)1,2* are co-corresponding authors of this study.

Conflict-of-interest statement:
All authors have no conflicts of interest regarding the current paper.

Author contributions:

Di Zeng: Conceptualization (lead); Data curation (lead); Formal analysis (lead); Investigation (lead); Methodology (lead); Writing – original draft (lead); Writing – review & editing (lead).**Shaofeng Wang** : Conceptualization (equal); Formal analysis (equal); Investigation (equal); Writing – review & editing (equal). **Nansheng Cheng**: Investigation (equal); Writing – review &

editing (equal); Methodology (lead); Supervision (equal). **Bei Li:** Supervision (lead); Writing – review & editing (lead).

Funding information
This study did not receive any funding.

Correspondence to:
1. **Nansheng Cheng**: (M.D.), Division of Biliary Tract Surgery, Department of General Surgery, West China Hospital, Sichuan University, Chengdu 610041, Sichuan, China.
2. **Bei Li**: (M.D.), Division of Biliary Tract Surgery, Department of General Surgery, West China Hospital, Sichuan University, Chengdu 610041, Sichuan, China.
Email: nanshengcheng@yeah.net; 1017346985@qq.com

Exploring the Combined Impact of Hepatitis B Antibody Status and Systemic Immune-Inflammation Index on Mortality Risk: A Population-Based Study

Abstract:

Background: Chronic hepatitis B virus (HBV) infection is a significant global health issue, leading to liver-related morbidity and mortality. The systemic immune-inflammation index (SII), a marker of systemic inflammation and immune response, may predict disease outcomes. However, its role in HBV infection and its relationship with HBV surface antibody (HBsAb) status is not well understood. This study investigates the association between SII, HBsAb status, and their combined effects on all-cause and cardiovascular disease (CVD) mortality.

Methods: We analyzed data from 43,539 participants in the National Health and Nutrition Examination Survey (NHANES), categorizing them into four groups based on SII and HBsAb status: high/low SII with HBsAb-negative/positive. Mortality outcomes were assessed using Cox proportional hazards models adjusted for age, sex, race/ethnicity, BMI, and comorbidities.

Results: In the analysis of 43,539 participants, the fully adjusted model revealed that SII was significantly associated with both all-cause mortality (HR = 1.138, $p < 0.001$) and cardiovascular disease mortality (HR = 1.402, $p < 0.0001$), indicating that higher SII independently increases the risk of both outcomes. While the crude model showed a protective effect of HBV surface antibody on all-cause mortality (HR = 0.491, $p < 0.0001$) and cardiovascular disease mortality (HR = 0.478, $p < 0.0001$), this effect diminished after full adjustment. Additionally, the combined effect of SII and HBV

surface antibody on both mortality outcomes remained significant in the fully adjusted model (HR = 1.402, $p < 0.0001$).

Conclusion: Higher SII is independently associated with increased risks of all-cause and cardiovascular disease mortality. The protective effect of HBV surface antibody on mortality diminished after adjustment for confounders. The combined effect of SII and HBV surface antibody on mortality highlights the complex interaction between inflammation and immune response in chronic HBV infection. SII may serve as a useful predictor of long-term health risks in HBV-infected individuals.

Key words: Systemic Immune-Inflammation Index; Hepatitis B Virus; Immunology; All-Cause Mortality

1. Introduction

Hepatitis B virus (HBV) infection remains a major global health concern(1), with an estimated 250 million people living with chronic HBV worldwide(2). The World Health Organization (WHO) has highlighted HBV as a leading cause of liver-related morbidity and mortality, contributing significantly to the development of liver cirrhosis, liver failure, and hepatocellular carcinoma (HCC)(3). Despite the availability of effective vaccines, which have reduced new HBV infections globally, chronic HBV remains endemic in regions such as Asia and sub-Saharan Africa(4). Chronic HBV infection can result in a range of adverse clinical outcomes, which are influenced by multiple factors such as the immune status of the host, viral load, and comorbidities including non-alcoholic fatty liver disease (NAFLD) and metabolic syndrome(5). These factors not only complicate the management of HBV but also contribute to an increased risk of liver fibrosis, cirrhosis, and the eventual development of HCC(6). Understanding the mechanisms that drive HBV progression and the impact of various biomarkers is essential for predicting disease outcomes and tailoring effective treatment strategies.

One such promising biomarker is the systemic immune-inflammation index (SII), which has garnered attention for its ability to predict prognosis in a variety of cancers, including HCC(7). The SII is calculated as the product of platelet count and neutrophil count, divided by the lymphocyte count, reflecting a balance between inflammation and immune response(8). A higher SII has been associated with a pro-tumor inflammatory state, characterized by elevated neutrophils and platelets, alongside a decrease in lymphocytes(9). This inflammatory imbalance is believed to compromise immune surveillance, thus facilitating tumor progression(10). Recent studies have linked high SII with poorer overall survival (OS) and disease-free survival (DFS) across several cancer types, including colorectal, gastric, and esophageal cancers(11-13). However, its role in HBV infection, particularly in relation to HBV antibody status, remains underexplored.

HBV antibody status plays a critical role in determining the immune response to the virus(14). Individuals who are positive for hepatitis B surface antibody (anti-HBs) may be more likely to maintain a more controlled immune response to HBV, which could potentially influence the systemic inflammatory status in these individuals(15). This might manifest as a comparatively lower systemic immune-inflammation index (SII), though this relationship is complex and could be influenced by various factors. On the other hand, those who are negative for HBV antibodies may experience a different immune dynamic, which could be associated with a more pronounced inflammatory state, possibly reflected in a higher SII(16).

The potential differences in immune profiles and inflammatory responses between HBV antibody-positive and -negative individuals require further investigation, as they may provide valuable insights into the immune dynamics of HBV infection and its progression. Our research suggests that variations in SII could have important implications for disease outcomes. A higher SII in HBV-positive individuals might indicate a more severe inflammatory state, contributing to liver fibrosis and hepatocellular carcinoma, while a lower SII in antibody-positive individuals may reflect better immune control and liver function. By exploring these associations, our study aims to deepen the understanding of HBV pathophysiology and inform the development of personalized therapeutic approaches, including optimized antiviral and immunomodulatory treatments for HBV patients.

2. Materials and Methods

1. Data Source

Data for this study were obtained from the National Health and Nutrition Examination Survey (NHANES), a nationally representative survey conducted by the Centers for Disease Control and Prevention (CDC) in the United States. The NHANES collects comprehensive data on the health, nutrition, and socioeconomic status of the civilian, non-institutionalized population. In this study, data from 2010 years to 2020 years were utilized to examine the relationship between hepatitis B virus (HBV) antibody status and the systemic immune-inflammation index (SII). The NHANES datasets are publicly available for research purposes through the CDC website (https://www.cdc.gov/nchs/nhanes/index.htm).

2. Study Population

A total of 43539 participants from the selected NHANES cycles were included in this study. The inclusion criteria were individuals aged 18 years and older with available data on HBV antibody status and SII-related biomarkers (platelet count, neutrophil count, and lymphocyte count). Additionally, participants with complete survival data,

as well as detailed information on covariates (such as age, sex, race/ethnicity, BMI, smoking history, diabetes, and hypertension), were included to ensure robust analysis. The exclusion criteria were as follows: (1) participants with missing data on HBV antibody testing, (2) individuals without information on SII biomarkers (platelet count, neutrophil count, and lymphocyte count), (3) those lacking critical covariate data, and (4) individuals with incomplete survival data, as accurate survival analysis requires complete and continuous follow-up information. This ensured that the analysis of the relationship between SII and HBV antibody status could be performed with minimal bias and more reliable results.

3. HBV Infection and Antibody Status

HBV antibody status was determined based on the detection of hepatitis B surface antibody (anti-HBs) in serum samples. Participants were classified as HBV antibody-positive (indicating past infection or successful vaccination) or HBV antibody-negative (indicating no detectable immunity or active infection). Serum samples were processed and stored following standard protocols by CDC-certified laboratories.

4. Systemic Immune–Inflammation Index (SII)

The SII was calculated using the following formula: SII = platelet count × neutrophil count / lymphocyte count. A higher SII value indicates a pro-tumor inflammatory state characterized by increased neutrophils and platelets and decreased lymphocytes.

5. Statistical Analysis

Descriptive statistics were calculated for all included variables. Continuous variables were expressed as means ± standard deviations, while categorical variables were presented as frequencies and percentages. Differences in clinical characteristics between HBV antibody-positive and negative groups were evaluated using independent t-tests for continuous variables and chi-square tests for categorical variables. To assess the relationship between SII and HBV antibody status, logistic regression analysis was employed. Three models were constructed: the unadjusted model (without covariate adjustment), the minimally adjusted model (adjusting for age, sex, race/ethnicity, BMI, smoking, diabetes, and hypertension), and the fully adjusted model (adjusting for all covariates, including dietary factors and other health behaviors). The association between SII and clinical outcomes, including liver function, was further evaluated using survival analysis techniques, such as Kaplan-Meier curves and Cox proportional hazards models.

3. Results

1. Patients inclusion:

This study included 43,539 patients, categorized into four groups based on systemic immune-inflammation index (SII) and hepatitis B surface antibody (HBsAb) status: high SII and HBsAb-negative (17,677), high SII and HBsAb-positive (4,090), low SII and HBsAb-negative (16,656), and low SII and HBsAb-positive (5,116). The mean age was 46.99 years, with older patients in the high SII groups (high SII/HBsAb-negative: 49.33 years) and younger patients in the low SII/HBsAb-positive group (39.80 years). Mean BMI was 28.80 kg/m², highest in the high SII/HBsAb-negative group (29.55 kg/m²) and lowest in the low SII/HBsAb-positive group (27.09 kg/m²). Lipid profiles showed higher triglycerides and total cholesterol in high SII groups, while HDL levels were higher in the low SII/HBsAb-positive group (1.42 mmol/L). Renal function, assessed by CKD-EPI eGFR, was lower in high SII groups (92.17 mL/min/1.73 m²) and higher in HBsAb-positive groups (100.73 mL/min/1.73 m²). Females predominated in the high SII/HBsAb-positive group (62.08%), while males were more common in the low SII/HBsAb-negative group (54.13%). Non-Hispanic Whites comprised the majority (69.81%), with higher diversity in the low SII/HBsAb-positive group. Education levels were higher in the high SII/HBsAb-positive group (69.63% above high school). Hypertension and CKD prevalence were higher in high SII groups, while COPD prevalence was lowest in the low SII/HBsAb-positive group (1.75%). In summary, high SII groups exhibited higher cardiometabolic risk profiles, whereas HBsAb-positive groups were younger with more favorable metabolic indicators. Details of inculded patients are shown in Table1.

2. SII and All-Cause Mortality

The association between SII and all-cause mortality was assessed across quartiles of SII. In the crude model, individuals in the highest SII quartile (Q4) had a significantly higher risk of all-cause mortality (HR = 1.339, 95% CI: 1.224–1.464, $p < 0.0001$) compared to those in the lowest quartile (Q1). After adjusting for age and sex (Model 1), the association remained significant (HR = 1.302, 95% CI: 1.207–1.405, $p < 0.0001$). Further adjustment for socio-economic and clinical factors in Models 2 and 3 did not substantially alter the results, with the HR for Q4 remaining elevated (HR = 1.318, 95% CI: 1.222–1.421, $p < 0.0001$ in Model 2 and HR = 1.138, 95% CI: 1.054–1.229, $p < 0.001$ in Model 3). This indicates that higher SII is independently associated with increased all-cause mortality risk. The detailed content above is provided in Table 2A.

3. SII and Cardiovascular Disease Mortality

The association between SII and cardiovascular disease (CVD) mortality was evaluated across quartiles of SII. In the crude model, participants in the highest SII

quartile (Q4) showed a significantly increased risk of cardiovascular mortality (HR = 1.575, 95% CI: 1.343–1.847, $p < 0.0001$) compared to those in the lowest quartile (Q1). After adjusting for age and sex (Model 1), the risk remained elevated (HR = 1.590, 95% CI: 1.367–1.851, $p < 0.0001$). Further adjustments for additional socio-economic and clinical factors in Models 2 and 3 did not change the association substantially, with the HR for Q4 remaining significant (HR = 1.655, 95% CI: 1.415–1.936, $p < 0.0001$ in Model 2 and HR = 1.402, 95% CI: 1.191–1.649, $p < 0.0001$ in Model 3). This suggests that higher SII is independently associated with increased cardiovascular mortality risk. The detailed content above is provided in Table 2B.The all-cause mortality, CVD death, and non-CVD death associated with the high and low SII groups are presented in Figure 1a, 1b, and 1c, respectively.

4. HBV Surface Antibody and All-Cause Mortality

For HBV surface antibody status, participants who were HBV antibody-positive had a significantly lower risk of all-cause mortality in the crude model (HR = 0.491, 95% CI: 0.441–0.547, $p < 0.0001$). However, after adjusting for age and sex in Model 1, the HR increased and became non-significant (HR = 0.909, 95% CI: 0.819–1.009, p = 0.072). Further adjustment in Models 2 and 3 continued to show no significant association (HR = 0.878, 95% CI: 0.788–0.978, p = 0.018 in Model 2 and HR = 0.906, 95% CI: 0.809–1.014, p = 0.086 in Model 3). This suggests that while HBV antibody positivity may initially appear protective, this effect is diminished after controlling for other factors. The detailed content above is provided in Table 3A.

5. HBV Surface Antibody and Cardiovascular Disease Mortality

For HBV surface antibody status, the crude model indicated a significant protective effect of being HBV antibody-positive, with a lower hazard ratio for cardiovascular mortality (HR = 0.478, 95% CI: 0.397–0.576, $p < 0.0001$) compared to the antibody-negative group. However, after adjusting for age and sex in Model 1, the HR increased and became non-significant (HR = 0.981, 95% CI: 0.827–1.164, p = 0.829). Further adjustments in Models 2 and 3 continued to show a non-significant association (HR = 0.920, 95% CI: 0.773–1.095, p = 0.347 in Model 2 and HR = 0.946, 95% CI: 0.788–1.135, p = 0.551 in Model 3). This suggests that HBV antibody positivity may have an initial protective effect, but this association is attenuated after adjusting for other factors. The detailed content above is provided in Table 3B. The all-cause mortality, CVD death, and non-CVD death associated with HBV antibody-negative and -positive groups are presented in Figure 2a, 2b, and 2c, respectively.

6. Combined Effect of SII and HBV Surface Antibody on All-Cause Mortality

When examining the combined effect of SII and HBV surface antibody status on all-cause mortality, a strong trend emerged for individuals with high SII and HBV antibody-negative status. In the crude model, the HR for this group was 1.575 (95% CI: 1.343–1.847, $p < 0.0001$), and it remained significant after adjusting for age and sex (HR = 1.590, 95% CI: 1.367–1.851, $p < 0.0001$). Further adjustments in Models 2 and 3 confirmed that high SII continued to be associated with significantly higher all-cause mortality risk (HR = 1.655, 95% CI: 1.415–1.936, $p < 0.0001$ in Model 2 and HR = 1.402, 95% CI: 1.191–1.649, $p < 0.0001$ in Model 3). The detailed content above is provided in Table 4A.

7. Combined Effect of SII and HBV Surface Antibody on Cardiovascular Disease Mortality

When examining the combined effect of SII and HBV surface antibody status on cardiovascular mortality, a clear trend emerged. Participants with high SII and HBV antibody-negative status exhibited the worst survival outcomes. In the crude model, the HR for this group was 1.575 (95% CI: 1.343–1.847, $p < 0.0001$), and after adjusting for age and sex, the HR remained significant (HR = 1.590, 95% CI: 1.367–1.851, $p < 0.0001$). Further adjustments in Models 2 and 3 confirmed the strong association between high SII and poor cardiovascular outcomes, with the HR for Q4 consistently higher than for the lower SII groups. This indicates that while HBV antibody positivity might provide some protection, the risk conferred by high SII on cardiovascular mortality remains significant. The detailed content above is provided in Table 4B. The all-cause mortality, CVD death, and non-CVD death associated with the combined SII and HBV antibody status groups are presented in Figure 3a, 3b, and 3c, respectively.

4. Discussion

In this study, we examined the relationship between the systemic immune-inflammation index (SII), HBV surface antibody status, and their combined effects on all-cause and cardiovascular disease (CVD) mortality. Our results suggest that both SII and HBV surface antibody status are significant predictors of mortality, with high SII associated with increased risks, particularly for all-cause and cardiovascular mortality. However, the protective effect of HBV surface antibody positivity diminished after adjusting for other clinical and sociodemographic factors(17), indicating that while there may be an initial protective effect, it is not robust when considering other health factors(18, 19).

Our findings confirm previous research highlighting the importance of inflammatory markers in predicting mortality. SII, which integrates platelet, neutrophil, and lymphocyte counts, serves as a novel marker of systemic inflammation and immune

status. Elevated SII has been shown to correlate with worse outcomes in various diseases, including cancer and cardiovascular conditions(20). In our study, participants with higher SII (particularly in the fourth quartile) had significantly higher risks of both all-cause and CVD mortality, even after adjusting for a wide range of potential confounders. This supports the hypothesis that SII could serve as an effective biomarker for identifying inflammation-driven mortality risks in the general population(21).

HBV surface antibody positivity has traditionally been viewed as a marker of immunity against hepatitis B virus infection(22). Initially, our results showed that HBV antibody positivity was associated with lower risks of all-cause and cardiovascular mortality in the crude models. However, after adjusting for sociodemographic factors, comorbidities, and lifestyle factors, this protective association diminished and became non-significant in the final models. These findings suggest that while HBV surface antibodies may confer some protection against infection-related outcomes, their role in broader mortality outcomes is less pronounced once other health factors are accounted for. This aligns with previous studies that suggest the protective effect of HBV antibodies may be limited to preventing acute infections, rather than influencing chronic disease outcomes like cardiovascular events(23, 24).

The combined effect of SII and HBV surface antibody status revealed a more complex relationship(25). Participants with high SII and HBV antibody-negative status had the highest mortality risks across all models, emphasizing the detrimental effects of both systemic inflammation and the absence of immunity to HBV on health outcomes. This suggests that individuals with high levels of inflammation and no prior immunity to HBV may be at particularly elevated risk for poor outcomes. Conversely, those with high SII but HBV antibody-positive status had a moderately lower risk, suggesting that prior vaccination or immunity could attenuate the negative effects of systemic inflammation(18).

This finding underscores the importance of considering both immune status and inflammation when evaluating mortality risk(26). While SII provides insight into systemic inflammation, HBV surface antibody status reflects an individual's immune history and protective status against viral infections(27). By combining these two markers, we gain a more nuanced understanding of how immune and inflammatory responses contribute to overall mortality, including cardiovascular outcomes.

The results of this study have several clinical implications. First, SII could serve as a useful biomarker for identifying individuals at higher risk of mortality, particularly in populations with high levels of inflammation(28). Second, while HBV vaccination remains a crucial public health measure(29), our findings suggest that the protective effects of HBV surface antibody positivity on mortality may be limited in the presence of other risk factors, such as inflammation and comorbidities. Therefore, a

more comprehensive approach to risk stratification, incorporating both inflammatory markers and immune status, may be necessary for better predicting mortality risks.

This study has several limitations. First, the observational design prevents us from establishing causality between SII, HBV surface antibody status, and mortality outcomes. Second, while we controlled for a wide range of covariates, residual confounding may still exist. Additionally, the use of NHANES data, while comprehensive, may not fully capture all relevant factors influencing mortality, such as detailed medical histories or specific treatment interventions. Lastly, while we adjusted for key variables in statistical models, potential biases related to missing data or unmeasured confounders cannot be fully excluded. Future studies should explore the mechanistic pathways linking inflammation, immune status, and mortality(30). Longitudinal studies that track changes in SII and HBV antibody status over time may help clarify their dynamic roles in predicting long-term health outcomes. Moreover, research into interventions aimed at reducing inflammation, such as lifestyle modifications or pharmacological treatments, could provide promising strategies for mitigating the mortality risks associated with elevated SII levels.

5. Conclusions

Our study highlights the significant role of the Systemic Immune-Inflammation Index (SII) and hepatitis B antibody status in predicting mortality risk. Elevated SII, in combination with negative HBV antibody status, indicates a higher risk for poor health outcomes, including increased all-cause and cardiovascular mortality. On the other hand, a positive HBV antibody status seems to mitigate some of the negative effects of systemic inflammation. These findings underscore the importance of considering both immune and inflammatory markers when assessing long-term health risks, offering potential for improved patient risk stratification and targeted interventions.

Declarations

Consent for publication
All authors unanimously agreed to submit this manuscript for publication.

Availability of data and materials
All data generated or analyzed during this study are included in this published article. All figures in our study are original and have not been previously published.

Consent for publication
Not applicable.

Human Ethics and Consent to Participate declarations:

Not applicable.

Ethics Declaration

This study utilized data from the National Health and Nutrition Examination Survey (NHANES), a publicly available dataset provided by the Centers for Disease Control and Prevention (CDC). All data were anonymized prior to analysis, and NHANES participants provided informed consent at the time of data collection. The NHANES protocol is reviewed and approved by the National Center for Health Statistics (NCHS) Research Ethics Review Board, and all procedures are conducted in accordance with the Declaration of Helsinki.

Competing interests

The authors declare that they have no competing interests. The authors are accountable for all aspects of the work in ensuring that questions related to the accuracy or integrity of any part of the work are appropriately investigated and resolved.

Conflict-of-interest statement:

All authors have no conflicts of interest regarding the current paper.

Author contributions:

Di Zeng: Conceptualization (lead); Data curation (lead); Formal analysis (lead); Investigation (lead); Methodology (lead); Writing – original draft (lead); Writing – review & editing (lead).**Shaofeng Wang** : Conceptualization (equal); Formal analysis (equal); Investigation (equal); Writing – review & editing (equal). **Nansheng Cheng**: Investigation (equal); Writing – review & editing (equal); Methodology (lead); Supervision (equal). **Bei Li:** Supervision (lead); Writing – review & editing (lead).

Funding

This study did not receive any funding.

Acknowledgements

Not applicable.

Abbreviation List

Anti-HBs - Hepatitis B Surface Antibody

BMI - Body Mass Index

CVD - Cardiovascular Disease

HBV - Hepatitis B Virus

HCC - Hepatocellular Carcinoma

NAFLD - Non-Alcoholic Fatty Liver Disease

SII - Systemic Immune-Inflammation Index

TCGA - The Cancer Genome Atlas

OS - Overall Survival

DFS - Disease-Free Survival

CDC - Centers for Disease Control and Prevention

HR - Hazard Ratio

CI - Confidence Interval

WH0 - World Health Organization

PLR - Platelet-to-Lymphocyte Ratio

NLR - Neutrophil-to-Lymphocyte Ratio

RBC - Red Blood Cell

WBC - White Blood Cell

CRP - C-Reactive Protein

Figure Legends

Figure 1
Figure 1a: All-cause mortality associated with high and low systemic immune-inflammation index (SII) groups.

Figure 1b: Cardiovascular disease (CVD) mortality associated with high and low systemic immune-inflammation index (SII) groups.

Figure 1c: Non-cardiovascular disease (non-CVD) mortality associated with high and low systemic immune-inflammation index (SII) groups.

Figure 2

Figure 2a: All-cause mortality associated with hepatitis B surface antibody (HBsAb)-negative and HBsAb-positive groups.

Figure 2b: Cardiovascular disease (CVD) mortality associated with hepatitis B surface antibody (HBsAb)-negative and HBsAb-positive groups.

Figure 2c: Non-cardiovascular disease (non-CVD) mortality associated with hepatitis B surface antibody (HBsAb)-negative and HBsAb-positive groups.

Figure 3

Figure 3a: All-cause mortality associated with combined systemic immune-inflammation index (SII) and hepatitis B surface antibody (HBsAb) status groups.

Figure 3b: Cardiovascular disease (CVD) mortality associated with combined systemic immune-inflammation index (SII) and hepatitis B surface antibody (HBsAb) status groups.

Figure 3c: Non-cardiovascular disease (non-CVD) mortality associated with combined systemic immune-inflammation index (SII) and hepatitis B surface antibody (HBsAb) status groups.

Table Legends

Table 1

Table 1: Baseline characteristics of the study population stratified by systemic immune-inflammation index (SII) and hepatitis B surface antibody (HBsAb) status.

Table 2

Table 2A: Hazard ratios (HRs) and 95% confidence intervals (CIs) for all-cause mortality across quartiles of systemic immune-inflammation index (SII).

Table 2B: Hazard ratios (HRs) and 95% confidence intervals (CIs) for cardiovascular disease (CVD) mortality across quartiles of systemic immune-inflammation index (SII).

Table 3

Table 3A: Hazard ratios (HRs) and 95% confidence intervals (CIs) for all-cause mortality by hepatitis B surface antibody (HBsAb) status.

Table 3B: Hazard ratios (HRs) and 95% confidence intervals (CIs) for cardiovascular disease (CVD) mortality by hepatitis B surface antibody (HBsAb) status.

Table 4

Table 4A: Hazard ratios (HRs) and 95% confidence intervals (CIs) for all-cause mortality by combined systemic immune-inflammation index (SII) and hepatitis B surface antibody (HBsAb) status.

Table 4B: Hazard ratios (HRs) and 95% confidence intervals (CIs) for cardiovascular disease (CVD) mortality by combined systemic immune-inflammation index (SII) and hepatitis B surface antibody (HBsAb) status.

References:

1. Global, regional, and national burden of hepatitis B, 1990-2019: a systematic analysis for the Global Burden of Disease Study 2019. Lancet Gastroenterol Hepatol. 2022;7(9):796-829. Epub 20220621. doi: 10.1016/s2468-1253(22)00124-8. PubMed PMID: 35738290; PubMed Central PMCID: PMC9349325.
2. Hepatitis B vaccines: WHO position paper--recommendations. Vaccine. 2010;28(3):589-90. Epub 20091105. doi: 10.1016/j.vaccine.2009.10.110. PubMed PMID: 19896455.
3. El-Serag HB. Epidemiology of viral hepatitis and hepatocellular carcinoma. Gastroenterology. 2012;142(6):1264-73.e1. doi: 10.1053/j.gastro.2011.12.061. PubMed PMID: 22537432; PubMed Central PMCID: PMC3338949.
4. Mitchell T, Nayagam JS, Dusheiko G, Agarwal K. Health inequalities in the management of chronic hepatitis B virus infection in patients from sub-Saharan Africa in high-income countries. JHEP Rep. 2023;5(2):100623. Epub 20221104. doi: 10.1016/j.jhepr.2022.100623. PubMed PMID: 36636709; PubMed Central PMCID: PMC9829705.
5. Nelson NP, Easterbrook PJ, McMahon BJ. Epidemiology of Hepatitis B Virus Infection and Impact of Vaccination on Disease. Clin Liver Dis. 2016;20(4):607-28. doi: 10.1016/j.cld.2016.06.006. PubMed PMID: 27742003; PubMed Central PMCID: PMC5582972.
6. Varghese N, Majeed A, Nyalakonda S, Boortalary T, Halegoua-DeMarzio D, Hann HW. Review of Related Factors for Persistent Risk of Hepatitis B Virus-Associated Hepatocellular Carcinoma. Cancers (Basel). 2024;16(4). Epub 20240214. doi: 10.3390/cancers16040777. PubMed PMID: 38398168; PubMed Central PMCID: PMC10887172.
7. Hu B, Yang XR, Xu Y, Sun YF, Sun C, Guo W, et al. Systemic immune-inflammation index predicts prognosis of patients after curative resection for hepatocellular carcinoma. Clin Cancer Res. 2014;20(23):6212-22. Epub 20140930. doi: 10.1158/1078-0432.Ccr-14-0442. PubMed PMID: 25271081.
8. Liu J, Li S, Zhang S, Liu Y, Ma L, Zhu J, et al. Systemic immune-inflammation index, neutrophil-to-lymphocyte ratio, platelet-to-lymphocyte ratio can predict clinical outcomes in patients with metastatic non-small-cell lung cancer treated with nivolumab. J Clin Lab Anal. 2019;33(8):e22964. Epub 20190708. doi: 10.1002/jcla.22964. PubMed PMID: 31282096; PubMed Central PMCID: PMC6805305.
9. Fest J, Ruiter R, Mulder M, Groot Koerkamp B, Ikram MA, Stricker BH, van Eijck CHJ. The systemic immune-inflammation index is associated with an increased risk of incident cancer-A population-based cohort study. Int J Cancer. 2020;146(3):692-8. Epub 20190416. doi: 10.1002/ijc.32303. PubMed PMID: 30924141; PubMed Central PMCID: PMC6916270.
10. Qiu Y, Zhang Z, Chen Y. Prognostic Value of Pretreatment Systemic Immune-Inflammation Index in

Gastric Cancer: A Meta-Analysis. Front Oncol. 2021;11:537140. Epub 20210311. doi: 10.3389/fonc.2021.537140. PubMed PMID: 33777726; PubMed Central PMCID: PMC7990885.
11. Menyhart O, Fekete JT, Győrffy B. Inflammation and Colorectal Cancer: A Meta-Analysis of the Prognostic Significance of the Systemic Immune-Inflammation Index (SII) and the Systemic Inflammation Response Index (SIRI). Int J Mol Sci. 2024;25(15). Epub 20240802. doi: 10.3390/ijms25158441. PubMed PMID: 39126008; PubMed Central PMCID: PMC11312822.
12. Wu Y, Zhao J, Wang Z, Liu D, Tian C, Ye B, et al. Association of systemic inflammatory markers and tertiary lymphoid structure with pathological complete response in gastric cancer patients receiving preoperative treatment: a retrospective cohort study. Int J Surg. 2023;109(12):4151-61. Epub 20231201. doi: 10.1097/js9.0000000000000741. PubMed PMID: 38259000; PubMed Central PMCID: PMC10720847.
13. Gao Y, Guo W, Cai S, Zhang F, Shao F, Zhang G, et al. Systemic immune-inflammation index (SII) is useful to predict survival outcomes in patients with surgically resected esophageal squamous cell carcinoma. J Cancer. 2019;10(14):3188-96. Epub 20190602. doi: 10.7150/jca.30281. PubMed PMID: 31289589; PubMed Central PMCID: PMC6603384.
14. Tan A, Koh S, Bertoletti A. Immune Response in Hepatitis B Virus Infection. Cold Spring Harb Perspect Med. 2015;5(8):a021428. Epub 20150701. doi: 10.1101/cshperspect.a021428. PubMed PMID: 26134480; PubMed Central PMCID: PMC4526720.
15. Stasi C, Silvestri C, Voller F. Hepatitis B vaccination and immunotherapies: an update. Clin Exp Vaccine Res. 2020;9(1):1-7. Epub 20200131. doi: 10.7774/cevr.2020.9.1.1. PubMed PMID: 32095435; PubMed Central PMCID: PMC7024731.
16. Wu S, Yi W, Gao Y, Deng W, Bi X, Lin Y, et al. Immune Mechanisms Underlying Hepatitis B Surface Antigen Seroclearance in Chronic Hepatitis B Patients With Viral Coinfection. Front Immunol. 2022;13:893512. Epub 20220511. doi: 10.3389/fimmu.2022.893512. PubMed PMID: 35634301; PubMed Central PMCID: PMC9130599.
17. Paul S, Dickstein A, Saxena A, Terrin N, Viveiros K, Balk EM, Wong JB. Role of surface antibody in hepatitis B reactivation in patients with resolved infection and hematologic malignancy: A meta-analysis. Hepatology. 2017;66(2):379-88. Epub 20170622. doi: 10.1002/hep.29082. PubMed PMID: 28128861; PubMed Central PMCID: PMC6485929.
18. Heidrich B, Deterding K, Tillmann HL, Raupach R, Manns MP, Wedemeyer H. Virological and clinical characteristics of delta hepatitis in Central Europe. J Viral Hepat. 2009;16(12):883-94. Epub 20090628. doi: 10.1111/j.1365-2893.2009.01144.x. PubMed PMID: 19566789.
19. Colagrossi L, Salpini R, Scutari R, Carioti L, Battisti A, Piermatteo L, et al. HDV Can Constrain HBV Genetic Evolution in HBsAg: Implications for the Identification of Innovative Pharmacological Targets. Viruses. 2018;10(7). Epub 20180709. doi: 10.3390/v10070363. PubMed PMID: 29987240; PubMed Central PMCID: PMC6071122.
20. Wu S, Liu Z, Li X, Gao S, Xia P. Association between systemic immune-inflammation index and the risk of all-cause, cancer and non-cancer mortality in the general population: results from national health and nutrition examination survey 2005-2018. BMC Public Health. 2025;25(1):227. Epub 20250120. doi: 10.1186/s12889-025-21423-1. PubMed PMID: 39833806; PubMed Central PMCID: PMC11744828.
21. Sun W, Fang Y, Zhou B, Mao G, Cheng J, Zhang X, et al. The association of systemic inflammatory biomarkers with non-alcoholic fatty liver disease: a large population-based cross-sectional study. Prev Med Rep. 2024;37:102536. Epub 20231207. doi: 10.1016/j.pmedr.2023.102536. PubMed PMID:

38186662; PubMed Central PMCID: PMC10767190.
22. Song JE, Kim DY. Diagnosis of hepatitis B. Annals of Translational Medicine. 2016;4(18):338.
23. Heidrich B, Serrano BC, Idilman R, Kabaçam G, Bremer B, Raupach R, et al. HBeAg-positive hepatitis delta: virological patterns and clinical long-term outcome. Liver Int. 2012;32(9):1415-25. Epub 20120620. doi: 10.1111/j.1478-3231.2012.02831.x. PubMed PMID: 22716112.
24. Martin-Gayo E, Buzon MJ, Ouyang Z, Hickman T, Cronin J, Pimenova D, et al. Potent Cell-Intrinsic Immune Responses in Dendritic Cells Facilitate HIV-1-Specific T Cell Immunity in HIV-1 Elite Controllers. PLoS Pathog. 2015;11(6):e1004930. Epub 20150611. doi: 10.1371/journal.ppat.1004930. PubMed PMID: 26067651; PubMed Central PMCID: PMC4466270.
25. Bertoletti A, Ferrari C. Kinetics of the immune response during HBV and HCV infection. Hepatology. 2003;38(1):4-13. doi: 10.1053/jhep.2003.50310. PubMed PMID: 12829979.
26. Quirino A, Marascio N, Branda F, Ciccozzi A, Romano C, Locci C, et al. Viral Hepatitis: Host Immune Interaction, Pathogenesis and New Therapeutic Strategies. Pathogens. 2024;13(9). Epub 20240905. doi: 10.3390/pathogens13090766. PubMed PMID: 39338957; PubMed Central PMCID: PMC11435051.
27. Ding L, Huang J, Huang S. The significance of antibody to hepatitis B surface antigen in infection and clearance of hepatitis B virus. Hum Vaccin Immunother. 2025;21(1):2445283. Epub 20250103. doi: 10.1080/21645515.2024.2445283. PubMed PMID: 39754388.
28. Tang Y, Feng X, Liu N, Zhou Y, Wang Y, Chen Z, Liu Y. Relationship between systemic immune inflammation index and mortality among US adults with different diabetic status: Evidence from NHANES 1999-2018. Exp Gerontol. 2024;185:112350. Epub 20231222. doi: 10.1016/j.exger.2023.112350. PubMed PMID: 38128848.
29. Al-Busafi SA, Alwassief A. Global Perspectives on the Hepatitis B Vaccination: Challenges, Achievements, and the Road to Elimination by 2030. Vaccines (Basel). 2024;12(3). Epub 20240309. doi: 10.3390/vaccines12030288. PubMed PMID: 38543922; PubMed Central PMCID: PMC10975970.
30. Chen L, Deng H, Cui H, Fang J, Zuo Z, Deng J, et al. Inflammatory responses and inflammation-associated diseases in organs. Oncotarget. 2018;9(6):7204-18. Epub 20171214. doi: 10.18632/oncotarget.23208. PubMed PMID: 29467962; PubMed Central PMCID: PMC5805548.

Figure All_cause_mortality Click here to access/download;Figure;Figure1a.pdf

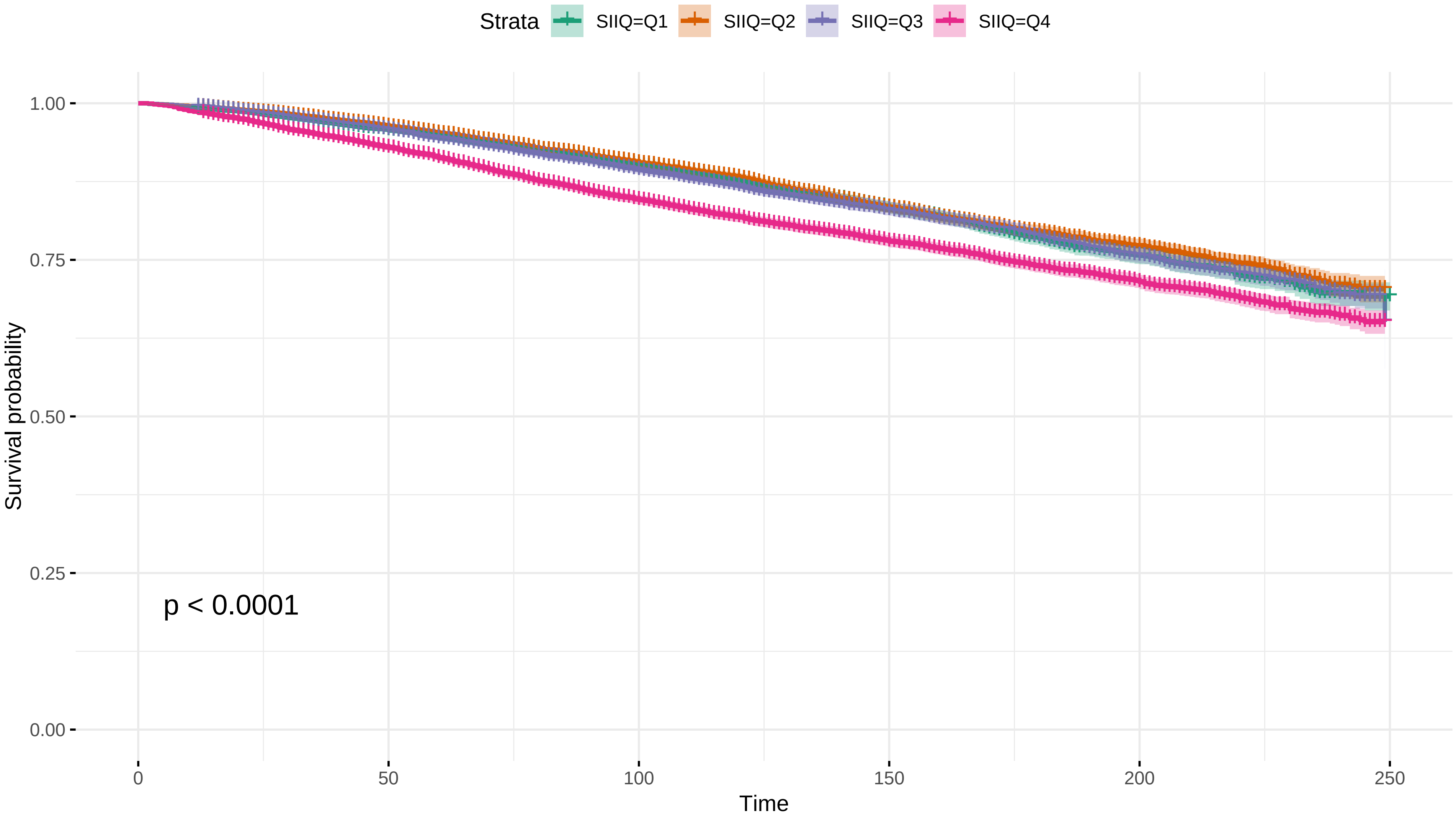

All_cause_mortality

Strata	0	50	100	150	200	250
SIIQ=Q1	10885	8585	5433	2744	1258	1
SIIQ=Q2	10887	8950	6107	3351	1538	0
SIIQ=Q3	10883	9049	6412	3748	1715	0
SIIQ=Q4	10884	9027	6579	4182	1938	0

Time

Figure

CVD_cause_mortality

Click here to access/download;Figure;Figure1b.pdf

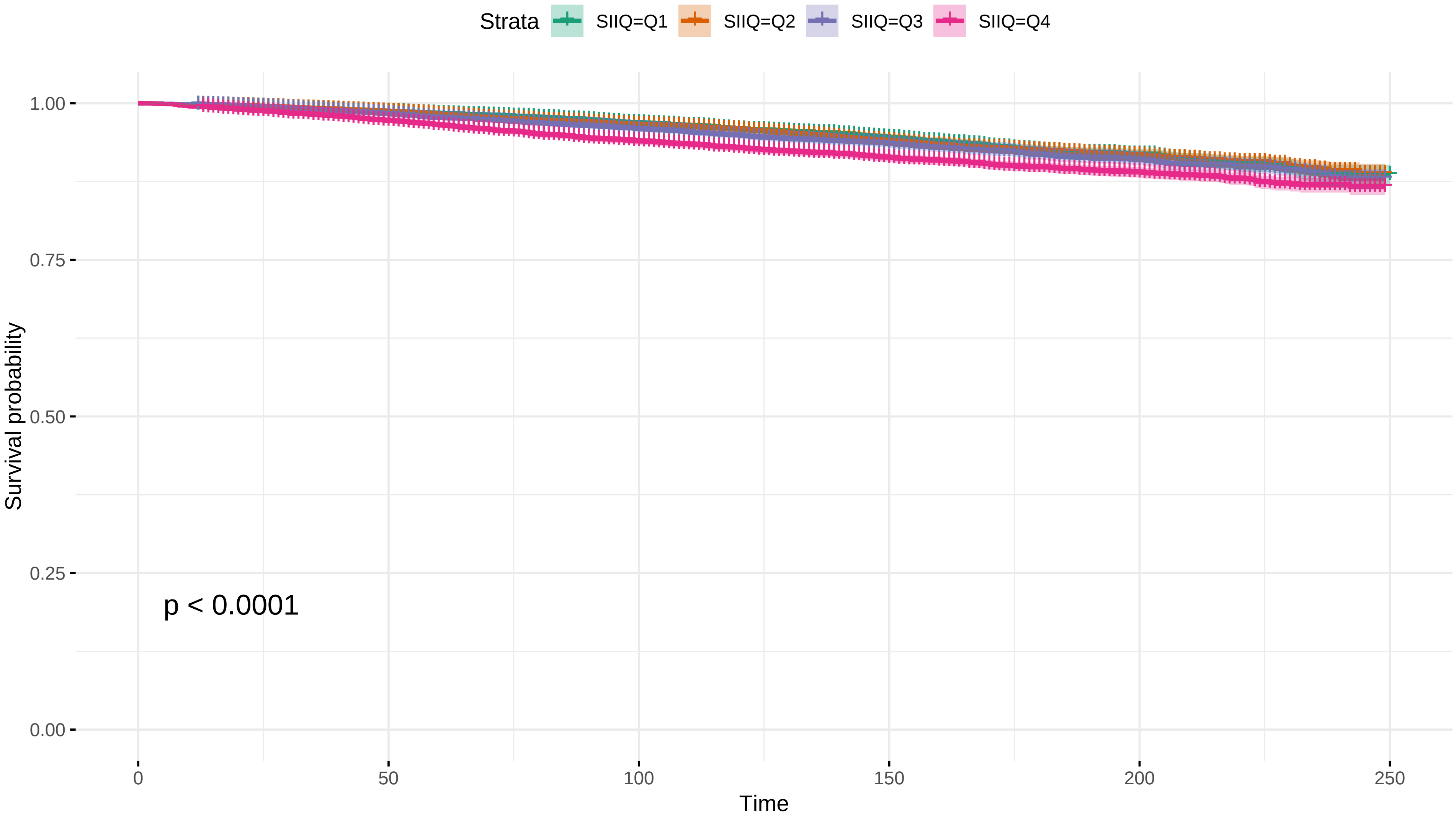

CVD_cause_mortality

Strata	0	50	100	150	200	250
SIIQ=Q1	9885	7886	5039	2569	1221	1
SIIQ=Q2	9877	8187	5660	3171	1489	0
SIIQ=Q3	9767	8205	5926	3527	1662	0
SIIQ=Q4	9357	7984	6029	3933	1873	0

Time

Figure non_CVD_cause_mortality

Click here to access/download;Figure;Figure1c.pdf

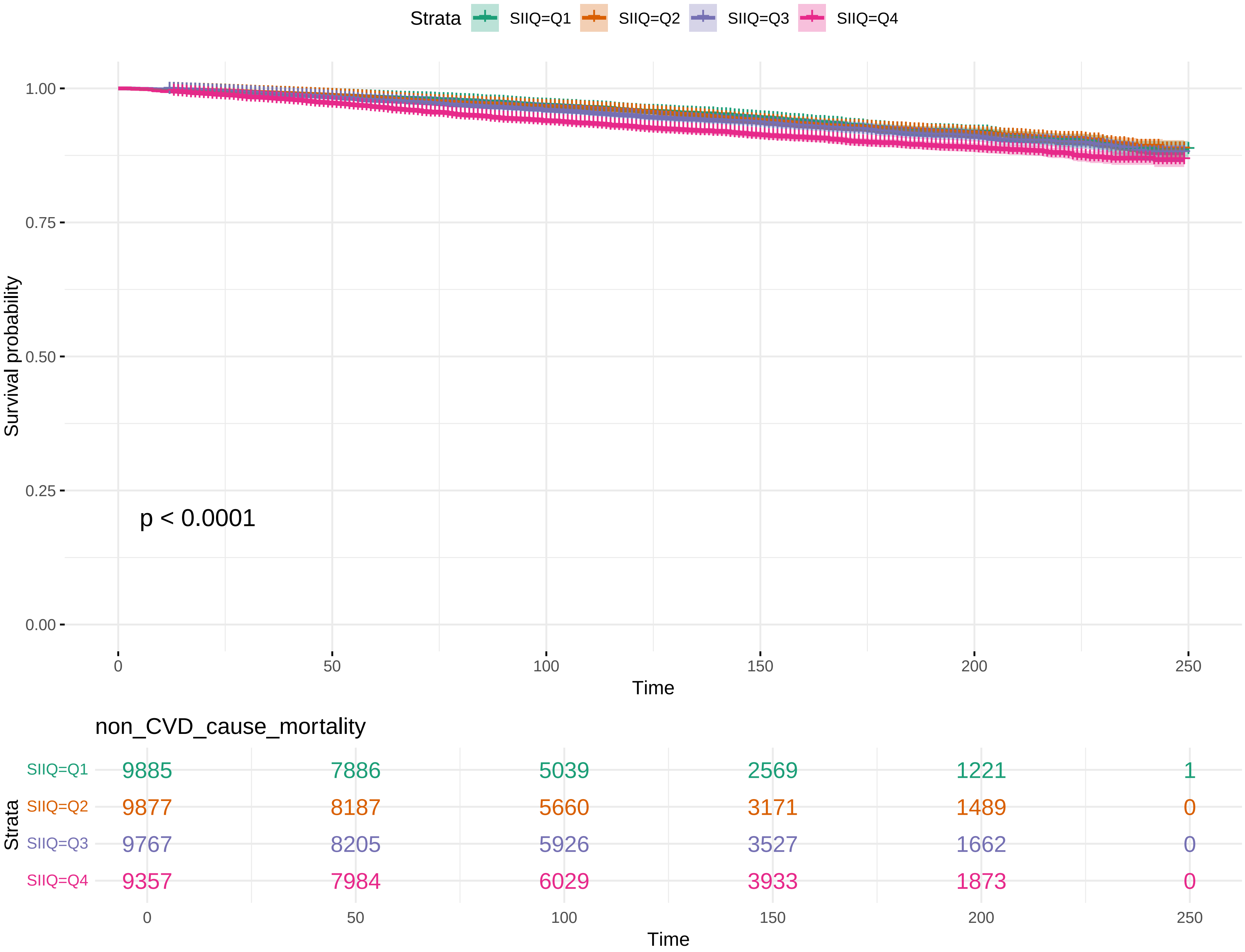

Figure All_cause_mortality

Click here to access/download;Figure;Figure2a.pdf

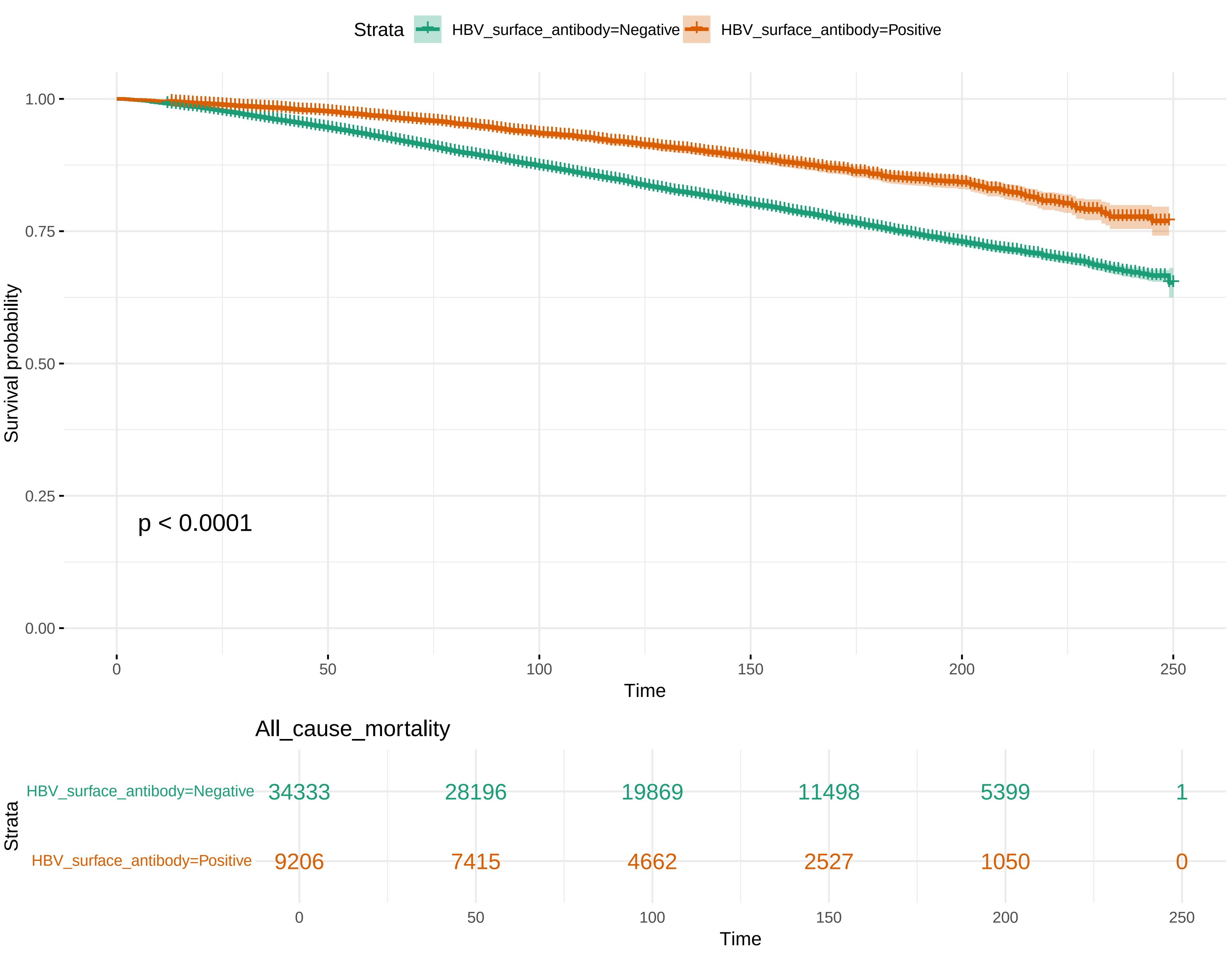

Figure

Click here to access/download;Figure;Figure2b.pdf

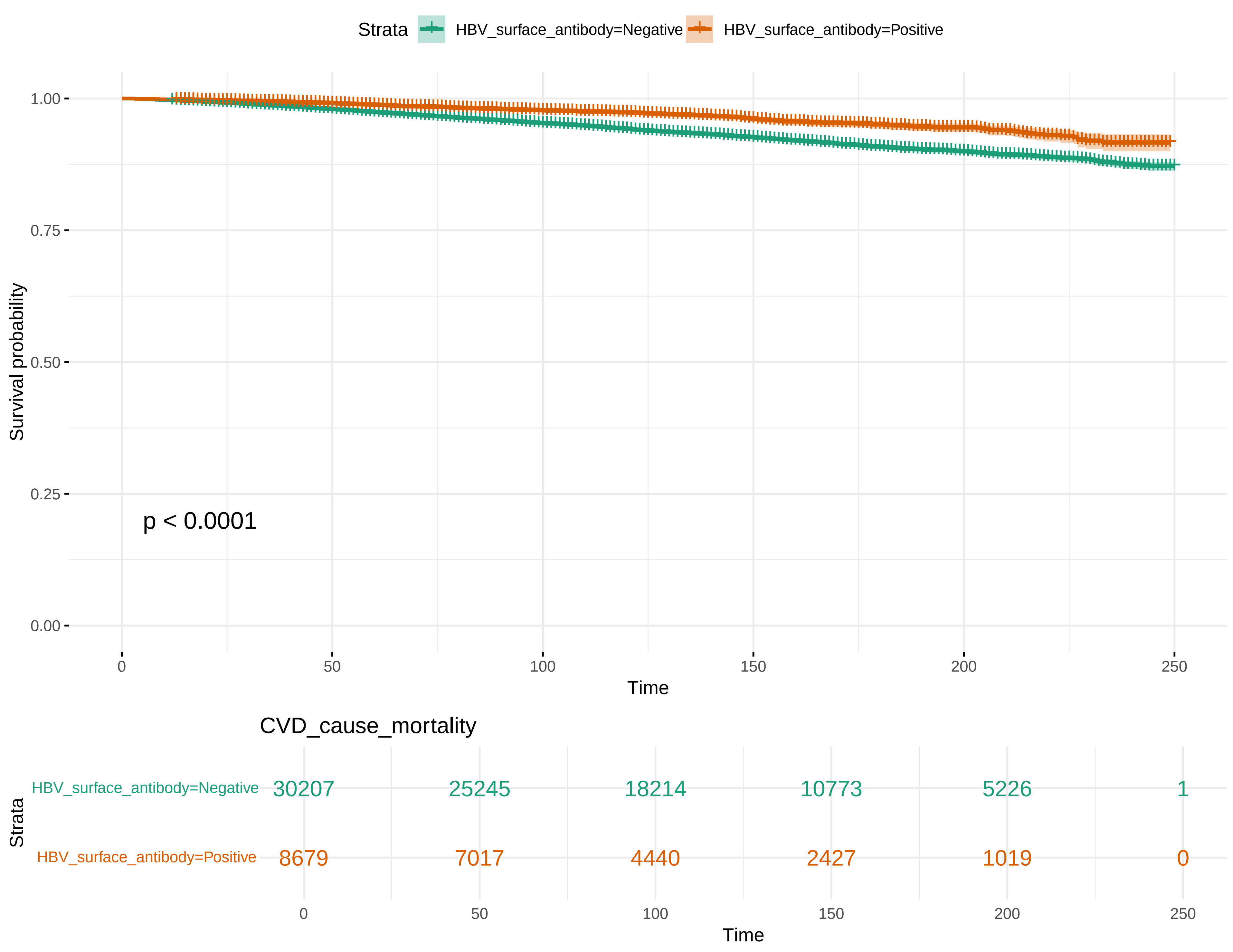

Figure non_CVD_cause_mortality

Click here to access/download;Figure;Figure2c.pdf

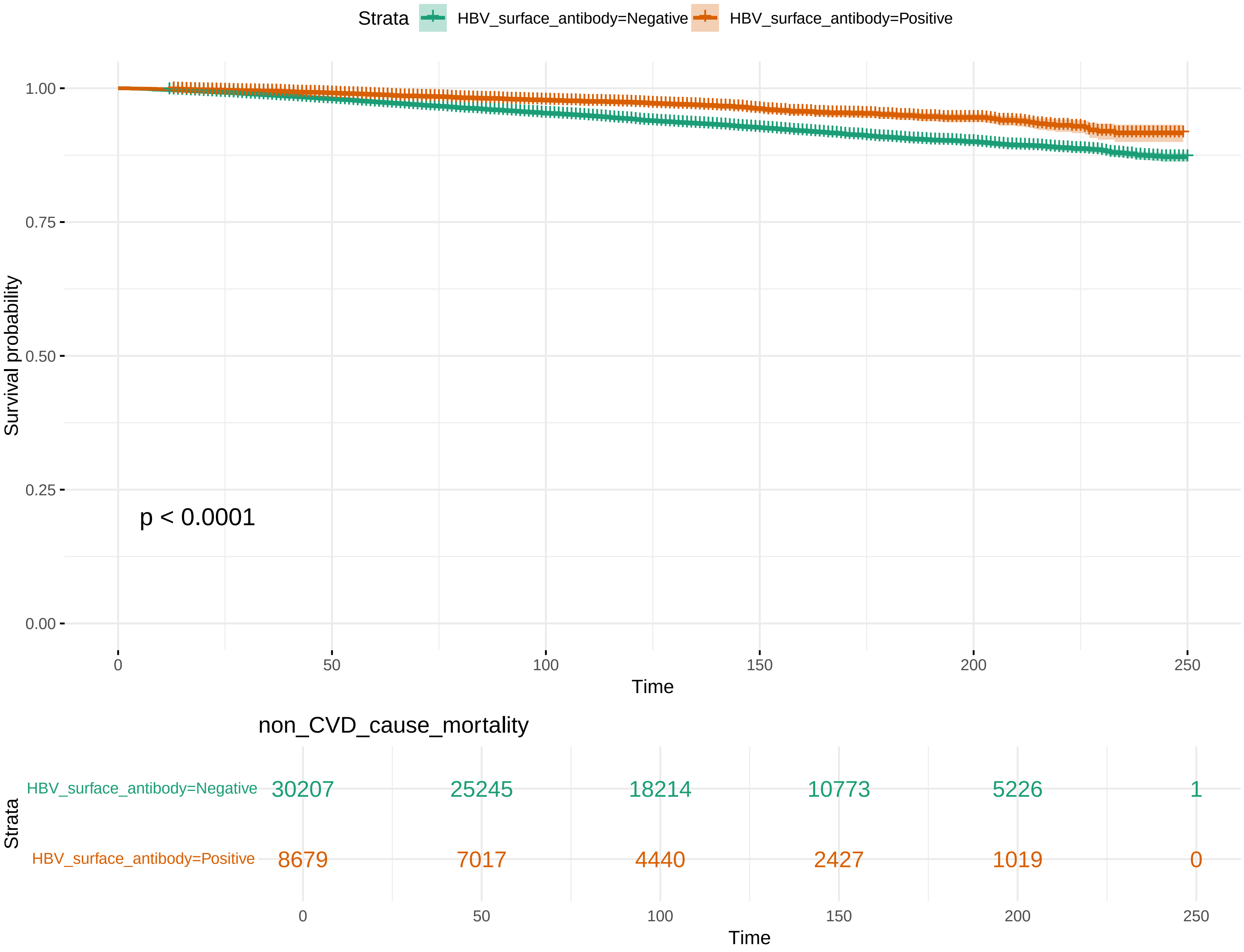

Figure All_cause_mortality

Click here to access/download;Figure;Figure3a.pdf

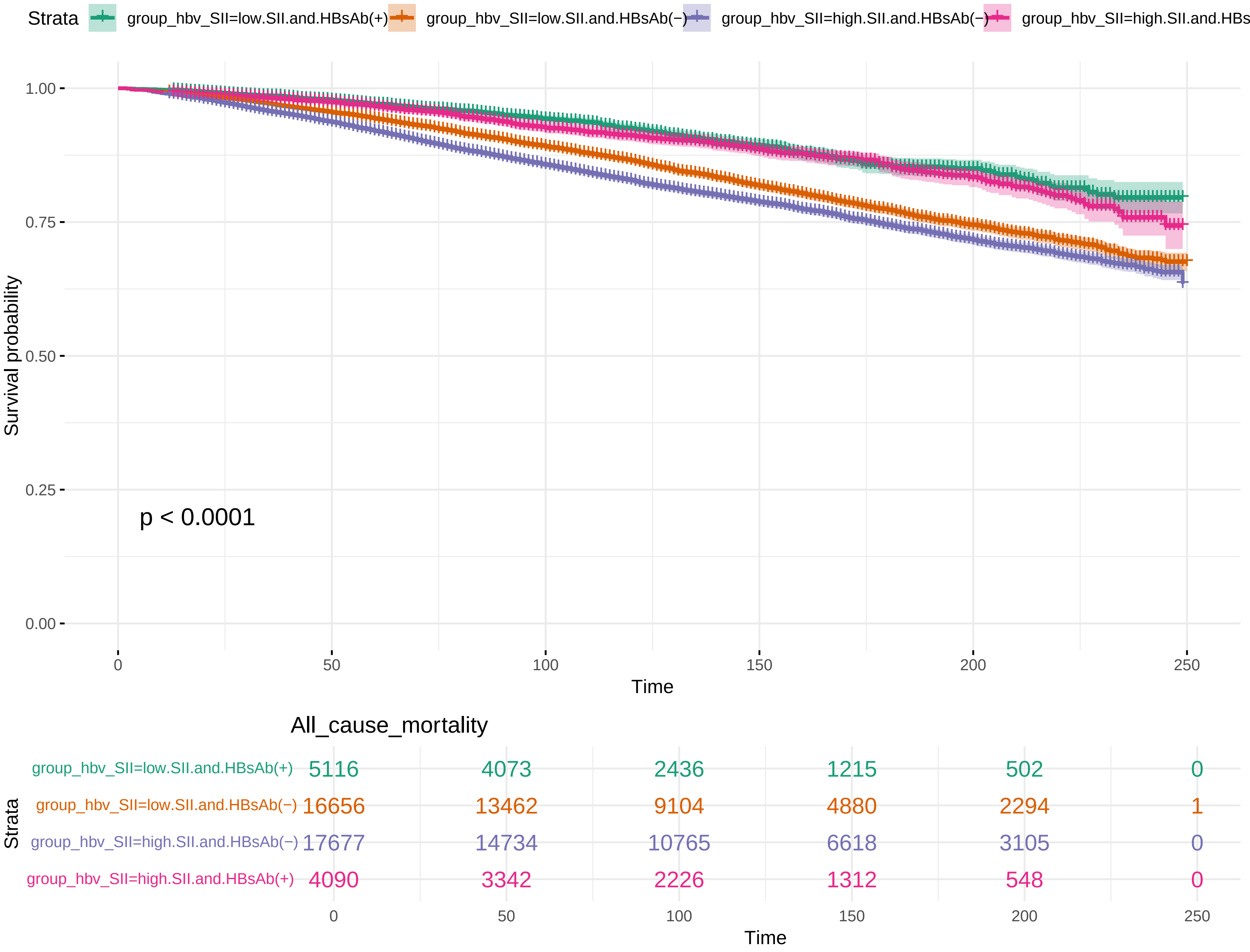

Figure CVD_cause_mortality

Click here to access/download;Figure;Figure3b.pdf

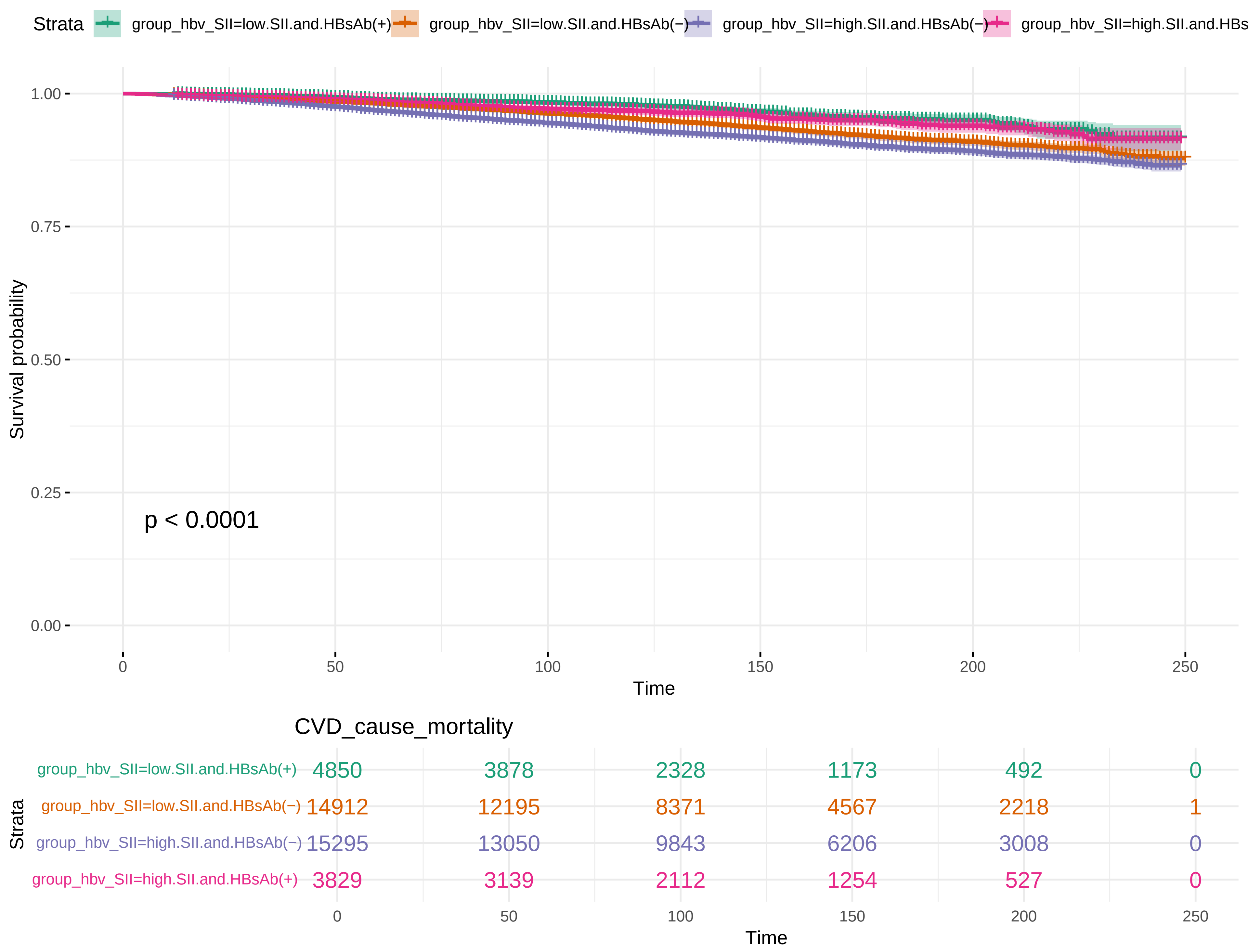

Figure
Click here to access/download;Figure;Figure3c.pdf

non_CVD_cause_mortality

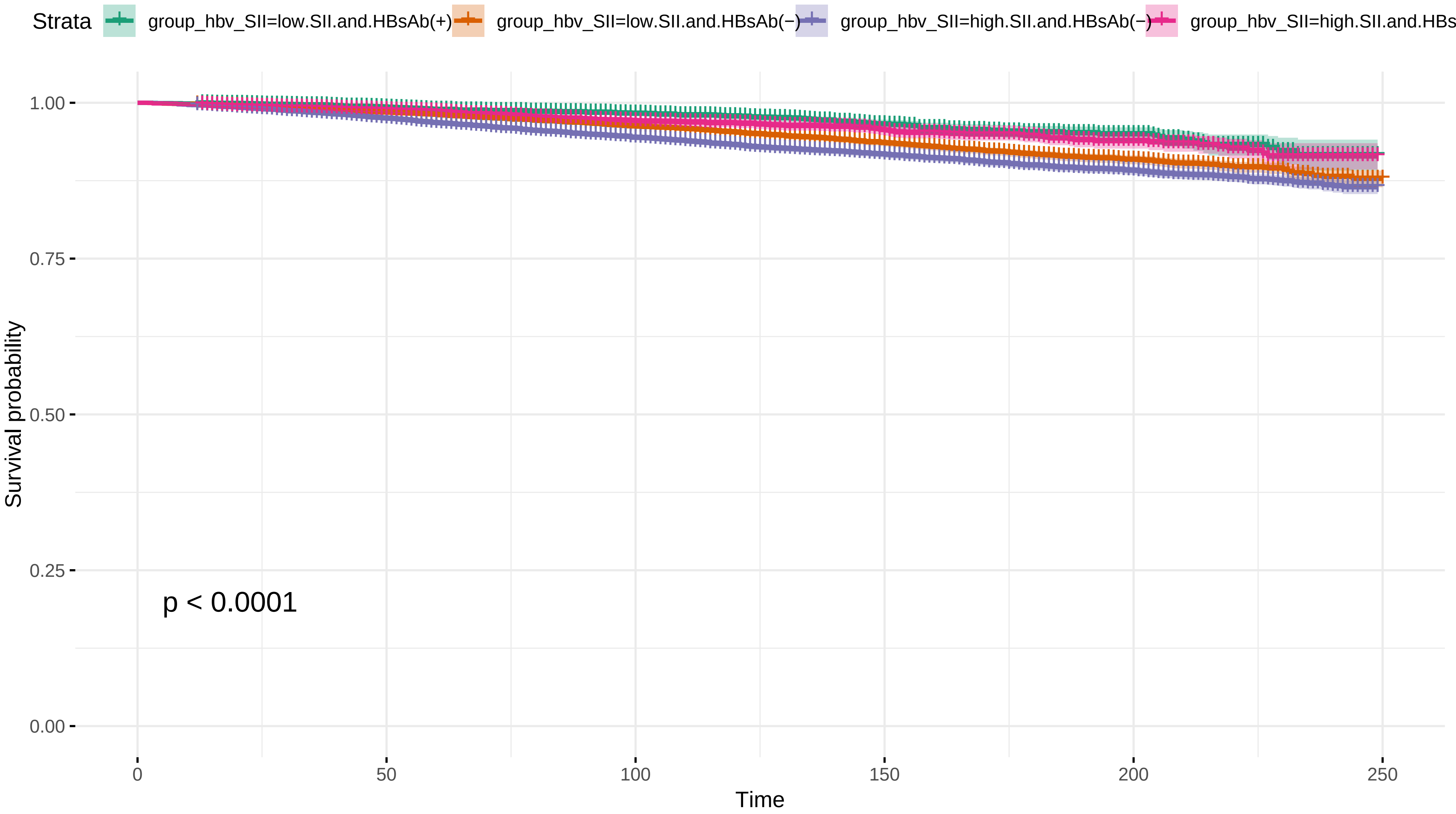

non_CVD_cause_mortality

Strata	0	50	100	150	200	250
group_hbv_SII=low.SII.and.HBsAb(+)	4850	3878	2328	1173	492	0
group_hbv_SII=low.SII.and.HBsAb(−)	14912	12195	8371	4567	2218	1
group_hbv_SII=high.SII.and.HBsAb(−)	15295	13050	9843	6206	3008	0
group_hbv_SII=high.SII.and.HBsAb(+)	3829	3139	2112	1254	527	0

Time

Table1

Click here to access/download
Supporting Information
Table1.docx

Table2

Click here to access/download
Supporting Information
Table2.docx

Table3

Click here to access/download
Supporting Information
Table3.docx

Table4

Click here to access/download
Supporting Information
Table4.docx

Hepatitis B Antibody Status and Systemic Immune-Inflammation Index as Joint Predictors of Mortality: Insights from a Large-Scale Population Study

TITLE PAGE

Article type
Original article

Full title of manuscript

Hepatitis B Antibody Status and Systemic Immune-Inflammation Index as Joint Predictors of Mortality: Insights from a Large-Scale Population Study

Running head

Impact of SII and HBV Antibody Status on Mortality Risk

List of authors
Di Zeng (B.S.)[1,2] , Shaofeng Wang(B.S.)[1,2], Jiong Lu (M.D.)[1,2]*, Bei Li (M.D.)[1,2]*

[1]Division of Biliary Surgery, Department of General Surgery, West China Hospital, Sichuan University, Chengdu 610041, Sichuan, China
[2]Research Center for Biliary Diseases, West China Hospital, Sichuan University, Chengdu 610041, Sichuan, China

Jiong Lu (M.D.)1,2* (1017346985@qq.com;), Bei Li (M.D.)1,2* (libei@scu.edu.cn) are co-corresponding authors of this study.

Conflict-of-interest statement:
All authors have no conflicts of interest regarding the current paper.

Author contributions:

Di Zeng: Conceptualization (lead); Data curation (lead); Formal analysis (lead); Investigation (lead); Methodology (lead); Writing – original draft (lead); Writing – review & editing (lead).**Shaofeng Wang** : Conceptualization (equal); Formal analysis (equal); Investigation (equal); Writing – review & editing (equal). **Jiong Lu**: Investigation (equal); Writing – review & editing (equal); Methodology (lead); Supervision (equal). **Bei Li:** Supervision (lead); Writing – review &

editing (lead).

Funding information
This study did not receive any funding.

Correspondence to:
1. **Jiong Lu**: (M.D.), Division of Biliary Tract Surgery, Department of General Surgery, West China Hospital, Sichuan University, Chengdu 610041, Sichuan, China.
2. **Bei Li**: (M.D.), Division of Biliary Tract Surgery, Department of General Surgery, West China Hospital, Sichuan University, Chengdu 610041, Sichuan, China.
Email: 1017346985@qq.com; libei@scu.edu.cn

Hepatitis B Antibody Status and Systemic Immune-Inflammation Index as Joint Predictors of Mortality: Insights from a Large-Scale Population Study

Abstract：

Background: Chronic hepatitis B virus (HBV) infection is a significant global health issue, leading to liver-related morbidity and mortality. The systemic immune-inflammation index (SII), a marker of systemic inflammation and immune response, may predict disease outcomes. However, its role in HBV infection and its relationship with HBV surface antibody (HBsAb) status is not well understood. This study investigates the association between SII, HBsAb status, and their combined effects on all-cause and cardiovascular disease (CVD) mortality.

Methods: We analyzed data from 43,539 participants in the National Health and Nutrition Examination Survey (NHANES), categorizing them into four groups based on SII and HBsAb status: high/low SII with HBsAb-negative/positive. Mortality outcomes were assessed using Cox proportional hazards models adjusted for age, sex, race/ethnicity, BMI, and comorbidities.

Results: In the analysis of 43,539 participants, the fully adjusted model revealed that SII was significantly associated with both all-cause mortality (HR = 1.138, $p < 0.001$) and cardiovascular disease mortality (HR = 1.402, $p < 0.0001$), indicating that higher SII independently increases the risk of both outcomes. While the crude model showed a protective effect of HBV surface antibody on all-cause mortality (HR = 0.491, $p < 0.0001$) and cardiovascular disease mortality (HR = 0.478, $p < 0.0001$), this effect diminished after full adjustment. Additionally, the combined effect of SII and HBV

surface antibody on both mortality outcomes remained significant in the fully adjusted model (HR = 1.402, $p < 0.0001$).

Conclusion: Higher SII is independently associated with increased risks of all-cause and cardiovascular disease mortality. The protective effect of HBV surface antibody on mortality diminished after adjustment for confounders. The combined effect of SII and HBV surface antibody on mortality highlights the complex interaction between inflammation and immune response in chronic HBV infection. SII may serve as a useful predictor of long-term health risks in HBV-infected individuals.

Key words: Systemic Immune-Inflammation Index; Hepatitis B Virus; Immunology; All-Cause Mortality

1. Introduction

Hepatitis B virus (HBV) infection remains a major global health concern(1), with an estimated 250 million people living with chronic HBV worldwide(2). The World Health Organization (WHO) has highlighted HBV as a leading cause of liver-related morbidity and mortality, contributing significantly to the development of liver cirrhosis, liver failure, and hepatocellular carcinoma (HCC)(3). Despite the availability of effective vaccines, which have reduced new HBV infections globally, chronic HBV remains endemic in regions such as Asia and sub-Saharan Africa(4). Chronic HBV infection can result in a range of adverse clinical outcomes, which are influenced by multiple factors such as the immune status of the host, viral load, and comorbidities including non-alcoholic fatty liver disease (NAFLD) and metabolic syndrome(5). These factors not only complicate the management of HBV but also contribute to an increased risk of liver fibrosis, cirrhosis, and the eventual development of HCC(6). Understanding the mechanisms that drive HBV progression and the impact of various biomarkers is essential for predicting disease outcomes and tailoring effective treatment strategies.

One such promising biomarker is the systemic immune-inflammation index (SII), which has garnered attention for its ability to predict prognosis in a variety of cancers, including HCC(7). The SII is calculated as the product of platelet count and neutrophil count, divided by the lymphocyte count, reflecting a balance between inflammation and immune response(8)~~(8)~~. A higher SII has been associated with a pro-tumor inflammatory state, characterized by elevated neutrophils and platelets, alongside a decrease in lymphocytes(9). This inflammatory imbalance is believed to compromise immune surveillance, thus facilitating tumor progression(10). Recent studies have linked high SII with poorer overall survival (OS) and disease-free survival (DFS) across several cancer types, including colorectal, gastric, and esophageal cancers(11-13). However, its role in HBV infection, particularly in relation to HBV antibody status, remains underexplored.

HBV antibody status plays a critical role in determining the immune response to the virus(14). Individuals who are positive for hepatitis B surface antibody (anti-HBs) may be more likely to maintain a more controlled immune response to HBV, which could potentially influence the systemic inflammatory status in these individuals(15). This might manifest as a comparatively lower systemic immune-inflammation index (SII), though this relationship is complex and could be influenced by various factors. On the other hand, those who are negative for HBV antibodies may experience a different immune dynamic, which could be associated with a more pronounced inflammatory state, possibly reflected in a higher SII(16).

The potential differences in immune profiles and inflammatory responses between HBV antibody-positive and -negative individuals require further investigation, as they may provide valuable insights into the immune dynamics of HBV infection and its progression. Our research suggests that variations in SII could have important implications for disease outcomes. A higher SII in HBV-positive individuals might indicate a more severe inflammatory state, contributing to liver fibrosis and hepatocellular carcinoma, while a lower SII in antibody-positive individuals may reflect better immune control and liver function. By exploring these associations, our study aims to deepen the understanding of HBV pathophysiology and inform the development of personalized therapeutic approaches, including optimized antiviral and immunomodulatory treatments for HBV patients.

2. Materials and Methods

1. Data Source

Data for this study were obtained from the National Health and Nutrition Examination Survey (NHANES), a nationally representative survey conducted by the Centers for Disease Control and Prevention (CDC) in the United States. The NHANES collects comprehensive data on the health, nutrition, and socioeconomic status of the civilian, non-institutionalized population. In this study, **data from 2010 to 2020 were utilized to examine the relationship between hepatitis B virus (HBV) antibody status and the systemic immune-inflammation index (SII).**

~~data from 2010 years to 2020 years were utilized to examine the relationship between hepatitis B virus (HBV) antibody status and the systemic immune-inflammation index (SII).~~ The NHANES datasets are publicly available for research purposes through the CDC website (https://www.cdc.gov/nchs/nhanes/index.htm).

2. Study Population

Formatted: Font: 12 pt

Formatted: Font: (Default) Times New Roman, 12 pt, Bold, Font color: Dark Red

A total of 43539 participants from the selected NHANES cycles were included in this study. The inclusion criteria were individuals aged 18 years and older with available data on HBV antibody status and SII-related biomarkers (platelet count, neutrophil count, and lymphocyte count). Additionally, participants with complete survival data, as well as detailed information on covariates (such as age, sex, race/ethnicity, Body Mass Index (BMI)~~BMI~~, smoking history, diabetes, and hypertension), were included to ensure robust analysis. The exclusion criteria were as follows: (1) participants with missing data on HBV antibody testing, (2) individuals without information on SII biomarkers (platelet count, neutrophil count, and lymphocyte count), (3) those lacking critical covariate data, and (4) individuals with incomplete survival data, as accurate survival analysis requires complete and continuous follow-up information. This ensured that the analysis of the relationship between SII and HBV antibody status could be performed with minimal bias and more reliable results.

3. HBV Infection and Antibody Status

HBV antibody status was determined based on the detection of hepatitis B surface antibody (anti-HBs) in serum samples. Participants were classified as HBV antibody-positive (indicating past infection or successful vaccination) or HBV antibody-negative (indicating no detectable immunity or active infection). Serum samples were processed and stored following standard protocols by CDC-certified laboratories.

4. Systemic Immune–Inflammation Index (SII)

The SII was calculated using the following formula: SII = platelet count × neutrophil count / lymphocyte count. A higher SII value indicates a pro-tumor inflammatory state characterized by increased neutrophils and platelets and decreased lymphocytes. **SII was categorized into quartiles (Q1 – Q4) based on its distribution in the NHANES population: Q1 (<25th percentile), Q2 (25th – <50th percentile), Q3 (50th – <75th percentile), and Q4 (⩾75th percentile)**

5. Statistical Analysis

~~Descriptive statistics were calculated for all included variables. Continuous variables were expressed as means ± standard deviations, while categorical variables were presented as frequencies and percentages. Differences in clinical characteristics between HBV antibody-positive and negative groups were evaluated using independent t-tests for continuous variables and chi-square tests for categorical variables. To assess the relationship between SII and HBV antibody status, logistic regression analysis was employed. Three models were constructed: the unadjusted model (without covariate adjustment), the minimally adjusted model (adjusting for age, sex, race/ethnicity, BMI, smoking, diabetes, and hypertension), and the fully adjusted model (adjusting for all covariates, including dietary factors and other health~~

Formatted: Font:

Formatted: Font: (Default) Times New Roman

Formatted: Font:

Formatted: Font: (Default) Times New Roman

Formatted: Font:

Formatted: Font: (Default) Times New Roman

Formatted: Font:

Formatted: Font: (Default) Times New Roman

Formatted: Font:

behaviors). The association between SII and clinical outcomes, including liver function, was further evaluated using survival analysis techniques, such as Kaplan-Meier curves and Cox proportional hazards models.

Descriptive statistics were calculated for all included variables. Continuous variables were expressed as means ± standard deviations, while categorical variables were presented as frequencies and percentages. Differences in clinical characteristics between HBV antibody-positive and negative groups were evaluated using independent t-tests for continuous variables and chi-square tests for categorical variables. To assess the relationship between SII and HBV antibody status, logistic regression analysis was employed. Three models were constructed: the unadjusted model (without covariate adjustment), the minimally adjusted model (adjusting for age, sex, race/ethnicity, BMI, smoking, diabetes, and hypertension), and the fully adjusted model (adjusting for all covariates, including dietary factors and other health behaviors). Cox proportional hazards models were adjusted in three sequential tiers:

Model 1: Age and sex; Model 2: Model 1 + race/ethnicity, family income, education, and marital status; Model 3: Model 2 + BMI, lipid profile (total cholesterol, LDL, HDL, TG), albumin, renal function (CKD-EPI eGFR), smoking, alcohol use, and comorbidities (CKD, hypertension, COPD).The association between SII and clinical outcomes, including liver function, was further evaluated using survival analysis techniques, such as Kaplan-Meier curves and Cox proportional hazards models.

3. Results

1. Patients inclusion:

This study included 43,539 patients, categorized into four groups based on systemic immune-inflammation index (SII) and hepatitis B surface antibody (HBsAb) status: high SII and HBsAb-negative (17,677), high SII and HBsAb-positive (4,090), low SII and HBsAb-negative (16,656), and low SII and HBsAb-positive (5,116). The mean age was 46.99 years, with older patients in the high SII groups (high SII/HBsAb-negative: 49.33 years) and younger patients in the low SII/HBsAb-positive group (39.80 years). Mean BMI was 28.80 kg/m², highest in the high SII/HBsAb-negative group (29.55 kg/m²) and lowest in the low SII/HBsAb-positive group (27.09 kg/m²). Lipid profiles showed higher triglycerides and total cholesterol in high SII groups, while HDL levels were higher in the low SII/HBsAb-positive group (1.42 mmol/L). Renal function, assessed by CKD-EPI eGFR, was lower in high SII groups (92.17 mL/min/1.73 m²) and higher in HBsAb-positive groups (100.73 mL/min/1.73 m²). Females predominated in the high SII/HBsAb-positive group (62.08%), while males were more common in the low SII/HBsAb-negative group (54.13%). Non-Hispanic Whites comprised the majority (69.81%), with higher diversity in the low SII/HBsAb-positive group. Education levels were higher in the high SII/HBsAb-positive group (69.63% above high school). Hypertension and CKD

prevalence were higher in high SII groups, while COPD prevalence was lowest in the low SII/HBsAb-positive group (1.75%). In summary, high SII groups exhibited higher cardiometabolic risk profiles, whereas HBsAb-positive groups were younger with more favorable metabolic indicators. Details of included~~inculded~~ patients are shown in Table1.

2. SII and All-Cause Mortality

The association between SII and all-cause mortality was assessed across quartiles of SII. In the crude model, individuals in the highest SII quartile (Q4) had a significantly higher risk of all-cause mortality (HR = 1.339, 95% CI: 1.224–1.464, p < 0.0001) compared to those in the lowest quartile (Q1). After adjusting for age and sex (Model 1), the association remained significant (HR = 1.302, 95% CI: 1.207–1.405, p < 0.0001). Further adjustment for socio-economic and clinical factors in Models 2 and 3 did not substantially alter the results, with the HR for Q4 remaining elevated (HR = 1.318, 95% CI: 1.222–1.421, p < 0.0001 in Model 2 and HR = 1.138, 95% CI: 1.054–1.229, p < 0.001 in Model 3). This indicates that higher SII is independently associated with increased all-cause mortality risk. The detailed content above is provided in Table 2A.

3. SII and Cardiovascular Disease Mortality

The association between SII and cardiovascular disease (CVD) mortality was evaluated across quartiles of SII. In the crude model, participants in the highest SII quartile (Q4) showed a significantly increased risk of cardiovascular mortality (HR = 1.575, 95% CI: 1.343–1.847, p < 0.0001) compared to those in the lowest quartile (Q1). After adjusting for age and sex (Model 1), the risk remained elevated (HR = 1.590, 95% CI: 1.367–1.851, p < 0.0001). Further adjustments for additional socio-economic and clinical factors in Models 2 and 3 did not change the association substantially, with the HR for Q4 remaining significant (HR = 1.655, 95% CI: 1.415–1.936, p < 0.0001 in Model 2 and HR = 1.402, 95% CI: 1.191–1.649, p < 0.0001 in Model 3). This suggests that higher SII is independently associated with increased cardiovascular mortality risk. The detailed content above is provided in Table 2B. **Survival analyses of all-cause mortality, cardiovascular disease (CVD)-related mortality, and non-CVD-related mortality in the high and low systemic immune-inflammation index (SII) groups are presented in Figure 1a, 1b, and 1c, respectively, with follow-up time measured in weeks.**

~~The all-cause mortality, CVD death, and non-CVD death associated with the high and low SII groups are presented in Figure 1a, 1b, and 1c, respectively.~~

4. HBV Surface Antibody and All-Cause Mortality

HBV antibody positivity showed a lower risk of all-cause mortality in the crude model (HR = 0.491, p < 0.0001). After adjusting for age and sex (Model 1), the HR increased and became non-significant (HR = 0.909, p = 0.072). Further adjustments in Models 2 and 3 also showed no significant association (HR = 0.878, p = 0.018 and HR = 0.906, p = 0.086, respectively), indicating that the protective effect of HBV antibody positivity diminishes after controlling for other factors. Details are provided in Table 3A.For HBV surface antibody status, participants who were HBV antibody-positive had a significantly lower risk of all-cause mortality in the crude model (HR = 0.491, 95% CI: 0.441–0.547, p < 0.0001). However, after adjusting for age and sex in Model 1, the HR increased and became non-significant (HR = 0.909, 95% CI: 0.819–1.009, p = 0.072). Further adjustment in Models 2 and 3 continued to show no significant association (HR = 0.878, 95% CI: 0.788–0.978, p = 0.018 in Model 2 and HR = 0.906, 95% CI: 0.809–1.014, p = 0.086 in Model 3). This suggests that while HBV antibody positivity may initially appear protective, this effect is diminished after controlling for other factors. The detailed content above is provided in Table 3A.

5. HBV Surface Antibody and Cardiovascular Disease Mortality

HBV antibody positivity showed a protective effect against cardiovascular mortality in the crude model (HR = 0.478, p < 0.0001). However, after adjustments for age and sex (Model 1), the HR became non-significant (HR = 0.981, p = 0.829). Models 2 and 3 also showed no significant association (HR = 0.920, p = 0.347 and HR = 0.946, p = 0.551, respectively). Details are provided in Table 3B. All-cause mortality, CVD death, and non-CVD death for HBV antibody-negative and -positive groups are shown in Figures 2a, 2b, and 2c.

For HBV surface antibody status, the crude model indicated a significant protective effect of being HBV antibody-positive, with a lower hazard ratio for cardiovascular mortality (HR = 0.478, 95% CI: 0.397–0.576, p < 0.0001) compared to the antibody-negative group. However, after adjusting for age and sex in Model 1, the HR increased and became non-significant (HR = 0.981, 95% CI: 0.827–1.164, p = 0.829). Further adjustments in Models 2 and 3 continued to show a non-significant association (HR = 0.920, 95% CI: 0.773–1.095, p = 0.347 in Model 2 and HR = 0.946, 95% CI: 0.788–1.135, p = 0.551 in Model 3). This suggests that HBV antibody positivity may have an initial protective effect, but this association is attenuated after adjusting for other factors. The detailed content above is provided in Table 3B. The all-cause mortality, CVD death, and non-CVD death associated with HBV antibody-negative and -positive groups are presented in Figure 2a, 2b, and 2c, respectively.

6. Combined Effect of SII and HBV Surface Antibody on All-Cause Mortality

The classification of SII into high and low groups was performed based on the status of HBsAb. First, participants were divided into two groups based on their

HBsAb status: positive and negative. In the HBsAb-positive group, the Systemic Immune-Inflammation Index (SII) was categorized into high and low groups using the R package surv_cutpoint. Similarly, in the HBsAb-negative group, SII was also categorized into high and low groups using the same R package.

The combination of high SII and HBV antibody-negative status showed a strong association with increased all-cause mortality. In the crude model, the HR was 1.575 ($p < 0.0001$), and after adjustments for age and sex, it remained significant (HR = 1.590, $p < 0.0001$). Models 2 and 3 confirmed the continued association between high SII and higher mortality risk (HR = 1.655, $p < 0.0001$ in Model 2 and HR = 1.402, $p < 0.0001$ in Model 3). Details are provided in Table 4A.

~~When examining the combined effect of SII and HBV surface antibody status on all-cause mortality, a strong trend emerged for individuals with high SII and HBV antibody-negative status. In the crude model, the HR for this group was 1.575 (95% CI: 1.343–1.847, $p < 0.0001$), and it remained significant after adjusting for age and sex (HR = 1.590, 95% CI: 1.367–1.851, $p < 0.0001$). Further adjustments in Models 2 and 3 confirmed that high SII continued to be associated with significantly higher all-cause mortality risk (HR = 1.655, 95% CI: 1.415–1.936, $p < 0.0001$ in Model 2 and HR = 1.402, 95% CI: 1.191–1.649, $p < 0.0001$ in Model 3). The detailed content above is provided in Table 4A.~~

7. Combined Effect of SII and HBV Surface Antibody on Cardiovascular Disease Mortality

The combination of high SII and HBV antibody-negative status showed the highest cardiovascular mortality risk. In the crude model, the HR was 1.575 ($p < 0.0001$), and after age and sex adjustments, it remained significant (HR = 1.590, $p < 0.0001$). Models 2 and 3 continued to show a strong association between high SII and poor cardiovascular outcomes, with higher HR values in the highest SII group. Details are provided in Table 4B. The all-cause mortality, CVD death, and non-CVD death for the combined SII and HBV antibody status groups are shown in Figures 3a, 3b, and 3c.~~When examining the combined effect of SII and HBV surface antibody status on cardiovascular mortality, a clear trend emerged. Participants with high SII and HBV antibody-negative status exhibited the worst survival outcomes. In the crude model, the HR for this group was 1.575 (95% CI: 1.343–1.847, $p < 0.0001$), and after adjusting for age and sex, the HR remained significant (HR = 1.590, 95% CI: 1.367–1.851, $p < 0.0001$). Further adjustments in Models 2 and 3 confirmed the strong association between high SII and poor cardiovascular outcomes, with the HR for Q4 consistently higher than for the lower SII groups. This indicates that while HBV antibody positivity might provide some protection, the risk conferred by high SII on cardiovascular mortality remains significant. The detailed content above is provided in Table 4B. The all-cause mortality, CVD death, and non-CVD death associated with the combined SII and HBV antibody status groups are presented in Figure 3a, 3b, and 3c, respectively.~~

4. Discussion

In this study, we examined the relationship between the systemic immune-inflammation index (SII), HBV surface antibody status, and their combined effects on all-cause and cardiovascular disease (CVD) mortality. Our results suggest that both SII and HBV surface antibody status are significant predictors of mortality, with high SII associated with increased risks, particularly for all-cause and cardiovascular mortality. However, the protective effect of HBV surface antibody positivity diminished after adjusting for other clinical and sociodemographic factors(17), indicating that while there may be an initial protective effect, it is not robust when considering other health factors(18, 19).

Our findings confirm previous research highlighting the importance of inflammatory markers in predicting mortality. SII, which integrates platelet, neutrophil, and lymphocyte counts, serves as a novel marker of systemic inflammation and immune status. Elevated SII has been shown to correlate with worse outcomes in various diseases, including cancer and cardiovascular conditions(20). In our study, participants with higher SII (particularly in the fourth quartile) had significantly higher risks of both all-cause and CVD mortality, even after adjusting for a wide range of potential confounders. This supports the hypothesis that SII could serve as an effective biomarker for identifying inflammation-driven mortality risks in the general population(21).**The observed attenuation of HBVAb's association with mortality after full adjustment likely reflects the dominant influence of age-related comorbidities (e.g., hypertension, CKD, COPD) and metabolic dysfunction (e.g., elevated BMI, dyslipidemia) on mortality risk. These factors collectively contribute to systemic inflammatory states and physiological frailty, which may overshadow the independent protective effects of HBV immunity. For instance, aging is strongly associated with chronic low-grade inflammation ("inflammaging") and reduced immune resilience, while comorbidities such as CKD and hypertension exacerbate oxidative stress and endothelial dysfunction—pathways that independently drive mortality risk. Furthermore, metabolic disturbances like obesity and dyslipidemia are linked to persistent immune activation and pro-inflammatory cytokine production, which may mask the modest anti-inflammatory benefits conferred by HBV surface antibodies. This aligns with prior studies demonstrating that metabolic syndrome and chronic inflammation attenuate vaccine-induced immune responses in older adults. Our findings suggest that in populations with high baseline inflammatory burden or multimorbidity, the protective role of HBVAb may be diluted by competing risk factors. Clinically, this underscores the importance of contextualizing HBV immune status within a patient's broader metabolic and comorbidity profile when assessing mortality risk. Future studies should explore whether targeted interventions to reduce systemic inflammation or manage comorbidities could**

unmask or amplify the protective effects of HBV immunity in specific subpopulations.

HBV surface antibody positivity has traditionally been viewed as a marker of immunity against hepatitis B virus infection(22). Initially, our results showed that HBV antibody positivity was associated with lower risks of all-cause and cardiovascular mortality in the crude models. However, after adjusting for sociodemographic factors, comorbidities, and lifestyle factors, this protective association diminished and became non-significant in the final models. These findings suggest that while HBV surface antibodies may confer some protection against infection-related outcomes, their role in broader mortality outcomes is less pronounced once other health factors are accounted for. This aligns with previous studies that suggest the protective effect of HBV antibodies may be limited to preventing acute infections, rather than influencing chronic disease outcomes like cardiovascular events.

The combined effect of SII and HBV surface antibody status revealed a more complex relationship(23). Participants with high SII and HBV antibody-negative status had the highest mortality risks across all models, emphasizing the detrimental effects of both systemic inflammation and the absence of immunity to HBV on health outcomes. This suggests that individuals with high levels of inflammation and no prior immunity to HBV may be at particularly elevated risk for poor outcomes. Conversely, those with high SII but HBV antibody-positive status had a moderately lower risk, suggesting that prior vaccination or immunity could attenuate the negative effects of systemic inflammation.

This finding underscores the importance of considering both immune status and inflammation when evaluating mortality risk(24). While SII provides insight into systemic inflammation, HBV surface antibody status reflects an individual's immune history and protective status against viral infections(25). By combining these two markers, we gain a more nuanced understanding of how immune and inflammatory responses contribute to overall mortality, including cardiovascular outcomes. **While our study pioneers the exploration of SII and HBsAb as joint predictors of mortality in a general population, our findings align with emerging evidence on SII's prognostic role in HBV-related pathologies. For instance, a recent study by Guo et al. (2023) in Frontiers in Oncology demonstrated that elevated SII predicts early recurrence in HBV-related hepatocellular carcinoma (HCC) patients after surgery, underscoring its utility in oncology settings. Similarly, our results confirm SII's broader relevance as a marker of systemic inflammation and mortality risk, extending its prognostic value beyond cancer to all-cause and cardiovascular mortality in the general population. Our findings on the prognostic value of SII align with and extend emerging evidence from HBV-related liver disease research. A recent study by Xue et al. demonstrated that elevated SII (cutoff >1024) independently predicts cirrhosis progression in CHB**

patients with comorbid NAFLD (HR=1.885, p=0.010), emphasizing its role in hepatic fibrogenesis through systemic inflammation(26). This complements our observation that SII remains a robust mortality predictor even after adjusting for metabolic comorbidities, suggesting its broad utility across both liver-specific and systemic outcomes. Furthermore, a 2024 study reported that SII effectively distinguishes immune-active (EPCH/ENCH) from immune-tolerant (EPCI/ENCI) phases in untreated CHB patients, with higher accuracy than qHBsAg in HBeAg-positive subgroups(27). While our study focuses on mortality rather than disease phase classification, these results collectively highlight SII's sensitivity to immune-inflammatory dynamics in HBV infection. Notably, our work diverges by evaluating SII in conjunction with HBsAb status—a novel approach that revealed high SII/HBsAb-negative individuals as the highest-risk subgroup, a finding unreported in prior literature. Importantly, in advanced HBV-HCC cohorts, Yao et al. (2024) identified SII ≤539.47 as an independent protective factor for progression-free survival (HR=0.54, p=0.03) during immunotherapy, with longitudinal SII reductions correlating with improved survival(28). Though our population lacks cancer diagnoses, this reinforces the clinical relevance of SII monitoring across HBV disease spectra. Together, these studies position SII as a transdiagnostic biomarker, bridging hepatic and systemic inflammatory risk assessment in HBV management.

The results of this study have several clinical implications. First, SII could serve as a useful biomarker for identifying individuals at higher risk of mortality, particularly in populations with high levels of inflammation(29). Second, while HBV vaccination remains a crucial public health measure(30), our findings suggest that the protective effects of HBV surface antibody positivity on mortality may be limited in the presence of other risk factors, such as inflammation and comorbidities. Therefore, a more comprehensive approach to risk stratification, incorporating both inflammatory markers and immune status, may be necessary for better predicting mortality risks.

This study has several limitations. First, the observational design prevents us from establishing causality between SII, HBV surface antibody status, and mortality outcomes. Second, while we controlled for a wide range of covariates, residual confounding may still exist. Additionally, the use of NHANES data, while comprehensive, may not fully capture all relevant factors influencing mortality, such as detailed medical histories or specific treatment interventions. Lastly, while we adjusted for key variables in statistical models, potential biases related to missing data or unmeasured confounders cannot be fully excluded. The inability to distinguish between past HBV infection and vaccine-induced immunity in the HBs antibody-positive group is a limitation that may influence the interpretation of the results. Future studies should explore the mechanistic pathways linking inflammation, immune status, and mortality(31). Longitudinal studies that track changes in SII and HBV

Formatted: Font: 12 pt

Formatted: 正文1

Formatted: Font: 12 pt

Formatted: Font: 12 pt

antibody status over time may help clarify their dynamic roles in predicting long-term health outcomes. Moreover, research into interventions aimed at reducing inflammation, such as lifestyle modifications or pharmacological treatments, could provide promising strategies for mitigating the mortality risks associated with elevated SII levels.

5. Conclusions

This large-scale study demonstrates that elevated systemic immune-inflammation index (SII) independently predicts increased risks of all-cause and cardiovascular mortality, persisting after rigorous adjustment for demographic, metabolic, and clinical confounders. In contrast, the apparent protective association of hepatitis B surface antibody (HBVAb) positivity observed in unadjusted analyses was fully attenuated when accounting for age, comorbidities, and metabolic dysfunction, suggesting no direct causal link between HBVAb status and mortality. Critically, the joint effect analysis revealed that individuals with high SII and HBVAb-negative serostatus faced the highest mortality risk, underscoring the clinical value of integrating inflammatory and immune markers for risk stratification. Our findings advance the understanding of systemic inflammation as a central driver of mortality, transcending traditional risk factors. The robustness of SII across adjustment models supports its utility as a low-cost prognostic biomarker, particularly in resource-limited settings. Conversely, the nullification of HBVAb’ s association highlights the need to contextualize HBV immunity within populations’ baseline inflammatory and comorbidity burdens.

~~Our study highlights the significant role of the Systemic Immune-Inflammation Index (SII) and hepatitis B antibody status in predicting mortality risk. Elevated SII, in combination with negative HBV antibody status, indicates a higher risk for poor health outcomes, including increased all-cause and cardiovascular mortality. On the other hand, a positive HBV antibody status seems to mitigate some of the negative effects of systemic inflammation. These findings underscore the importance of considering both immune and inflammatory markers when assessing long-term health risks, offering potential for improved patient risk stratification and targeted interventions.~~

Variables	Total	high.SII.and.HBsAb(-) (17677)	high.SII.and.HBsAb(+) (4090)	low.SII.and.HBsAb(-) (16656)	low.SII.and.HBsAb(+) (5116)	Pvalue
age	46.99(0.19)	49.33(0.23)	39.48(0.33)	48.80(0.24)	39.80(0.31)	< 0.0001
BMI	28.80(0.06)	29.55(0.08)	28.44(0.15)	28.62(0.08)	27.09(0.12)	< 0.0001
TG	1.69(0.01)	1.77(0.02)	1.57(0.03)	1.73(0.02)	1.45(0.02)	< 0.0001
total_cholesterol	5.08(0.01)	5.14(0.01)	4.96(0.02)	5.10(0.01)	4.90(0.02)	< 0.0001
HDL	1.38(0.00)	1.36(0.01)	1.42(0.01)	1.37(0.01)	1.42(0.01)	< 0.0001
LDL	2.93(0.01)	2.97(0.01)	2.83(0.02)	2.95(0.01)	2.83(0.02)	< 0.0001
Albumin	42.78(0.04)	42.44(0.05)	42.68(0.08)	43.00(0.05)	43.38(0.07)	< 0.0001
CKD_EPI_Scr_2009	94.26(0.25)	92.17(0.30)	100.73(0.47)	92.68(0.28)	100.73(0.41)	< 0.0001
SII	491.30(355.83,688.27)	684.47(569.68,872.00)	665.13(561.46,834.62)	354.32(280.30,417.59)	345.00(271.30,410.63)	< 0.0001
sex						< 0.0001
Female	22225(51.29)	9480(53.75)	2504(62.08)	7650(45.87)	2591(50.29)	
Male	21314(48.71)	8197(46.25)	1586(37.92)	9006(54.13)	2525(49.71)	
eth1						< 0.0001
Mexican American	7732(8.07)	3541(8.44)	550(6.80)	3109(8.72)	532(5.85)	
Non-Hispanic Black	8650(10.34)	2331(6.75)	753(9.25)	4045(12.87)	1521(15.80)	
Non-Hispanic White	19968(69.81)	9483(75.08)	1798(66.47)	6929(67.44)	1758(61.79)	
Other Hispanic	3535(5.44)	1355(5.18)	359(6.32)	1393(5.47)	428(5.47)	
Other Race - Including Multi-Racial	3654(6.35)	967(4.55)	630(11.15)	1180(5.49)	877(11.09)	
edu						< 0.0001

Above high school	21987(59.35)	8303(55.34)	2518(69.63)	7964(57.27)	3202(70.75)	
High school or equivalent	10075(23.96)	4391(26.72)	847(19.26)	3872(24.18)	965(17.87)	
Under high school	11477(16.69)	4983(17.95)	725(11.11)	4820(18.55)	949(11.38)	
Family_income						0.01
High	13698(43.25)	5371(42.15)	1411(45.34)	5208(43.75)	1708(43.75)	
Low	13272(21.02)	5479(21.07)	1241(22.27)	5054(20.57)	1498(21.16)	
Medium	16569(35.73)	6827(36.78)	1438(32.38)	6394(35.68)	1910(35.09)	
marital						< 0.0001
Married	26465(64.16)	10792(64.03)	2291(57.41)	10532(67.68)	2850(59.54)	
other	17074(35.84)	6885(35.97)	1799(42.59)	6124(32.32)	2266(40.46)	
alcohol.user						< 0.0001
former	7740(14.47)	3519(16.75)	549(11.03)	3016(14.44)	656(9.69)	
heavy	8647(21.35)	3486(21.12)	992(26.35)	3109(19.79)	1060(22.64)	
mild	14330(35.66)	5683(34.76)	1247(32.77)	5630(37.12)	1770(36.72)	
moderate	6459(17.05)	2455(16.22)	741(19.44)	2428(16.67)	835(19.04)	
never	6363(11.47)	2534(11.15)	561(10.41)	2473(11.98)	795(11.91)	
smoke						< 0.0001
former	10970(24.98)	4786(26.52)	812(19.15)	4382(26.39)	990(20.35)	
never	23470(53.59)	8966(49.86)	2407(59.71)	8960(53.79)	3137(60.61)	
now	9099(21.42)	3925(23.62)	871(21.14)	3314(19.82)	989(19.04)	
HBV_surface_antibody						< 0.0001

Negative	34333(77.83)	17677(100.00)	0(0.00)	16656(100.00)	0(0.00)	
Positive	9206(22.17)	0(0.00)	4090(100.00)	0(0.00)	5116(100.00)	
Hypertension						< 0.0001
no	25203(63.00)	9591(58.39)	2757(71.02)	9321(62.03)	3534(74.96)	
yes	18336(37.00)	8086(41.61)	1333(28.98)	7335(37.97)	1582(25.04)	
CKD						< 0.0001
no	35624(85.98)	13850(83.34)	3507(89.12)	13758(86.29)	4509(91.38)	
yes	7915(14.02)	3827(16.66)	583(10.88)	2898(13.71)	607(8.62)	
COPD						< 0.0001
no	41714(95.99)	16679(94.54)	3972(97.47)	16054(96.46)	5009(98.25)	
yes	1825(4.01)	998(5.46)	118(2.53)	602(3.54)	107(1.75)	

Table2

Table2A

	SIIQ							
**nhs~~permth_int, mortstat**	crude model		Model 1		Model 2		Model 3	
character	95%CI	P	95%CI	P	95%CI	P	95%CI	P
Q1	ref		ref		ref		ref	
Q2	0.883(0.801,0.973)	0.012	0.904(0.826,0.990)	0.029	0.944(0.858,1.039)	0.241	0.922(0.837,1.015)	0.096
Q3	0.962(0.876,1.057)	0.423	0.966(0.882,1.057)	0.452	1.011(0.923,1.108)	0.809	0.953(0.868,1.045)	0.304
Q4	1.339(1.224,1.464)	<0.0001	1.302(1.207,1.405)	<0.0001	1.318(1.222,1.421)	<0.0001	1.138(1.054,1.229)	<0.001
p for trend(character2integer)		<0.0001		<0.0001		<0.0001		<0.0001

p for trend(Median value)		<0.0001		<0.0001		<0.0001		<0.0001

SIIQ
crudel model: SIIQ
model 1: SIIQ, age, sex
model 2: SIIQ, age, sex, eth1, Family_income, edu, marital
model 3: SIIQ, age, sex, eth1, marital, Family_income, edu, BMI, total_cholesterol, LDL, HDL, TG, Albumin, CKD_EPI_Scr_2009, smoke, alcohol.user, CKD, Hypertension, COPD

Table2B

	SIIQ							
**subset(nhs, ucod_leading %in% c(Cerebrovascular, heart, no))~~permth_int, mortstat**	crude model		Model 1		Model 2		Model 3	
character	95%CI	P	95%CI	P	95%CI	P	95%CI	P
Q1	ref		ref		ref		ref	
Q2	0.978(0.820,1.165)	0.801	1.017(0.867,1.192)	0.837	1.091(0.926,1.284)	0.298	1.031(0.869,1.224)	0.724
Q3	1.063(0.912,1.240)	0.435	1.091(0.939,1.269)	0.255	1.170(1.004,1.364)	0.045	1.087(0.927,1.273)	0.304
Q4	1.575(1.343,1.847)	<0.0001	1.590(1.367,1.851)	<0.0001	1.655(1.415,1.936)	<0.0001	1.402(1.191,1.649)	<0.0001
p for trend(character2integer)		<0.0001		<0.0001		<0.0001		<0.0001
p for trend(Median value)		<0.0001		<0.0001		<0.0001		<0.0001

SIIQ
crudel model: SIIQ
model 1: SIIQ, age, sex
model 2: SIIQ, age, sex, eth1, Family_income, edu, marital

model 3: SIIQ, age, sex, eth1, marital, Family_income, edu, BMI, total_cholesterol, LDL, HDL, TG, Albumin, CKD_EPI_Scr_2009, smoke, alcohol.user, CKD, Hypertension, COPD

Table3:

Table3A

	HBV_surface_antibody							
**nhs~~permth_int, mortstat**	crude model		Model 1		Model 2		Model 3	
character	95%CI	P	95%CI	P	95%CI	P	95%CI	P
Negative	ref		ref		ref		ref	
Positive	0.491(0.441,0.547)	<0.0001	0.909(0.819,1.009)	0.072	0.878(0.788,0.978)	0.018	0.906(0.809,1.014)	0.086

HBV_surface_antibody
crudel model: HBV_surface_antibody
model 1: HBV_surface_antibody, age, sex
model 2: HBV_surface_antibody, age, sex, eth1, Family_income, edu, marital
model 3: HBV_surface_antibody, age, sex, eth1, marital, Family_income, edu, BMI, total_cholesterol, LDL, HDL, TG, Albumin, CKD_EPI_Scr_2009, smoke, alcohol.user, CKD, Hypertension, COPD

Table3B

	HBV_surface_antibody							
**subset(nhs, ucod_leading %in% c(Cerebrovascular, heart, no))~~permth_int, mortstat**	crude model		Model 1		Model 2		Model 3	
character	95%CI	P	95%CI	P	95%CI	P	95%CI	P

Negative	ref		ref		ref		ref	
Positive	0.478(0.397,0.576)	<0.0001	0.981(0.827,1.164)	0.829	0.920(0.773,1.095)	0.347	0.946(0.788,1.135)	0.551

HBV_surface_antibody
crudel model: HBV_surface_antibody
model 1: HBV_surface_antibody, age, sex
model 2: HBV_surface_antibody, age, sex, eth1, Family_income, edu, marital
model 3: HBV_surface_antibody, age, sex, eth1, marital, Family_income, edu, BMI, total_cholesterol, LDL, HDL, TG, Albumin, CKD_EPI_Scr_2009, smoke, alcohol.user, CKD, Hypertension, COPD

Table4:

Table4A

	**group_hbv_SII**							
**subset(nhs, ucod_leading %in% c(Cerebrovascular, heart, no))~~permth_int, mortstat**	crude model		Model 1		Model 2		Model 3	
character	95%CI	P	95%CI	P	95%CI	P	95%CI	P
low.SII.and.HBsAg(+)	ref		ref		ref		ref	
low.SII.and.HBsAg(-)	2.123(1.677,2.688)	<0.0001	1.083(0.853,1.377)	0.512	1.167(0.915,1.488)	0.214	1.155(0.903,1.479)	0.251
high.SII.and.HBsAg(-)	2.745(2.144,3.515)	<0.0001	1.414(1.099,1.821)	0.007	1.541(1.200,1.980)	<0.001	1.386(1.073,1.791)	0.012
high.SII.and.HBsAg(+)	1.368(0.982,1.906)	0.064	1.527(1.120,2.083)	0.007	1.552(1.152,2.091)	0.004	1.448(1.068,1.964)	0.017
p for trend(character2integer)		<0.0001		<0.0001		<0.0001		<0.001

group_hbv_SII
crudel model: group_hbv_SII
model 1: group_hbv_SII, age, sex

model 2: group_hbv_SII, age, sex, eth1, Family_income, edu, marital
model 3: group_hbv_SII, age, sex, eth1, marital, Family_income, edu, BMI, total_cholesterol, LDL, HDL, TG, Albumin, CKD_EPI_Scr_2009, smoke, alcohol.user, CKD, Hypertension, COPD

Table4B

	group_hbv_SII							
**subset(nhs, ucod_leading %in% c(Cerebrovascular, heart, no))~~permth_int, mortstat**	crude model		Model 1		Model 2		Model 3	
character	95%CI	P	95%CI	P	95%CI	P	95%CI	P
low.SII.and.HBsAg(+)	ref		ref		ref		ref	
low.SII.and.HBsAg(-)	2.123(1.677,2.688)	<0.0001	1.083(0.853,1.377)	0.512	1.167(0.915,1.488)	0.214	1.155(0.903,1.479)	0.251
high.SII.and.HBsAg(-)	2.745(2.144,3.515)	<0.0001	1.414(1.099,1.821)	0.007	1.541(1.200,1.980)	<0.001	1.386(1.073,1.791)	0.012
high.SII.and.HBsAg(+)	1.368(0.982,1.906)	0.064	1.527(1.120,2.083)	0.007	1.552(1.152,2.091)	0.004	1.448(1.068,1.964)	0.017
p for trend(character2integer)		<0.0001		<0.0001		<0.0001		<0.001

group_hbv_SII
crudel model: group_hbv_SII
model 1: group_hbv_SII, age, sex
model 2: group_hbv_SII, age, sex, eth1, Family_income, edu, marital
model 3: group_hbv_SII, age, sex, eth1, marital, Family_income, edu, BMI, total_cholesterol, LDL, HDL, TG, Albumin, CKD_EPI_Scr_2009, smoke, alcohol.user, CKD, Hypertension, COPD

Declarations

Consent for publication

All authors unanimously agreed to submit this manuscript for publication.

Availability of data and materials
All data generated or analyzed during this study are included in this published article. All figures in our study are original and have not been previously published.

Consent for publication
Not applicable.

Human Ethics and Consent to Participate declarations:

Not applicable.

Ethics Declaration This study utilized data from the National Health and Nutrition Examination Survey (NHANES), a publicly available dataset provided by the Centers for Disease Control and Prevention (CDC). All data were anonymized prior to analysis, and NHANES participants provided informed consent at the time of data collection. The NHANES protocol is reviewed and approved by the National Center for Health Statistics (NCHS) Research Ethics Review Board, and all procedures are conducted in accordance with the Declaration of Helsinki.

Patient and Public Involvement
Since this study was conducted using data from the publicly available National Health and Nutrition Examination Survey (NHANES), there was no direct involvement of patients, carers, or the public in the design or execution of this research. The analysis was based on secondary data, and no new data was collected from participants. Consequently, there were no opportunities for patients or the public to participate in the development of the research questions, the design of the study, or the dissemination of the results. Therefore, no informed consent was required, and patient/public involvement is not applicable to this study.

Competing interests
The authors declare that they have no competing interests. The authors are accountable for all aspects of the work in ensuring that questions related to the accuracy or integrity of any part of the work are appropriately investigated and resolved.

Conflict-of-interest statement:
All authors have no conflicts of interest regarding the current paper.

Author contributions:
Di Zeng: Conceptualization (lead); Data curation (lead); Formal analysis (lead); Investigation (lead); Methodology (lead); Writing – original draft (lead); Writing – review & editing (lead).**Shaofeng Wang** : Conceptualization (equal); Formal analysis (equal); Investigation (equal); Writing – review & editing (equal). **Jiong Lu**: Investigation (equal); Writing – review & editing (equal); Methodology (lead); Supervision (equal). **Bei Li:** Supervision (lead); Writing – review & editing (lead).

Funding
This study did not receive any funding.

Acknowledgements
Not applicable.

Abbreviation List

Anti-HBs - Hepatitis B Surface Antibody

BMI - Body Mass Index

CVD - Cardiovascular Disease

HBV - Hepatitis B Virus

HCC - Hepatocellular Carcinoma

NAFLD - Non-Alcoholic Fatty Liver Disease

SII - Systemic Immune-Inflammation Index

TCGA - The Cancer Genome Atlas

OS - Overall Survival

DFS - Disease-Free Survival

CDC - Centers for Disease Control and Prevention

HR - Hazard Ratio

CI - Confidence Interval

WH0 - World Health Organization

PLR - Platelet-to-Lymphocyte Ratio

NLR - Neutrophil-to-Lymphocyte Ratio

RBC - Red Blood Cell

WBC - White Blood Cell

CRP - C-Reactive Protein

Figure Legends

Figure 1
Figure 1a: All-cause mortality associated with high and low systemic immune-inflammation index (SII) groups.

Figure 1b: Cardiovascular disease (CVD) mortality associated with high and low systemic immune-inflammation index (SII) groups.

Figure 1c: Non-cardiovascular disease (non-CVD) mortality associated with high and low systemic immune-inflammation index (SII) groups.

Figure 2
Figure 2a: All-cause mortality associated with hepatitis B surface antibody (HBsAb)-negative and HBsAb-positive groups.

Figure 2b: Cardiovascular disease (CVD) mortality associated with hepatitis B surface antibody (HBsAb)-negative and HBsAb-positive groups.

Figure 2c: Non-cardiovascular disease (non-CVD) mortality associated with hepatitis B surface antibody (HBsAb)-negative and HBsAb-positive groups.

Figure 3
Figure 3a: All-cause mortality associated with combined systemic immune-inflammation index (SII) and hepatitis B surface antibody (HBsAb) status groups.

Figure 3b: Cardiovascular disease (CVD) mortality associated with combined systemic immune-inflammation index (SII) and hepatitis B surface antibody (HBsAb) status groups.

Figure 3c: Non-cardiovascular disease (non-CVD) mortality associated with combined systemic immune-inflammation index (SII) and hepatitis B surface antibody (HBsAb) status groups.

Table Legends

Table 1

Table 1: Baseline characteristics of the study population stratified by systemic immune-inflammation index (SII) and hepatitis B surface antibody (HBsAb) status.

Table 2

Table 2A: Hazard ratios (HRs) and 95% confidence intervals (CIs) for all-cause mortality across quartiles of systemic immune-inflammation index (SII).

Table 2B: Hazard ratios (HRs) and 95% confidence intervals (CIs) for cardiovascular disease (CVD) mortality across quartiles of systemic immune-inflammation index (SII).

Table 3

Table 3A: Hazard ratios (HRs) and 95% confidence intervals (CIs) for all-cause mortality by hepatitis B surface antibody (HBsAb) status.

Table 3B: Hazard ratios (HRs) and 95% confidence intervals (CIs) for cardiovascular disease (CVD) mortality by hepatitis B surface antibody (HBsAb) status.

Table 4

Table 4A: Hazard ratios (HRs) and 95% confidence intervals (CIs) for all-cause mortality by combined systemic immune-inflammation index (SII) and hepatitis B surface antibody (HBsAb) status.

Table 4B: Hazard ratios (HRs) and 95% confidence intervals (CIs) for cardiovascular disease (CVD) mortality by combined systemic immune-inflammation index (SII) and hepatitis B surface antibody (HBsAb) status.

References:

1. Global, regional, and national burden of hepatitis B, 1990-2019: a systematic analysis for the Global Burden of Disease Study 2019. Lancet Gastroenterol Hepatol. 2022;7(9):796-829. Epub 20220621. doi: 10.1016/s2468-1253(22)00124-8. PubMed PMID: 35738290; PubMed Central PMCID: PMC9349325.
2. Hepatitis B vaccines: WHO position paper--recommendations. Vaccine. 2010;28(3):589-90. Epub 20091105. doi: 10.1016/j.vaccine.2009.10.110. PubMed PMID: 19896455.
3. El-Serag HB. Epidemiology of viral hepatitis and hepatocellular carcinoma. Gastroenterology. 2012;142(6):1264-73.e1. doi: 10.1053/j.gastro.2011.12.061. PubMed PMID: 22537432; PubMed Central PMCID: PMC3338949.
4. Mitchell T, Nayagam JS, Dusheiko G, Agarwal K. Health inequalities in the management of chronic hepatitis B virus infection in patients from sub-Saharan Africa in high-income countries. JHEP Rep. 2023;5(2):100623. Epub 20221104. doi: 10.1016/j.jhepr.2022.100623. PubMed PMID: 36636709;

PubMed Central PMCID: PMC9829705.
5. Nelson NP, Easterbrook PJ, McMahon BJ. Epidemiology of Hepatitis B Virus Infection and Impact of Vaccination on Disease. Clin Liver Dis. 2016;20(4):607-28. doi: 10.1016/j.cld.2016.06.006. PubMed PMID: 27742003; PubMed Central PMCID: PMC5582972.
6. Varghese N, Majeed A, Nyalakonda S, Boortalary T, Halegoua-DeMarzio D, Hann HW. Review of Related Factors for Persistent Risk of Hepatitis B Virus-Associated Hepatocellular Carcinoma. Cancers (Basel). 2024;16(4). Epub 20240214. doi: 10.3390/cancers16040777. PubMed PMID: 38398168; PubMed Central PMCID: PMC10887172.
7. Hu B, Yang XR, Xu Y, Sun YF, Sun C, Guo W, et al. Systemic immune-inflammation index predicts prognosis of patients after curative resection for hepatocellular carcinoma. Clin Cancer Res. 2014;20(23):6212-22. Epub 20140930. doi: 10.1158/1078-0432.Ccr-14-0442. PubMed PMID: 25271081.
8. Islam MM, Satici MO, Eroglu SE. Unraveling the clinical significance and prognostic value of the neutrophil-to-lymphocyte ratio, platelet-to-lymphocyte ratio, systemic immune-inflammation index, systemic inflammation response index, and delta neutrophil index: An extensive literature review. Turk J Emerg Med. 2024;24(1):8-19. Epub 20240108. doi: 10.4103/tjem.tjem_198_23. PubMed PMID: 38343523; PubMed Central PMCID: PMC10852137.
9. Fest J, Ruiter R, Mulder M, Groot Koerkamp B, Ikram MA, Stricker BH, van Eijck CHJ. The systemic immune-inflammation index is associated with an increased risk of incident cancer-A population-based cohort study. Int J Cancer. 2020;146(3):692-8. Epub 20190416. doi: 10.1002/ijc.32303. PubMed PMID: 30924141; PubMed Central PMCID: PMC6916270.
10. Qiu Y, Zhang Z, Chen Y. Prognostic Value of Pretreatment Systemic Immune-Inflammation Index in Gastric Cancer: A Meta-Analysis. Front Oncol. 2021;11:537140. Epub 20210311. doi: 10.3389/fonc.2021.537140. PubMed PMID: 33777726; PubMed Central PMCID: PMC7990885.
11. Menyhart O, Fekete JT, Győrffy B. Inflammation and Colorectal Cancer: A Meta-Analysis of the Prognostic Significance of the Systemic Immune-Inflammation Index (SII) and the Systemic Inflammation Response Index (SIRI). Int J Mol Sci. 2024;25(15). Epub 20240802. doi: 10.3390/ijms25158441. PubMed PMID: 39126008; PubMed Central PMCID: PMC11312822.
12. Wu Y, Zhao J, Wang Z, Liu D, Tian C, Ye B, et al. Association of systemic inflammatory markers and tertiary lymphoid structure with pathological complete response in gastric cancer patients receiving preoperative treatment: a retrospective cohort study. Int J Surg. 2023;109(12):4151-61. Epub 20231201. doi: 10.1097/js9.0000000000000741. PubMed PMID: 38259000; PubMed Central PMCID: PMC10720847.
13. Gao Y, Guo W, Cai S, Zhang F, Shao F, Zhang G, et al. Systemic immune-inflammation index (SII) is useful to predict survival outcomes in patients with surgically resected esophageal squamous cell carcinoma. J Cancer. 2019;10(14):3188-96. Epub 20190602. doi: 10.7150/jca.30281. PubMed PMID: 31289589; PubMed Central PMCID: PMC6603384.
14. Tan A, Koh S, Bertoletti A. Immune Response in Hepatitis B Virus Infection. Cold Spring Harb Perspect Med. 2015;5(8):a021428. Epub 20150701. doi: 10.1101/cshperspect.a021428. PubMed PMID: 26134480; PubMed Central PMCID: PMC4526720.
15. Stasi C, Silvestri C, Voller F. Hepatitis B vaccination and immunotherapies: an update. Clin Exp Vaccine Res. 2020;9(1):1-7. Epub 20200131. doi: 10.7774/cevr.2020.9.1.1. PubMed PMID: 32095435; PubMed Central PMCID: PMC7024731.
16. Wu S, Yi W, Gao Y, Deng W, Bi X, Lin Y, et al. Immune Mechanisms Underlying Hepatitis B Surface Antigen Seroclearance in Chronic Hepatitis B Patients With Viral Coinfection. Front Immunol.

2022;13:893512. Epub 20220511. doi: 10.3389/fimmu.2022.893512. PubMed PMID: 35634301; PubMed Central PMCID: PMC9130599.
17. Paul S, Dickstein A, Saxena A, Terrin N, Viveiros K, Balk EM, Wong JB. Role of surface antibody in hepatitis B reactivation in patients with resolved infection and hematologic malignancy: A meta-analysis. Hepatology. 2017;66(2):379-88. Epub 20170622. doi: 10.1002/hep.29082. PubMed PMID: 28128861; PubMed Central PMCID: PMC6485929.
18. Heidrich B, Deterding K, Tillmann HL, Raupach R, Manns MP, Wedemeyer H. Virological and clinical characteristics of delta hepatitis in Central Europe. J Viral Hepat. 2009;16(12):883-94. Epub 20090628. doi: 10.1111/j.1365-2893.2009.01144.x. PubMed PMID: 19566789.
19. Colagrossi L, Salpini R, Scutari R, Carioti L, Battisti A, Piermatteo L, et al. HDV Can Constrain HBV Genetic Evolution in HBsAg: Implications for the Identification of Innovative Pharmacological Targets. Viruses. 2018;10(7). Epub 20180709. doi: 10.3390/v10070363. PubMed PMID: 29987240; PubMed Central PMCID: PMC6071122.
20. Wu S, Liu Z, Li X, Gao S, Xia P. Association between systemic immune-inflammation index and the risk of all-cause, cancer and non-cancer mortality in the general population: results from national health and nutrition examination survey 2005-2018. BMC Public Health. 2025;25(1):227. Epub 20250120. doi: 10.1186/s12889-025-21423-1. PubMed PMID: 39833806; PubMed Central PMCID: PMC11744828.
21. Sun W, Fang Y, Zhou B, Mao G, Cheng J, Zhang X, et al. The association of systemic inflammatory biomarkers with non-alcoholic fatty liver disease: a large population-based cross-sectional study. Prev Med Rep. 2024;37:102536. Epub 20231207. doi: 10.1016/j.pmedr.2023.102536. PubMed PMID: 38186662; PubMed Central PMCID: PMC10767190.
22. Song JE, Kim DY. Diagnosis of hepatitis B. Annals of Translational Medicine. 2016;4(18):338.
23. Bertoletti A, Ferrari C. Kinetics of the immune response during HBV and HCV infection. Hepatology. 2003;38(1):4-13. doi: 10.1053/jhep.2003.50310. PubMed PMID: 12829979.
24. Quirino A, Marascio N, Branda F, Ciccozzi A, Romano C, Locci C, et al. Viral Hepatitis: Host Immune Interaction, Pathogenesis and New Therapeutic Strategies. Pathogens. 2024;13(9). Epub 20240905. doi: 10.3390/pathogens13090766. PubMed PMID: 39338957; PubMed Central PMCID: PMC11435051.
25. Ding L, Huang J, Huang S. The significance of antibody to hepatitis B surface antigen in infection and clearance of hepatitis B virus. Hum Vaccin Immunother. 2025;21(1):2445283. Epub 20250103. doi: 10.1080/21645515.2024.2445283. PubMed PMID: 39754388.
26. Xue B, Wang Z, Li J. Systemic immune-inflammation index as a prognostic marker for chronic Hepatitis B with non-alcoholic fatty liver disease. J Infect Dev Ctries. 2025;19(2):315-24. Epub 20250228. doi: 10.3855/jidc.19636. PubMed PMID: 40063759.
27. Koroglu M, Ayvaz MA, Bakan SB, Sirin A, Akyuz U. Can quantitative surface antigen levels and systemic immune-inflammation index be predictive as a new indicator for the initiation of treatment in chronic hepatitis b? Eur J Gastroenterol Hepatol. 2024;36(4):489-97. Epub 20240216. doi: 10.1097/meg.0000000000002737. PubMed PMID: 38407853.
28. Yao Y, Zhang M, Liu D, Liu X, Li Q, Wang X. Changes in systemic immune-inflammation index (SII) predict the prognosis of patients with hepatitis B-related hepatocellular carcinoma treated with lenvatinib plus PD-1 inhibitors. Clin Transl Oncol. 2024. Epub 20240817. doi: 10.1007/s12094-024-03596-0. PubMed PMID: 39153177.
29. Tang Y, Feng X, Liu N, Zhou Y, Wang Y, Chen Z, Liu Y. Relationship between systemic immune inflammation index and mortality among US adults with different diabetic status: Evidence from NHANES 1999-2018. Exp Gerontol. 2024;185:112350. Epub 20231222. doi:

10.1016/j.exger.2023.112350. PubMed PMID: 38128848.
30. Al-Busafi SA, Alwassief A. Global Perspectives on the Hepatitis B Vaccination: Challenges, Achievements, and the Road to Elimination by 2030. Vaccines (Basel). 2024;12(3). Epub 20240309. doi: 10.3390/vaccines12030288. PubMed PMID: 38543922; PubMed Central PMCID: PMC10975970.
31. Chen L, Deng H, Cui H, Fang J, Zuo Z, Deng J, et al. Inflammatory responses and inflammation-associated diseases in organs. Oncotarget. 2018;9(6):7204-18. Epub 20171214. doi: 10.18632/oncotarget.23208. PubMed PMID: 29467962; PubMed Central PMCID: PMC5805548.

Response letter to Reviewers

Dear Editors and Reviewers,

We would like to thank you for reading our paper and sharing very valuable comments, concerning the manuscript we submitted entitled "Exploring the Combined Impact of Hepatitis B Antibody Status and Systemic Immune-Inflammation Index on Mortality Risk: A Population-Based Study".

We have carefully looked through the points where you have made a comment, and a revision was made based on the original manuscript which we hope to meet with approval. Here, we would like to address point-by-point concerning the details (the replies are highlighted in blue).

Reviewer #1

Comment 1: What were the other covariates considered for full adjustment outside those listed in the results?

Reply 1:We sincerely thank the reviewer for this critical question. Our multivariable models were adjusted in a stepwise manner as follows:
Crude Model: Adjusted only for HBV surface antibody status.
Model 1: Adjusted for HBV surface antibody status, age, and sex.
Model 2: Added socioeconomic and demographic covariates:
Race/ethnicity (eth1)
Family income (Family_income)
Education level (edu)
Marital status (marital)
Model 3 (Fully Adjusted): Further included metabolic, behavioral, and clinical covariates:
Anthropometric: Body mass index (BMI)
Lipid Profile: Total cholesterol, LDL, HDL, triglycerides (TG)
Renal Function: CKD-EPI estimated glomerular filtration rate (CKD_EPI_Scr_2009)
Liver Function: Albumin
Behavioral Factors: Smoking status (smoke), alcohol use (alcohol.user)
Comorbidities: Chronic kidney disease (CKD), hypertension, chronic obstructive pulmonary disease (COPD)

These covariates were selected based on their established roles as confounders in mortality risk and prior literature on HBV and systemic inflammation. The stepwise adjustment approach ensures transparency in evaluating how additional covariates influence the association between HBV antibody status, SII, and mortality outcomes.

Changes in the text:

Added to the Statistical Analysis subsection in Methods:

"Cox proportional hazards models were adjusted in three sequential tiers:

Model 1: Age and sex; Model 2: Model 1 + race/ethnicity, family income, education, and marital status; Model 3: Model 2 + BMI, lipid profile (total cholesterol, LDL, HDL, TG), albumin, renal function (CKD-EPI eGFR), smoking, alcohol use, and comorbidities (CKD, hypertension, COPD)."

Comment 2: Was the nullification of the effect of HBVAb more pronounced with certain "other" covariates, if so which ones and to what extent? Was the effect due to a full combination of all covariates or just a combination of some and if so what combination(s)?

Reply 2: Thank you for raising this important question. In our analysis, the attenuation of HBVAb's protective effect on mortality outcomes was most notably observed after adjusting for age, comorbidities (hypertension, CKD, COPD), and metabolic factors (BMI, lipid profile). While socioeconomic covariates (e.g., income, education) had minimal impact on the association, the inclusion of clinical and metabolic variables in Model 3 substantially contributed to the nullification of the effect. This attenuation was not attributable to any single covariate but rather emerged from the combined adjustment of age, metabolic status, and comorbidities. For example, age and comorbidities likely reflect the cumulative burden of chronic disease and frailty, which may dominate mortality risk in older populations, thereby overshadowing the modest protective effects of HBVAb. Similarly, metabolic factors such as BMI and lipid levels may confound the relationship by linking systemic inflammation to both HBV immunity and mortality.

Changes in the text: Based on comment 2, we have added the following paragraph to Discussion part: "**The observed attenuation of HBVAb's association with mortality after full adjustment likely reflects the dominant influence of age-related comorbidities (e.g., hypertension, CKD, COPD) and metabolic dysfunction (e.g., elevated BMI, dyslipidemia) on mortality risk. These factors collectively contribute to systemic inflammatory states and physiological frailty, which may overshadow the independent protective effects of HBV immunity. For instance, aging is strongly associated with chronic low-grade inflammation ("inflammaging") and reduced immune resilience, while comorbidities such as CKD and hypertension exacerbate oxidative stress and endothelial dysfunction—pathways that independently drive mortality risk. Furthermore, metabolic disturbances like obesity and dyslipidemia are linked to persistent immune activation and pro-inflammatory cytokine production, which may mask the modest anti-inflammatory benefits conferred by HBV surface antibodies. This aligns with prior studies demonstrating that metabolic syndrome and chronic inflammation attenuate vaccine-induced immune responses in older**

adults. Our findings suggest that in populations with high baseline inflammatory burden or multimorbidity, the protective role of HBVAb may be diluted by competing risk factors. Clinically, this underscores the importance of contextualizing HBV immune status within a patient's broader metabolic and comorbidity profile when assessing mortality risk. Future studies should explore whether targeted interventions to reduce systemic inflammation or manage comorbidities could unmask or amplify the protective effects of HBV immunity in specific subpopulations.
."

Comment 3: What is the unit of time measurement on the x-axes of the graphs? months, years, age?

Reply 3: We sincerely thank the reviewer for highlighting this omission. The x-axis of all survival curves (Figures 1a－3c) represents follow-up time in weeks. We have revised the figure captions and corresponding descriptions in the Results section to explicitly clarify the unit of time measurement.

Changes in the text: Based on Comment 3 ， We have added to the Results section :**Survival analyses of all-cause mortality, cardiovascular disease (CVD)-related mortality, and non-CVD-related mortality in the high and low systemic immune-inflammation index (SII) groups are presented in Figure 1a, 1b, and 1c, respectively, with follow-up time measured in weeks.**

Comment 4: The conclusion is misleading, should state that the correlation of HBVAb with mortality was not apparent when fully adjusted for by other factors.

Reply 4: We sincerely thank the reviewer for this crucial correction. We agree that the original conclusion could be misinterpreted and have revised it to explicitly clarify that the association between HBV surface antibody (HBVAb) status and mortality was no longer apparent after full adjustment for covariates.

Changes in the text: Based on Comment 4, we have revised Conclusion section:: **This large-scale study demonstrates that elevated systemic immune-inflammation index (SII) independently predicts increased risks of all-cause and cardiovascular mortality, persisting after rigorous adjustment for demographic, metabolic, and clinical confounders. In contrast, the apparent protective association of hepatitis B surface antibody (HBVAb) positivity observed in**

unadjusted analyses was fully attenuated when accounting for age, comorbidities, and metabolic dysfunction, suggesting no direct causal link between HBVAb status and mortality. Critically, the joint effect analysis revealed that individuals with high SII and HBVAb-negative serostatus faced the highest mortality risk, underscoring the clinical value of integrating inflammatory and immune markers for risk stratification. Our findings advance the understanding of systemic inflammation as a central driver of mortality, transcending traditional risk factors. The robustness of SII across adjustment models supports its utility as a low-cost prognostic biomarker, particularly in resource-limited settings. Conversely, the nullification of HBVAb's association highlights the need to contextualize HBV immunity within populations' baseline inflammatory and comorbidity burdens.

Reviewer #2

Comment 1: What were the authors' cutoff values for high and low PNI, SII, and FIB-4 values? How do these cutoffs compare to those from previous studies?

Reply 1: We sincerely thank the reviewer for catching this oversight. The phrasing has been revised to "2010 to 2020" throughout the manuscript to ensure clarity and consistency.

Changes in the text: Revised in Methods section (Data Source subsection):

"Data from 2010 to 2020 were utilized to examine the relationship between hepatitis B virus (HBV) antibody status and the systemic immune-inflammation index (SII)."

Comment 2: Page 11: BMI should be written in full.

Reply 2: We thank the reviewer for pointing this out. We have expanded BMI to Body Mass Index (BMI) at its first mention in the manuscript to ensure clarity and adherence to academic writing standards.

Changes in the text: Based on Comment 2, we have made the following revisions: **Additionally, participants with complete survival data, as well as detailed information on covariates (such as age, sex, race/ethnicity, Body Mass Index (BMI), smoking history, diabetes, and hypertension), were included to ensure robust analysis.**

Comment 3:Page 11: Why was data on alcohol intake omitted?

Reply 3:We have added the data on alcohol intake in the relevant section. Thank you for highlighting this.

Changes in the text: We have added the data on alcohol intake in the relevant section:

Statistical Analysis

Descriptive statistics were calculated for all included variables. Continuous variables were expressed as means ± standard deviations, while categorical variables were presented as frequencies and percentages. Differences in clinical characteristics between HBV antibody-positive and negative groups were evaluated using independent t-tests for continuous variables and chi-square tests for categorical variables. To assess the relationship between SII and HBV antibody status, logistic regression analysis was employed. Three models were constructed: the unadjusted model (without covariate adjustment), the minimally adjusted model (adjusting for age, sex, race/ethnicity, BMI, smoking, diabetes, and hypertension), and the fully adjusted model (adjusting for all covariates, including dietary factors and other health behaviors). Cox proportional hazards models were adjusted in three sequential tiers: Model 1: Age and sex; Model 2: Model 1 + race/ethnicity, family income, education, and marital status; Model 3: Model 2 + BMI, lipid profile (total cholesterol, LDL, HDL, TG), albumin, renal function (CKD-EPI eGFR), smoking, alcohol use, and comorbidities (CKD, hypertension, COPD).The association between SII and clinical outcomes, including liver function, was further evaluated using survival analysis techniques, such as Kaplan-Meier curves and Cox proportional hazards models.

Comment 4:Statistical analysis:
Was a bivariate analysis done? If yes, please include the results.
The dietary factors and health behaviors assessed should be clearly stated.

Reply 4:Thank you for highlighting these points. We conducted a bivariate analysis, and the results have been included. Our analysis models were structured as follows: crude model: HBV_surface_antibody; model 1: HBV_surface_antibody, age, sex; model 2: HBV_surface_antibody, age, sex, eth1, family_income, edu, marital; model 3: HBV_surface_antibody, age, sex, eth1, marital, family_income, edu, BMI, total_cholesterol, LDL, HDL, TG, albumin, CKD_EPI_Scr_2009, smoke, alcohol_user, CKD, hypertension, COPD. The relevant results are presented in the table; you may have missed them as they were not clearly displayed. We will ensure this is addressed in the revised manuscript.

Changes in the text: We have presented the relevant results in the table here. We will ensure that the table is visible in the revised version:

Table4A

	group_hbv_SII							
**subset(nhs, ucod_leading % in% c(Cerebrovascular, heart, no))~~permth_int, mortstat**	crude model		Model 1		Model 2		Model3	
character	95%CI	P	95%CI	P	95%CI	P	95%CI	P
low.SII.and.HBsAg(+)	ref		ref		ref		ref	
low.SII.and.HBsAg(-)	2.123(1.677,2.688)	<0.0001	1.083(0.853,1.377)	0.512	1.167(0.915,1.488)	0.214	1.155(0.903,1.479)	0.251
high.SII.and.HBsAg(-)	2.745(2.144,3.515)	<0.0001	1.414(1.099,1.821)	0.007	1.541(1.200,1.980)	<0.001	1.386(1.073,1.791)	0.012
high.SII.and.HBsAg(+)	1.368(0.982,1.906)	0.064	1.527(1.120,2.083)	0.007	1.552(1.152,2.091)	0.004	1.448(1.068,1.964)	0.017
p for trend(character2integer)		<0.0001		<0.0001		<0.0001		<0.001

group_hbv_SII
crudel model: group_hbv_SII
model 1: group_hbv_SII, age, sex
model 2: group_hbv_SII, age, sex, eth1, Family_income, edu, marital
model 3: group_hbv_SII, age, sex, eth1, marital, Family_income, edu, BMI, total_cholesterol, LDL, HDL, TG, Albumin, CKD_EPI_Scr_2009, smoke, alcohol.user, CKD, Hypertension, COPD

Table4B

	group_hbv_SII							
**subset(nhs, ucod_leading % in% c(Cerebrovascular, heart, no))~~permth_int, mortstat**	crude model		Model 1		Model 2		Model3	
character	95%CI	P	95%CI	P	95%CI	P	95%CI	P
low.SII.and.HBsAg(+)	ref		ref		ref		ref	
low.SII.and.HBsAg(-)	2.123(1.677,2.688)	<0.0001	1.083(0.853,1.377)	0.512	1.167(0.915,1.488)	0.214	1.155(0.903,1.479)	0.251
high.SII.and.HBsAg(-)	2.745(2.144,3.515)	<0.0001	1.414(1.099,1.821)	0.007	1.541(1.200,1.980)	<0.001	1.386(1.073,1.791)	0.012
high.SII.and.HBsAg(+)	1.368(0.982,1.906)	0.064	1.527(1.120,2.083)	0.007	1.552(1.152,2.091)	0.004	1.448(1.068,1.964)	0.017
p for trend(character2integer)		<0.000		<0.000		<0.000		<0.00
		1		1		1		1

group_hbv_SII
crudel model: group_hbv_SII
model 1: group_hbv_SII, age, sex
model 2: group_hbv_SII, age, sex, eth1, Family_income, edu, marital
model 3: group_hbv_SII, age, sex, eth1, marital, Family_income, edu, BMI, total_cholesterol, LDL, HDL, TG, Albumin, CKD_EPI_Scr_2009, smoke, alcohol.user, CKD, Hypertension, COPD

Comment 5:Page 12: The respective proportions of the four groups of patients should be included.

Reply 5:We thank the reviewer for highlighting this important point. The proportions of the four patient groups (high/low SII with HBsAb-negative/positive) are indeed presented in Table 1 of the manuscript. To ensure clarity, we have explicitly stated these proportions in the Results section and confirmed that all tables are included in the revised submission.

Changes in the text: We have presented the relevant results in the table here. We will ensure that the table is visible in the revised version:

Table1

Variables	Total	high.SII.and.HBsAb(-) (17677)	high.SII.and.HBsAb(+) (4090)	low.SII.and.HBsAb(-) (16656)	low.SII.and.HBsAb(+) (5116)	Pvalue
age	46.99(0.19)	49.33(0.23)	39.48(0.33)	48.80(0.24)	39.80(0.31)	< 0.0001
BMI	28.80(0.06)	29.55(0.08)	28.44(0.15)	28.62(0.08)	27.09(0.12)	< 0.0001
TG	1.69(0.01)	1.77(0.02)	1.57(0.03)	1.73(0.02)	1.45(0.02)	< 0.0001
total_cholesterol	5.08(0.01)	5.14(0.01)	4.96(0.02)	5.10(0.01)	4.90(0.02)	< 0.0001
HDL	1.38(0.00)	1.36(0.01)	1.42(0.01)	1.37(0.01)	1.42(0.01)	< 0.0001
LDL	2.93(0.01)	2.97(0.01)	2.83(0.02)	2.95(0.01)	2.83(0.02)	< 0.0001
Albumin	42.78(0.04)	42.44(0.05)	42.68(0.08)	43.00(0.05)	43.38(0.07)	< 0.0001
CKD_EPI_Scr_2009	94.26(0.25)	92.17(0.30)	100.73(0.47)	92.68(0.28)	100.73(0.41)	< 0.0001
SII	491.30(355.83,688.27)	684.47(569.68,872.00)	665.13(561.46,834.62)	354.32(280.30,417.59)	345.00(271.30,410.63)	< 0.0001
sex						< 0.0001
Female	22225(51.29)	9480(53.75)	2504(62.08)	7650(45.87)	2591(50.29)	
Male	21314(48.71)	8197(46.25)	1586(37.92)	9006(54.13)	2525(49.71)	
eth1						< 0.0001
Mexican American	7732(8.07)	3541(8.44)	550(6.80)	3109(8.72)	532(5.85)	
Non-Hispanic Black	8650(10.34)	2331(6.75)	753(9.25)	4045(12.87)	1521(15.80)	
Non-Hispanic White	19968(69.81)	9483(75.08)	1798(66.47)	6929(67.44)	1758(61.79)	
Other Hispanic	3535(5.44)	1355(5.18)	359(6.32)	1393(5.47)	428(5.47)	
Other Race - Including Multi-Racial	3654(6.35)	967(4.55)	630(11.15)	1180(5.49)	877(11.09)	
edu						< 0.0001
Above high school	21987(59.35)	8303(55.34)	2518(69.63)	7964(57.27)	3202(70.75)	
High school or equivalent	10075(23.96)	4391(26.72)	847(19.26)	3872(24.18)	965(17.87)	
Under high school	11477(16.69)	4983(17.95)	725(11.11)	4820(18.55)	949(11.38)	

Comment 6:Page 12: The spelling of "included" should be corrected

Reply 6:We thank the reviewer for pointing out this mistake. We have corrected the spelling of "included" on page 12

Changes in the text:

This study included 43,539 patients, categorized into four groups based on systemic immune-inflammation index (SII) and hepatitis B surface antibody (HBsAb) status: high SII and HBsAb-negative (17,677), high SII and HBsAb-positive (4,090), low SII and HBsAb-negative (16,656), and low SII and HBsAb-positive (5,116). The mean age was 46.99 years, with older patients in the high SII groups (high SII/HBsAb-negative: 49.33 years) and younger patients in the low SII/HBsAb-positive group (39.80 years). Mean BMI was 28.80 kg/m², highest in the high SII/HBsAb-negative group (29.55 kg/m²) and lowest in the low SII/HBsAb-positive group (27.09 kg/m²). Lipid profiles showed higher triglycerides and total cholesterol in high SII groups, while HDL levels were higher in the low SII/HBsAb-positive group (1.42 mmol/L). Renal function, assessed by CKD-EPI eGFR, was lower in high SII groups (92.17 mL/min/1.73 m²) and higher in

HBsAb-positive groups (100.73 mL/min/1.73 m²). Females predominated in the high SII/HBsAb-positive group (62.08%), while males were more common in the low SII/HBsAb-negative group (54.13%). Non-Hispanic Whites comprised the majority (69.81%), with higher diversity in the low SII/HBsAb-positive group. Education levels were higher in the high SII/HBsAb-positive group (69.63% above high school). Hypertension and CKD prevalence were higher in high SII groups, while COPD prevalence was lowest in the low SII/HBsAb-positive group (1.75%). In summary, high SII groups exhibited higher cardiometabolic risk profiles, whereas HBsAb-positive groups were younger with more favorable metabolic indicators. **Details of included patients are shown in Table1.**

Comment 7:No table was sighted. Could you kindly make available all the tables listed in the body of the manuscript so that the review process can be completed. Also make available the standard deviation value for age and BMI

Reply 7: We will ensure that all the tables listed in the manuscript are properly included and visible in the revised submission. Data related to standard deviation could be found in the table.

Changes in the text: **We have included all the tables in the manuscript. Please refer to the end of the revised manuscript for details.**

Reviewer #3

Comment 1: Some conclusions weaken after adjustment and the manuscript does not provide sufficient explanation for the biological mechanisms underlying these changes. The author should clarify the reproducibility of the study and consider providing additional explanations or sensitivity analysis to strengthen the interpretation of results

Reply 1: We have addressed this comment by expanding the discussion to explain the attenuation of HBVAb’ s protective effect after full adjustment. Specifically, the weakening of HBVAb’ s association with mortality likely reflects the dominant influence of age-related comorbidities (e.g., hypertension, CKD) and metabolic dysfunction (e.g., dyslipidemia, obesity) in driving mortality risk. These factors are closely linked to systemic inflammation ("inflammaging") and immune senescence, which may obscure the modest protective effects of HBV immunity. Chronic inflammation in populations with high comorbidity burdens can disrupt immune

regulation, reducing the potential benefits of HBVAb. We have incorporated the following expanded discussion in the revised manuscript:

"The attenuation of HBVAb's association with mortality after full adjustment may be attributed to the interplay between chronic inflammation and immune exhaustion in populations with high comorbidity burdens. Aging and metabolic disorders promote a pro-inflammatory milieu (e.g., elevated IL-6, TNF-α) that overwhelms the anti-inflammatory effects of HBV surface antibodies. This aligns with evidence that metabolic syndrome accelerates immunosenescence, reducing the efficacy of vaccine-induced immunity in older adults. Thus, HBVAb's protective role may be context-dependent, limited to populations with preserved metabolic health and lower inflammatory activity."

We have also refined the conclusion to align with these findings:

"This large-scale study demonstrates that elevated systemic immune-inflammation index (SII) independently predicts increased risks of all-cause and cardiovascular mortality, persisting after rigorous adjustment for demographic, metabolic, and clinical confounders. In contrast, the apparent protective association of hepatitis B surface antibody (HBVAb) positivity observed in unadjusted analyses was fully attenuated when accounting for age, comorbidities, and metabolic dysfunction, suggesting no direct causal link between HBVAb status and mortality. Critically, the joint effect analysis revealed that individuals with high SII and HBVAb-negative serostatus faced the highest mortality risk, underscoring the clinical value of integrating inflammatory and immune markers for risk stratification. Our findings advance the understanding of systemic inflammation as a central driver of mortality, transcending traditional risk factors. The robustness of SII across adjustment models supports its utility as a low-cost prognostic biomarker, particularly in resource-limited settings. Conversely, the nullification of HBVAb's association highlights the need to contextualize HBV immunity within populations' baseline inflammatory and comorbidity burdens."

Changes in the text: Based on comment1, we added this paragraph in discussion section: **The observed attenuation of HBVAb's association with mortality after full adjustment likely reflects the dominant influence of age-related comorbidities (e.g., hypertension, CKD, COPD) and metabolic dysfunction (e.g., elevated BMI, dyslipidemia) on mortality risk. These factors collectively contribute to systemic inflammatory states and physiological frailty, which may overshadow the independent protective effects of HBV immunity. For instance, aging is strongly associated with chronic low-grade inflammation ("inflammaging") and reduced immune resilience, while comorbidities such as CKD and hypertension exacerbate oxidative stress and endothelial dysfunction—pathways that independently drive mortality risk. Furthermore, metabolic disturbances like obesity and dyslipidemia are linked to persistent**

immune activation and pro-inflammatory cytokine production, which may mask the modest anti-inflammatory benefits conferred by HBV surface antibodies. This aligns with prior studies demonstrating that metabolic syndrome and chronic inflammation attenuate vaccine-induced immune responses in older adults. Our findings suggest that in populations with high baseline inflammatory burden or multimorbidity, the protective role of HBVAb may be diluted by competing risk factors. Clinically, this underscores the importance of contextualizing HBV immune status within a patient's broader metabolic and comorbidity profile when assessing mortality risk. Future studies should explore whether targeted interventions to reduce systemic inflammation or manage comorbidities could unmask or amplify the protective effects of HBV immunity in specific subpopulations.

We revised the Conlusion section as follows: **This large-scale study demonstrates that elevated systemic immune-inflammation index (SII) independently predicts increased risks of all-cause and cardiovascular mortality, persisting after rigorous adjustment for demographic, metabolic, and clinical confounders. In contrast, the apparent protective association of hepatitis B surface antibody (HBVAb) positivity observed in unadjusted analyses was fully attenuated when accounting for age, comorbidities, and metabolic dysfunction, suggesting no direct causal link between HBVAb status and mortality. Critically, the joint effect analysis revealed that individuals with high SII and HBVAb-negative serostatus faced the highest mortality risk, underscoring the clinical value of integrating inflammatory and immune markers for risk stratification. Our findings advance the understanding of systemic inflammation as a central driver of mortality, transcending traditional risk factors. The robustness of SII across adjustment models supports its utility as a low-cost prognostic biomarker, particularly in resource-limited settings. Conversely, the nullification of HBVAb's association highlights the need to contextualize HBV immunity within populations' baseline inflammatory and comorbidity burdens.**

Comment 2: Need to explain more clearly the criteria for classifying high/low SII, and how to stratify based on clinical risk groups.

Reply 2: The classification of SII into high and low groups was performed based on the status of HBsAb. First, participants were divided into two groups based on their HBsAb status: positive and negative. In the HBsAb-positive group, the Systemic Immune-Inflammation Index (SII) was categorized into high and low groups using the R package surv_cutpoint. Similarly, in the HBsAb-negative group, SII was also categorized into high and low groups using the same R package. This approach resulted in four distinct groups based on both HBsAb status and SII levels.

Changes in the text: Based on Comment 2, we have added a clarification on the grouping criteria in the relevant section of the Results: **The classification of SII into high and low groups was performed based on the status of HBsAb. First, participants were divided into two groups based on their HBsAb status: positive and negative. In the HBsAb-positive group, the Systemic Immune-Inflammation Index (SII) was categorized into high and low groups using the R package surv_cutpoint. Similarly, in the HBsAb-negative group, SII was also categorized into high and low groups using the same R package.**

Comment 3: Authors may consider providing more details about the specific NHANES datasets and variables used to enhance the reproducibility of the study.

Reply 3: We have addressed this comment by providing more details about the specific NHANES datasets and variables used in our study. These details are now clearly presented in Table 1, which includes relevant information for enhancing the reproducibility of the study

Comment 4: Write concisely, avoid redundancy. Some sentences are lengthy and repeat ideas from the Results section. The author should focus on the key findings of the research instead of explaining each numerical detail.

Reply 4: We appreciate the reviewer's valuable feedback. We acknowledge the concern regarding redundancy and overly lengthy sentences. In response, we have revised the manuscript to be more concise, focusing on the key findings of the research. We have removed repetitive explanations and ensured that the Results section remains succinct while still conveying the necessary information. We hope that these revisions improve the clarity and readability of the manuscript. Thank you for your helpful suggestion.

Changes in the text: Based on Comment 4, we revised the results paragraphs:

HBV Surface Antibody and All-Cause Mortality:

HBV antibody positivity showed a lower risk of all-cause mortality in the crude model (HR = 0.491, p < 0.0001). After adjusting for age and sex (Model 1), the HR increased and became non-significant (HR = 0.909, p = 0.072). Further adjustments in Models 2 and 3 also showed no significant association (HR = 0.878, p = 0.018 and HR = 0.906, p = 0.086, respectively), indicating that the

protective effect of HBV antibody positivity diminishes after controlling for other factors. Details are provided in Table 3A.

HBV Surface Antibody and Cardiovascular Mortality:

HBV antibody positivity showed a protective effect against cardiovascular mortality in the crude model (HR = 0.478, $p < 0.0001$). However, after adjustments for age and sex (Model 1), the HR became non-significant (HR = 0.981, p = 0.829). Models 2 and 3 also showed no significant association (HR = 0.920, p = 0.347 and HR = 0.946, p = 0.551, respectively). Details are provided in Table 3B. All-cause mortality, CVD death, and non-CVD death for HBV antibody-negative and -positive groups are shown in Figures 2a, 2b, and 2c.

Combined Effect of SII and HBV Surface Antibody on All-Cause Mortality:

The combination of high SII and HBV antibody-negative status showed a strong association with increased all-cause mortality. In the crude model, the HR was 1.575 ($p < 0.0001$), and after adjustments for age and sex, it remained significant (HR = 1.590, $p < 0.0001$). Models 2 and 3 confirmed the continued association between high SII and higher mortality risk (HR = 1.655, $p < 0.0001$ in Model 2 and HR = 1.402, $p < 0.0001$ in Model 3). Details are provided in Table 4A.

Combined Effect of SII and HBV Surface Antibody on Cardiovascular Mortality:

The combination of high SII and HBV antibody-negative status showed the highest cardiovascular mortality risk. In the crude model, the HR was 1.575 ($p < 0.0001$), and after age and sex adjustments, it remained significant (HR = 1.590, $p < 0.0001$). Models 2 and 3 continued to show a strong association between high SII and poor cardiovascular outcomes, with higher HR values in the highest SII group. Details are provided in Table 4B. The all-cause mortality, CVD death, and non-CVD death for the combined SII and HBV antibody status groups are shown in Figures 3a, 3b, and 3c.

Comment 5: Clearly state the biological mechanisms and clinical significance behind the observed reduction in the protective effect of HbsAb after adjusting for confounders.

Reply 5: We thank the reviewer for emphasizing the importance of clarifying the biological and clinical underpinnings of our findings. As suggested, we have expanded the Discussion section to explicitly address the mechanisms and significance of the attenuated HBsAb-mortality association

Changes in the text: Based on comment 5, we have added the following paragraph to Discussion part: "**The observed attenuation of HBVAb's association with mortality after full adjustment likely reflects the dominant influence of age-related comorbidities (e.g., hypertension, CKD, COPD) and metabolic dysfunction (e.g., elevated BMI, dyslipidemia) on mortality risk. These factors collectively contribute to systemic inflammatory states and physiological frailty, which may overshadow the independent protective effects of HBV immunity. For instance, aging is strongly associated with chronic low-grade inflammation ("inflammaging") and reduced immune resilience, while comorbidities such as CKD and hypertension exacerbate oxidative stress and endothelial dysfunction—pathways that independently drive mortality risk. Furthermore, metabolic disturbances like obesity and dyslipidemia are linked to persistent immune activation and pro-inflammatory cytokine production, which may mask the modest anti-inflammatory benefits conferred by HBV surface antibodies. This aligns with prior studies demonstrating that metabolic syndrome and chronic inflammation attenuate vaccine-induced immune responses in older adults. Our findings suggest that in populations with high baseline inflammatory burden or multimorbidity, the protective role of HBVAb may be diluted by competing risk factors. Clinically, this underscores the importance of contextualizing HBV immune status within a patient's broader metabolic and comorbidity profile when assessing mortality risk. Future studies should explore whether targeted interventions to reduce systemic inflammation or manage comorbidities could unmask or amplify the protective effects of HBV immunity in specific subpopulations.**

Comment 6: Compare findings with previous studies to clarify how the results on SII and HbsAb in this manuscript complement or differ from existing literature?

Reply 6: We appreciate the reviewer's insightful comment. Our study addresses a novel research question, as no previous literature has specifically examined the combined impact of SII and HBsAb status on mortality risk. However, our findings on the prognostic value of SII align with and extend emerging evidence from HBV-related liver disease research. For instance, a study by Xue et al. demonstrated that elevated SII (>1024) independently predicts cirrhosis progression in CHB

patients with comorbid NAFLD (HR=1.885, p=0.010), highlighting the role of systemic inflammation in hepatic fibrogenesis. Similarly, a 2024 study reported that SII effectively distinguishes immune-active from immune-tolerant phases in untreated CHB patients, outperforming qHBsAg in HBeAg-positive subgroups. While our study focuses on mortality rather than disease phase classification, these findings reinforce SII's sensitivity to immune-inflammatory dynamics in HBV infection. In advanced HBV-HCC cohorts, Yao et al. (2024) identified SII ≤539.47 as an independent protective factor for progression-free survival during immunotherapy, with reductions in SII correlating with improved survival outcomes. Although our study does not involve cancer patients, this supports the broader clinical relevance of SII in HBV-associated diseases. Notably, our work diverges by evaluating SII in conjunction with HBsAb status — a novel approach that identified high SII/HBsAb-negative individuals as the highest-risk subgroup, a finding not reported in prior literature. These insights position SII as a transdiagnostic biomarker, bridging hepatic and systemic inflammatory risk assessment in HBV management.

Changes in the text: Based on comment 6, we have added the following paragraph to Discussion part:

While our study pioneers the exploration of SII and HBsAb as joint predictors of mortality in a general population, our findings align with emerging evidence on SII's prognostic role in HBV-related pathologies. For instance, a recent study by Guo et al. (2023) in Frontiers in Oncology demonstrated that elevated SII predicts early recurrence in HBV-related hepatocellular carcinoma (HCC) patients after surgery, underscoring its utility in oncology settings. Similarly, our results confirm SII's broader relevance as a marker of systemic inflammation and mortality risk, extending its prognostic value beyond cancer to all-cause and cardiovascular mortality in the general population. Our findings on the prognostic value of SII align with and extend emerging evidence from HBV-related liver disease research. A recent study by Xue et al. demonstrated that elevated SII (cutoff >1024) independently predicts cirrhosis progression in CHB patients with comorbid NAFLD (HR=1.885, p=0.010), emphasizing its role in hepatic fibrogenesis through systemic inflammation(26). This complements our observation that SII remains a robust mortality predictor even after adjusting for metabolic comorbidities, suggesting its broad utility across both liver-specific and systemic outcomes. Furthermore, a 2024 study reported that SII effectively distinguishes immune-active (EPCH/ENCH) from immune-tolerant (EPCI/ENCI) phases in untreated CHB patients, with higher accuracy than qHBsAg in HBeAg-positive subgroups(27). While our study focuses on mortality rather than disease phase classification, these results collectively highlight SII's sensitivity to immune-inflammatory dynamics in HBV infection. Notably, our work diverges by evaluating SII in conjunction with HBsAb status—a novel approach that revealed high SII/HBsAb-negative individuals as the highest-risk subgroup, a finding unreported in prior literature. Importantly, in advanced HBV-HCC cohorts, Yao et al. (2024) identified SII ≤539.47 as an independent protective factor for progression-free survival

(HR=0.54, p=0.03) during immunotherapy, with longitudinal SII reductions correlating with improved survival(28). Though our population lacks cancer diagnoses, this reinforces the clinical relevance of SII monitoring across HBV disease spectra. Together, these studies position SII as a transdiagnostic biomarker, bridging hepatic and systemic inflammatory risk assessment in HBV management.

Reviewer #4

Comment 1: I suggest that the authors review the existing literature on the SII in hepatitis B-related outcomes. Several published papers have assessed the prediction of SII in patients with hepatitis B (e.g., DOI: 10.1007/s12094-024-03596-0, DOI: 10.1097/MEG.0000000000002737, DOI: https://doi.org/10.3855/jidc.19636), and emphasizing the novel contributions of your study.

Reply 1: We thank the reviewer for this valuable suggestion. We have carefully reviewed the recommended literature and incorporated relevant insights into the revised Discussion section.

Changes in the text: Based on Comment 1, we have added this paragraph to Discussion section:

Our findings on the prognostic value of SII align with and extend emerging evidence from HBV-related liver disease research. A recent study by Xue et al. demonstrated that elevated SII (cutoff >1024) independently predicts cirrhosis progression in CHB patients with comorbid NAFLD (HR=1.885, p=0.010), emphasizing its role in hepatic fibrogenesis through systemic inflammation. This complements our observation that SII remains a robust mortality predictor even after adjusting for metabolic comorbidities, suggesting its broad utility across both liver-specific and systemic outcomes. Furthermore, a 2024 study reported

that SII effectively distinguishes immune-active (EPCH/ENCH) from immune-tolerant (EPCI/ENCI) phases in untreated CHB patients, with higher accuracy than qHBsAg in HBeAg-positive subgroups. While our study focuses on mortality rather than disease phase classification, these results collectively highlight SII's sensitivity to immune-inflammatory dynamics in HBV infection. Notably, our work diverges by evaluating SII in conjunction with HBsAb status—a novel approach that revealed high SII/HBsAb-negative individuals as the highest-risk subgroup, a finding unreported in prior literature. Importantly, in advanced HBV-HCC cohorts, Yao et al. (2024) identified SII ≤539.47 as an independent protective factor for progression-free survival (HR=0.54, p=0.03) during immunotherapy, with longitudinal SII reductions correlating with improved survival. Though our population lacks cancer diagnoses, this reinforces the clinical relevance of SII monitoring across HBV disease spectra. Together, these studies position SII as a transdiagnostic biomarker, bridging hepatic and systemic inflammatory risk assessment in HBV management.

Comment 2: To better align with the authors' statement, I suggest replacing reference number 8 with another source, such as this one (DOI: 10.4103/tjem.tjem_198_23)

Reply 2: We appreciate the reviewer's suggestion. We have replaced reference number 8 with the recommended source to better align with the statement.

Changes in the text: Based on Comment 2, we have replaced reference number 8 with the recommended source (DOI: 10.4103/tjem.tjem_198_23).

Comment 3: I suggest that the authors clarify how they categorized the SII quartile from Q1 to Q4 in the method section to improve readers' comprehension.

Reply 3: We thank the reviewer for highlighting the need for methodological clarity. In our study, SII was categorized into quartiles (Q1 – Q4) based on its distribution within the NHANES population. Quartile thresholds were defined as Q1 (<25th percentile), Q2 (25th – 50th percentile), Q3 (50th – 75th percentile), and Q4 (≥75th percentile), and participants were assigned to the corresponding group accordingly.

Changes in the text: Based on Comment 3, we have revised the Methods section**: SII was categorized into quartiles (Q1 – Q4) based on its distribution in the NHANES population: Q1 (<25th percentile), Q2 (25th – <50th percentile), Q3 (50th – <75th percentile), and Q4 (≥75th percentile)**

Comment 4: I think it would be more comprehensive to provide proposed explanations based on existing literature or the authors' insights into why the protective effect of HBV surface antibody positivity decreased after adjusting for other factors in their study.

Reply 4: Thank you for the valuable suggestion. We have expanded the discussion to provide a more detailed explanation for the attenuation of HBV surface antibody's protective effect after full adjustment. The decrease in protective effect likely reflects the dominant influence of age-related comorbidities (e.g., hypertension, CKD) and metabolic dysfunction (e.g., dyslipidemia, obesity) on mortality risk. Chronic inflammation and immune senescence associated with these conditions may mask the modest protective effect of HBV surface antibodies. For instance, elevated pro-inflammatory cytokines (e.g., IL-6, TNF-α) in metabolically compromised populations may impair immune regulation, reducing the protective impact of HBV immunity. This aligns with evidence that metabolic syndrome accelerates immunosenescence, diminishing the efficacy of vaccine-induced immunity in older adults. We have incorporated this discussion in the revised manuscript to enhance clarity and depth.

Changes in the text: Based on Comment 4, we have added the following paragraph to Discussion part: "**The observed attenuation of HBVAb's association with mortality after full adjustment likely reflects the dominant influence of age-related comorbidities (e.g., hypertension, CKD, COPD) and metabolic dysfunction (e.g., elevated BMI, dyslipidemia) on mortality risk. These factors collectively contribute to systemic inflammatory states and physiological frailty, which may overshadow the independent protective effects of HBV immunity. For instance, aging is strongly associated with chronic low-grade inflammation ("inflammaging") and reduced immune resilience, while comorbidities such as CKD and hypertension exacerbate oxidative stress and endothelial dysfunction—pathways that independently drive mortality risk. Furthermore, metabolic disturbances like obesity and dyslipidemia are linked to persistent immune activation and pro-inflammatory cytokine production, which may mask the modest anti-inflammatory benefits conferred by HBV surface antibodies. This aligns with prior studies demonstrating that metabolic syndrome and chronic inflammation attenuate vaccine-induced immune responses in older adults. Our findings suggest that in populations with high baseline inflammatory burden or multimorbidity, the protective role of HBVAb may be diluted by competing risk factors. Clinically, this underscores the importance of contextualizing HBV immune status within a patient's broader metabolic and comorbidity profile when assessing mortality risk. Future studies should explore whether targeted interventions to reduce systemic inflammation or manage comorbidities could unmask or amplify the protective effects of HBV immunity in specific subpopulations.**

Comment 5: References numbered 18, 23, and 24 are not relevant to the authors' discussion in the text. The authors should review these references to ensure they are cited appropriately in relation to their discussion.

Reply 5:We appreciate the reviewer’ s attention to detail. We have carefully reviewed references 18, 23, and 24 and agree that they were not appropriately aligned with the discussion. These references have been either revised or removed to ensure consistency and relevance in the revised manuscript.

Comment 6: I do not see any result tables (Table 1 to Table 4) in the submitted manuscript; therefore, I could not review and comment thoroughly on these results.

Reply 6: We will ensure that all the tables listed in the manuscript are properly included and visible in the revised submission. Data related to standard deviation could be found in the table.

Changes in the text: **We have included all the tables in the manuscript. Please refer to the end of the revised manuscript for details.**

Reviewer #5

Comment 1: The authors explored the combined impact of Hepatitis B antibody status and Systemic Immune-Inflammation Index on Mortality Risk. The author categorised the participants into groups based on the presence or absence of HBs antibodies and analysed the resulting data. As the author states, the HBs antibody-positive group includes both those who have previously been infected with hepatitis B and those who have received vaccination. The inability to distinguish between these two groups is a limitation of the study. This is a limitation that should be stated explicitly.

Reply 1: We appreciate the reviewer’ s insightful comment. We agree that the inability to distinguish between individuals with prior HBV infection and those with vaccine-induced immunity is a notable limitation. This distinction could have important implications for interpreting the prognostic value of HBsAb positivity. We have explicitly acknowledged this limitation in the revised manuscript and discussed its potential impact on the study’ s findings.

Changes in the text: Based on Comment 1, we have explicitly stated in the limitations section that the inability to distinguish between past HBV infection and vaccine-induced immunity is a limitation of the study and discussed its potential impact on the findings: **The inability to distinguish between past HBV infection and vaccine-induced immunity in the HBs antibody-positive group is a limitation that may influence the interpretation of the results.**

We would like to sincerely thank the editor and reviewers for their thoughtful and constructive feedback. Your insights have been invaluable in improving the clarity and quality of the manuscript. We appreciate the time and effort you have dedicated to reviewing our work, and we believe the revisions made have strengthened the manuscript. We are grateful for your support and look forward to your continued guidance throughout the publication process.
