# Supplementary material for: Exploring the combined impact of hepatitis B antibody status and systemic immune-inflammation index on mortality risk: A population-based study
Source: PLoS One. 2025 Jul 21;20(7):e0328400. doi: 10.1371/journal.pone.0328400 (PMC12279158; doi:10.1371/journal.pone.0328400)
Supplement: Attachment — Submitted filename: Response letter to Reviewers .docx [file pone.0328400.s004.docx]

**Response letter to Reviewers**

Dear Editors and Reviewers,

We would like to thank you for reading our paper and sharing very valuable comments, concerning the manuscript we submitted entitled “Exploring the Combined Impact of Hepatitis B Antibody Status and Systemic Immune-Inflammation Index on Mortality Risk: A Population-Based Study”.

We have carefully looked through the points where you have made a comment, and a revision was made based on the original manuscript which we hope to meet with approval. Here, we would like to address point-by-point concerning the details (the replies are highlighted in blue).

**Reviewer #1**

Comment 1: What were the other covariates considered for full adjustment outside those listed in the results?

Reply 1:We sincerely thank the reviewer for this critical question. Our multivariable models were adjusted in a stepwise manner as follows:

Crude Model: Adjusted only for HBV surface antibody status.

Model 1: Adjusted for HBV surface antibody status, age, and sex.

Model 2: Added socioeconomic and demographic covariates:

Race/ethnicity (eth1)

Family income (Family_income)

Education level (edu)

Marital status (marital)

Model 3 (Fully Adjusted): Further included metabolic, behavioral, and clinical covariates:

Anthropometric: Body mass index (BMI)

Lipid Profile: Total cholesterol, LDL, HDL, triglycerides (TG)

Renal Function: CKD-EPI estimated glomerular filtration rate (CKD_EPI_Scr_2009)

Liver Function: Albumin

Behavioral Factors: Smoking status (smoke), alcohol use (alcohol.user)

Comorbidities: Chronic kidney disease (CKD), hypertension, chronic obstructive pulmonary disease (COPD)

These covariates were selected based on their established roles as confounders in mortality risk and prior literature on HBV and systemic inflammation. The stepwise adjustment approach ensures transparency in evaluating how additional covariates influence the association between HBV antibody status, SII, and mortality outcomes.

Changes in the text:

**Added to the Statistical Analysis subsection in Methods:**

**"Cox proportional hazards models were adjusted in three sequential tiers:**

**Model 1: Age and sex; Model 2: Model 1 + race/ethnicity, family income, education, and marital status; Model 3: Model 2 + BMI, lipid profile (total cholesterol, LDL, HDL, TG), albumin, renal function (CKD-EPI eGFR), smoking, alcohol use, and comorbidities (CKD, hypertension, COPD)."**

Comment 2: Was the nullification of the effect of HBVAb more pronounced with certain "other" covariates, if so which ones and to what extent? Was the effect due to a full combination of all covariates or just a combination of some and if so what combination(s)?

Reply 2: Thank you for raising this important question. In our analysis, the attenuation of HBVAb’s protective effect on mortality outcomes was most notably observed after adjusting for age, comorbidities (hypertension, CKD, COPD), and metabolic factors (BMI, lipid profile). While socioeconomic covariates (e.g., income, education) had minimal impact on the association, the inclusion of clinical and metabolic variables in Model 3 substantially contributed to the nullification of the effect. This attenuation was not attributable to any single covariate but rather emerged from the combined adjustment of age, metabolic status, and comorbidities. For example, age and comorbidities likely reflect the cumulative burden of chronic disease and frailty, which may dominate mortality risk in older populations, thereby overshadowing the modest protective effects of HBVAb. Similarly, metabolic factors such as BMI and lipid levels may confound the relationship by linking systemic inflammation to both HBV immunity and mortality.

Changes in the text: Based on comment 2, we have added the following paragraph to Discussion part: "**The observed attenuation of HBVAb’s association with mortality after full adjustment likely reflects the dominant influence of age-related comorbidities (e.g., hypertension, CKD, COPD) and metabolic dysfunction (e.g., elevated BMI, dyslipidemia) on mortality risk. These factors collectively contribute to systemic inflammatory states and physiological frailty, which may overshadow the independent protective effects of HBV immunity. For instance, aging is strongly associated with chronic low-grade inflammation (“inflammaging”) and reduced immune resilience, while comorbidities such as CKD and hypertension exacerbate oxidative stress and endothelial dysfunction—pathways that independently drive mortality risk. Furthermore, metabolic disturbances like obesity and dyslipidemia are linked to persistent immune activation and pro-inflammatory cytokine production, which may mask the modest anti-inflammatory benefits conferred by HBV surface antibodies. This aligns with prior studies demonstrating that metabolic syndrome and chronic inflammation attenuate vaccine-induced immune responses in older adults. Our findings suggest that in populations with high baseline inflammatory burden or multimorbidity, the protective role of HBVAb may be diluted by competing risk factors. Clinically, this underscores the importance of contextualizing HBV immune status within a patient’s broader metabolic and comorbidity profile when assessing mortality risk. Future studies should explore whether targeted interventions to reduce systemic inflammation or manage comorbidities could unmask or amplify the protective effects of HBV immunity in specific subpopulations.**

."

Comment 3: What is the unit of time measurement on the x-axes of the graphs? months, years, age?

Reply 3: We sincerely thank the reviewer for highlighting this omission. The x-axis of all survival curves (Figures 1a–3c) represents follow-up time in weeks. We have revised the figure captions and corresponding descriptions in the Results section to explicitly clarify the unit of time measurement.

Changes in the text: Based on Comment 3 ，We have added to the Results section :**Survival analyses of all-cause mortality, cardiovascular disease (CVD)-related mortality, and non-CVD-related mortality in the high and low systemic immune-inflammation index (SII) groups are presented in Figure 1a, 1b, and 1c, respectively, with follow-up time measured in weeks.**

Comment 4: The conclusion is misleading, should state that the correlation of HBVAb with mortality was not apparent when fully adjusted for by other factors.

Reply 4: We sincerely thank the reviewer for this crucial correction. We agree that the original conclusion could be misinterpreted and have revised it to explicitly clarify that the association between HBV surface antibody (HBVAb) status and mortality was no longer apparent after full adjustment for covariates.

Changes in the text: Based on Comment 4, we have revised Conclusion section:: **This large-scale study demonstrates that elevated systemic immune-inflammation index (SII) independently predicts increased risks of all-cause and cardiovascular mortality, persisting after rigorous adjustment for demographic, metabolic, and clinical confounders. In contrast, the apparent protective association of hepatitis B surface antibody (HBVAb) positivity observed in unadjusted analyses was fully attenuated when accounting for age, comorbidities, and metabolic dysfunction, suggesting no direct causal link between HBVAb status and mortality. Critically, the joint effect analysis revealed that individuals with high SII and HBVAb-negative serostatus faced the highest mortality risk, underscoring the clinical value of integrating inflammatory and immune markers for risk stratification. Our findings advance the understanding of systemic inflammation as a central driver of mortality, transcending traditional risk factors. The robustness of SII across adjustment models supports its utility as a low-cost prognostic biomarker, particularly in resource-limited settings. Conversely, the nullification of HBVAb’s association highlights the need to contextualize HBV immunity within populations’ baseline inflammatory and comorbidity burdens.**

**Reviewer #2**

Comment 1: What were the authors' cutoff values for high and low PNI, SII, and FIB-4 values? How do these cutoffs compare to those from previous studies?

Reply 1: We sincerely thank the reviewer for catching this oversight. The phrasing has been revised to “2010 to 2020” throughout the manuscript to ensure clarity and consistency.

Changes in the text: Revised in Methods section (Data Source subsection):

**"Data from 2010 to 2020 were utilized to examine the relationship between hepatitis B virus (HBV) antibody status and the systemic immune-inflammation index (SII)."**

Comment 2: Page 11: BMI should be written in full.

Reply 2: We thank the reviewer for pointing this out. We have expanded BMI to Body Mass Index (BMI) at its first mention in the manuscript to ensure clarity and adherence to academic writing standards.

Changes in the text: Based on Comment 2, we have made the following revisions: **Additionally, participants with complete survival data, as well as detailed information on covariates (such as age, sex, race/ethnicity, Body Mass Index (BMI), smoking history, diabetes, and hypertension), were included to ensure robust analysis.**

Comment 3:Page 11: Why was data on alcohol intake omitted?

Reply 3:We have added the data on alcohol intake in the relevant section. Thank you for highlighting this.

Changes in the text: We have added the data on alcohol intake in the relevant section:

**Statistical Analysis**

**Descriptive statistics were calculated for all included variables. Continuous variables were expressed as means ± standard deviations, while categorical variables were presented as frequencies and percentages. Differences in clinical characteristics between HBV antibody-positive and negative groups were evaluated using independent t-tests for continuous variables and chi-square tests for categorical variables. To assess the relationship between SII and HBV antibody status, logistic regression analysis was employed. Three models were constructed: the unadjusted model (without covariate adjustment), the minimally adjusted model (adjusting for age, sex, race/ethnicity, BMI, smoking, diabetes, and hypertension), and the fully adjusted model (adjusting for all covariates, including dietary factors and other health behaviors). Cox proportional hazards models were adjusted in three sequential tiers:**

**Model 1: Age and sex; Model 2: Model 1 + race/ethnicity, family income, education, and marital status; Model 3: Model 2 + BMI, lipid profile (total cholesterol, LDL, HDL, TG), albumin, renal function (CKD-EPI eGFR), smoking, alcohol use, and comorbidities (CKD, hypertension, COPD).The association between SII and clinical outcomes, including liver function, was further evaluated using survival analysis techniques, such as Kaplan-Meier curves and Cox proportional hazards models.**

Comment 4:Statistical analysis:

Was a bivariate analysis done? If yes, please include the results.

The dietary factors and health behaviors assessed should be clearly stated.

Reply 4:Thank you for highlighting these points. We conducted a bivariate analysis, and the results have been included. Our analysis models were structured as follows: crude model: HBV_surface_antibody; model 1: HBV_surface_antibody, age, sex; model 2: HBV_surface_antibody, age, sex, eth1, family_income, edu, marital; model 3: HBV_surface_antibody, age, sex, eth1, marital, family_income, edu, BMI, total_cholesterol, LDL, HDL, TG, albumin, CKD_EPI_Scr_2009, smoke, alcohol_user, CKD, hypertension, COPD. The relevant results are presented in the table; you may have missed them as they were not clearly displayed. We will ensure this is addressed in the revised manuscript.

Changes in the text: We have presented the relevant results in the table here. We will ensure that the table is visible in the revised version:

Comment 5:Page 12: The respective proportions of the four groups of patients should be included.

Reply 5:We thank the reviewer for highlighting this important point. The proportions of the four patient groups (high/low SII with HBsAb-negative/positive) are indeed presented in Table 1 of the manuscript. To ensure clarity, we have explicitly stated these proportions in the Results section and confirmed that all tables are included in the revised submission.

Changes in the text: We have presented the relevant results in the table here. We will ensure that the table is visible in the revised version:

Comment 6:Page 12: The spelling of "included" should be corrected

Reply 6:We thank the reviewer for pointing out this mistake. We have corrected the spelling of "included" on page 12

Changes in the text:

This study included 43,539 patients, categorized into four groups based on systemic immune-inflammation index (SII) and hepatitis B surface antibody (HBsAb) status: high SII and HBsAb-negative (17,677), high SII and HBsAb-positive (4,090), low SII and HBsAb-negative (16,656), and low SII and HBsAb-positive (5,116). The mean age was 46.99 years, with older patients in the high SII groups (high SII/HBsAb-negative: 49.33 years) and younger patients in the low SII/HBsAb-positive group (39.80 years). Mean BMI was 28.80 kg/m², highest in the high SII/HBsAb-negative group (29.55 kg/m²) and lowest in the low SII/HBsAb-positive group (27.09 kg/m²). Lipid profiles showed higher triglycerides and total cholesterol in high SII groups, while HDL levels were higher in the low SII/HBsAb-positive group (1.42 mmol/L). Renal function, assessed by CKD-EPI eGFR, was lower in high SII groups (92.17 mL/min/1.73 m²) and higher in HBsAb-positive groups (100.73 mL/min/1.73 m²). Females predominated in the high SII/HBsAb-positive group (62.08%), while males were more common in the low SII/HBsAb-negative group (54.13%). Non-Hispanic Whites comprised the majority (69.81%), with higher diversity in the low SII/HBsAb-positive group. Education levels were higher in the high SII/HBsAb-positive group (69.63% above high school). Hypertension and CKD prevalence were higher in high SII groups, while COPD prevalence was lowest in the low SII/HBsAb-positive group (1.75%). In summary, high SII groups exhibited higher cardiometabolic risk profiles, whereas HBsAb-positive groups were younger with more favorable metabolic indicators. **Details of included patients are shown in Table1.**

Comment 7:No table was sighted. Could you kindly make available all the tables listed in the body of the manuscript so that the review process can be completed. Also make available the standard deviation value for age and BMI

Reply 7: We will ensure that all the tables listed in the manuscript are properly included and visible in the revised submission. Data related to standard deviation could be found in the table.

Changes in the text: **We have included all the tables in the manuscript. Please refer to the end of the revised manuscript for details.**

**Reviewer #3**

Comment 1: Some conclusions weaken after adjustment and the manuscript does not provide sufficient explanation for the biological mechanisms underlying these changes. The author should clarify the reproducibility of the study and consider providing additional explanations or sensitivity analysis to strengthen the interpretation of results

Reply 1: We have addressed this comment by expanding the discussion to explain the attenuation of HBVAb’s protective effect after full adjustment. Specifically, the weakening of HBVAb’s association with mortality likely reflects the dominant influence of age-related comorbidities (e.g., hypertension, CKD) and metabolic dysfunction (e.g., dyslipidemia, obesity) in driving mortality risk. These factors are closely linked to systemic inflammation ("inflammaging") and immune senescence, which may obscure the modest protective effects of HBV immunity. Chronic inflammation in populations with high comorbidity burdens can disrupt immune regulation, reducing the potential benefits of HBVAb. We have incorporated the following expanded discussion in the revised manuscript:

“The attenuation of HBVAb’s association with mortality after full adjustment may be attributed to the interplay between chronic inflammation and immune exhaustion in populations with high comorbidity burdens. Aging and metabolic disorders promote a pro-inflammatory milieu (e.g., elevated IL-6, TNF-α) that overwhelms the anti-inflammatory effects of HBV surface antibodies. This aligns with evidence that metabolic syndrome accelerates immunosenescence, reducing the efficacy of vaccine-induced immunity in older adults. Thus, HBVAb’s protective role may be context-dependent, limited to populations with preserved metabolic health and lower inflammatory activity.”

We have also refined the conclusion to align with these findings:

“This large-scale study demonstrates that elevated systemic immune-inflammation index (SII) independently predicts increased risks of all-cause and cardiovascular mortality, persisting after rigorous adjustment for demographic, metabolic, and clinical confounders. In contrast, the apparent protective association of hepatitis B surface antibody (HBVAb) positivity observed in unadjusted analyses was fully attenuated when accounting for age, comorbidities, and metabolic dysfunction, suggesting no direct causal link between HBVAb status and mortality. Critically, the joint effect analysis revealed that individuals with high SII and HBVAb-negative serostatus faced the highest mortality risk, underscoring the clinical value of integrating inflammatory and immune markers for risk stratification. Our findings advance the understanding of systemic inflammation as a central driver of mortality, transcending traditional risk factors. The robustness of SII across adjustment models supports its utility as a low-cost prognostic biomarker, particularly in resource-limited settings. Conversely, the nullification of HBVAb’s association highlights the need to contextualize HBV immunity within populations’ baseline inflammatory and comorbidity burdens.”

Changes in the text: Based on comment1, we added this paragraph in discussion section: **The observed attenuation of HBVAb’s association with mortality after full adjustment likely reflects the dominant influence of age-related comorbidities (e.g., hypertension, CKD, COPD) and metabolic dysfunction (e.g., elevated BMI, dyslipidemia) on mortality risk. These factors collectively contribute to systemic inflammatory states and physiological frailty, which may overshadow the independent protective effects of HBV immunity. For instance, aging is strongly associated with chronic low-grade inflammation (“inflammaging”) and reduced immune resilience, while comorbidities such as CKD and hypertension exacerbate oxidative stress and endothelial dysfunction—pathways that independently drive mortality risk. Furthermore, metabolic disturbances like obesity and dyslipidemia are linked to persistent immune activation and pro-inflammatory cytokine production, which may mask the modest anti-inflammatory benefits conferred by HBV surface antibodies. This aligns with prior studies demonstrating that metabolic syndrome and chronic inflammation attenuate vaccine-induced immune responses in older adults. Our findings suggest that in populations with high baseline inflammatory burden or multimorbidity, the protective role of HBVAb may be diluted by competing risk factors. Clinically, this underscores the importance of contextualizing HBV immune status within a patient’s broader metabolic and comorbidity profile when assessing mortality risk. Future studies should explore whether targeted interventions to reduce systemic inflammation or manage comorbidities could unmask or amplify the protective effects of HBV immunity in specific subpopulations.**

We revised the Conlusion section as follows: **This large-scale study demonstrates that elevated systemic immune-inflammation index (SII) independently predicts increased risks of all-cause and cardiovascular mortality, persisting after rigorous adjustment for demographic, metabolic, and clinical confounders. In contrast, the apparent protective association of hepatitis B surface antibody (HBVAb) positivity observed in unadjusted analyses was fully attenuated when accounting for age, comorbidities, and metabolic dysfunction, suggesting no direct causal link between HBVAb status and mortality. Critically, the joint effect analysis revealed that individuals with high SII and HBVAb-negative serostatus faced the highest mortality risk, underscoring the clinical value of integrating inflammatory and immune markers for risk stratification. Our findings advance the understanding of systemic inflammation as a central driver of mortality, transcending traditional risk factors. The robustness of SII across adjustment models supports its utility as a low-cost prognostic biomarker, particularly in resource-limited settings. Conversely, the nullification of HBVAb’s association highlights the need to contextualize HBV immunity within populations’ baseline inflammatory and comorbidity burdens.**

Comment 2: Need to explain more clearly the criteria for classifying high/low SII, and how to stratify based on clinical risk groups.

Reply 2:  The classification of SII into high and low groups was performed based on the status of HBsAb. First, participants were divided into two groups based on their HBsAb status: positive and negative. In the HBsAb-positive group, the Systemic Immune-Inflammation Index (SII) was categorized into high and low groups using the R package surv_cutpoint. Similarly, in the HBsAb-negative group, SII was also categorized into high and low groups using the same R package. This approach resulted in four distinct groups based on both HBsAb status and SII levels.

Changes in the text: Based on Comment 2, we have added a clarification on the grouping criteria in the relevant section of the Results: **The classification of SII into high and low groups was performed based on the status of HBsAb. First, participants were divided into two groups based on their HBsAb status: positive and negative. In the HBsAb-positive group, the Systemic Immune-Inflammation Index (SII) was categorized into high and low groups using the R package surv_cutpoint. Similarly, in the HBsAb-negative group, SII was also categorized into high and low groups using the same R package.**

Comment 3: Authors may consider providing more details about the specific NHANES datasets and variables used to enhance the reproducibility of the study.

Reply 3:  We have addressed this comment by providing more details about the specific NHANES datasets and variables used in our study. These details are now clearly presented in Table 1, which includes relevant information for enhancing the reproducibility of the study

Comment 4: Write concisely, avoid redundancy. Some sentences are lengthy and repeat ideas from the Results section. The author should focus on the key findings of the research instead of explaining each numerical detail.

Reply 4:  We appreciate the reviewer’s valuable feedback. We acknowledge the concern regarding redundancy and overly lengthy sentences. In response, we have revised the manuscript to be more concise, focusing on the key findings of the research. We have removed repetitive explanations and ensured that the Results section remains succinct while still conveying the necessary information. We hope that these revisions improve the clarity and readability of the manuscript. Thank you for your helpful suggestion.

Changes in the text: Based on Comment 4, we revised the results paragraphs:

**HBV Surface Antibody and All-Cause Mortality:**

**HBV antibody positivity showed a lower risk of all-cause mortality in the crude model (HR = 0.491, p < 0.0001). After adjusting for age and sex (Model 1), the HR increased and became non-significant (HR = 0.909, p = 0.072). Further adjustments in Models 2 and 3 also showed no significant association (HR = 0.878, p = 0.018 and HR = 0.906, p = 0.086, respectively), indicating that the protective effect of HBV antibody positivity diminishes after controlling for other factors. Details are provided in Table 3A.**

**HBV Surface Antibody and Cardiovascular Mortality:**

**HBV antibody positivity showed a protective effect against cardiovascular mortality in the crude model (HR = 0.478, p < 0.0001). However, after adjustments for age and sex (Model 1), the HR became non-significant (HR = 0.981, p = 0.829). Models 2 and 3 also showed no significant association (HR = 0.920, p = 0.347 and HR = 0.946, p = 0.551, respectively). Details are provided in Table 3B. All-cause mortality, CVD death, and non-CVD death for HBV antibody-negative and -positive groups are shown in Figures 2a, 2b, and 2c.**

**Combined Effect of SII and HBV Surface Antibody on All-Cause Mortality:**

**The combination of high SII and HBV antibody-negative status showed a strong association with increased all-cause mortality. In the crude model, the HR was 1.575 (p < 0.0001), and after adjustments for age and sex, it remained significant (HR = 1.590, p < 0.0001). Models 2 and 3 confirmed the continued association between high SII and higher mortality risk (HR = 1.655, p < 0.0001 in Model 2 and HR = 1.402, p < 0.0001 in Model 3). Details are provided in Table 4A.**

**Combined Effect of SII and HBV Surface Antibody on Cardiovascular Mortality:**

**The combination of high SII and HBV antibody-negative status showed the highest cardiovascular mortality risk. In the crude model, the HR was 1.575 (p < 0.0001), and after age and sex adjustments, it remained significant (HR = 1.590, p < 0.0001). Models 2 and 3 continued to show a strong association between high SII and poor cardiovascular outcomes, with higher HR values in the highest SII group. Details are provided in Table 4B. The all-cause mortality, CVD death, and non-CVD death for the combined SII and HBV antibody status groups are shown in Figures 3a, 3b, and 3c.**

Comment 5: Clearly state the biological mechanisms and clinical significance behind the observed reduction in the protective effect of HbsAb after adjusting for confounders.

Reply 5: We thank the reviewer for emphasizing the importance of clarifying the biological and clinical underpinnings of our findings. As suggested, we have expanded the Discussion section to explicitly address the mechanisms and significance of the attenuated HBsAb-mortality association

Changes in the text: Based on comment 5, we have added the following paragraph to Discussion part: "**The observed attenuation of HBVAb’s association with mortality after full adjustment likely reflects the dominant influence of age-related comorbidities (e.g., hypertension, CKD, COPD) and metabolic dysfunction (e.g., elevated BMI, dyslipidemia) on mortality risk. These factors collectively contribute to systemic inflammatory states and physiological frailty, which may overshadow the independent protective effects of HBV immunity. For instance, aging is strongly associated with chronic low-grade inflammation (“inflammaging”) and reduced immune resilience, while comorbidities such as CKD and hypertension exacerbate oxidative stress and endothelial dysfunction—pathways that independently drive mortality risk. Furthermore, metabolic disturbances like obesity and dyslipidemia are linked to persistent immune activation and pro-inflammatory cytokine production, which may mask the modest anti-inflammatory benefits conferred by HBV surface antibodies. This aligns with prior studies demonstrating that metabolic syndrome and chronic inflammation attenuate vaccine-induced immune responses in older adults. Our findings suggest that in populations with high baseline inflammatory burden or multimorbidity, the protective role of HBVAb may be diluted by competing risk factors. Clinically, this underscores the importance of contextualizing HBV immune status within a patient’s broader metabolic and comorbidity profile when assessing mortality risk. Future studies should explore whether targeted interventions to reduce systemic inflammation or manage comorbidities could unmask or amplify the protective effects of HBV immunity in specific subpopulations.**

Comment 6:  Compare findings with previous studies to clarify how the results on SII and HbsAb in this manuscript complement or differ from existing literature?

Reply 6: We appreciate the reviewer’s insightful comment. Our study addresses a novel research question, as no previous literature has specifically examined the combined impact of SII and HBsAb status on mortality risk. However, our findings on the prognostic value of SII align with and extend emerging evidence from HBV-related liver disease research. For instance, a study by Xue et al. demonstrated that elevated SII (>1024) independently predicts cirrhosis progression in CHB patients with comorbid NAFLD (HR=1.885, p=0.010), highlighting the role of systemic inflammation in hepatic fibrogenesis. Similarly, a 2024 study reported that SII effectively distinguishes immune-active from immune-tolerant phases in untreated CHB patients, outperforming qHBsAg in HBeAg-positive subgroups. While our study focuses on mortality rather than disease phase classification, these findings reinforce SII’s sensitivity to immune-inflammatory dynamics in HBV infection. In advanced HBV-HCC cohorts, Yao et al. (2024) identified SII ≤539.47 as an independent protective factor for progression-free survival during immunotherapy, with reductions in SII correlating with improved survival outcomes. Although our study does not involve cancer patients, this supports the broader clinical relevance of SII in HBV-associated diseases. Notably, our work diverges by evaluating SII in conjunction with HBsAb status—a novel approach that identified high SII/HBsAb-negative individuals as the highest-risk subgroup, a finding not reported in prior literature. These insights position SII as a transdiagnostic biomarker, bridging hepatic and systemic inflammatory risk assessment in HBV management.

Changes in the text: Based on comment 6, we have added the following paragraph to Discussion part:

**While our study pioneers the exploration of SII and HBsAb as joint predictors of mortality in a general population, our findings align with emerging evidence on SII’s prognostic role in HBV-related pathologies. For instance, a recent study by Guo et al. (2023) in Frontiers in Oncology demonstrated that elevated SII predicts early recurrence in HBV-related hepatocellular carcinoma (HCC) patients after surgery, underscoring its utility in oncology settings. Similarly, our results confirm SII’s broader relevance as a marker of systemic inflammation and mortality risk, extending its prognostic value beyond cancer to all-cause and cardiovascular mortality in the general population. Our findings on the prognostic value of SII align with and extend emerging evidence from HBV-related liver disease research. A recent study by Xue et al. demonstrated that elevated SII (cutoff >1024) independently predicts cirrhosis progression in CHB patients with comorbid NAFLD (HR=1.885, p=0.010), emphasizing its role in hepatic fibrogenesis through systemic inflammation(26). This complements our observation that SII remains a robust mortality predictor even after adjusting for metabolic comorbidities, suggesting its broad utility across both liver-specific and systemic outcomes. Furthermore, a 2024 study reported that SII effectively distinguishes immune-active (EPCH/ENCH) from immune-tolerant (EPCI/ENCI) phases in untreated CHB patients, with higher accuracy than qHBsAg in HBeAg-positive subgroups(27). While our study focuses on mortality rather than disease phase classification, these results collectively highlight SII’s sensitivity to immune-inflammatory dynamics in HBV infection. Notably, our work diverges by evaluating SII in conjunction with HBsAb status—a novel approach that revealed high SII/HBsAb-negative individuals as the highest-risk subgroup, a finding unreported in prior literature. Importantly, in advanced HBV-HCC cohorts, Yao et al. (2024) identified SII ≤539.47 as an independent protective factor for progression-free survival (HR=0.54, p=0.03) during immunotherapy, with longitudinal SII reductions correlating with improved survival(28). Though our population lacks cancer diagnoses, this reinforces the clinical relevance of SII monitoring across HBV disease spectra. Together, these studies position SII as a transdiagnostic biomarker, bridging hepatic and systemic inflammatory risk assessment in HBV management.**

**Reviewer #4**

Comment 1: I suggest that the authors review the existing literature on the SII in hepatitis B-related outcomes. Several published papers have assessed the prediction of SII in patients with hepatitis B (e.g., DOI: 10.1007/s12094-024-03596-0, DOI: 10.1097/MEG.0000000000002737, DOI: https://doi.org/10.3855/jidc.19636), and emphasizing the novel contributions of your study.

Reply 1: We thank the reviewer for this valuable suggestion. We have carefully reviewed the recommended literature and incorporated relevant insights into the revised Discussion section.

Changes in the text: Based on Comment 1, we have added this paragraph to Discussion section:

**Our findings on the prognostic value of SII align with and extend emerging evidence from HBV-related liver disease research. A recent study by Xue et al. demonstrated that elevated SII (cutoff >1024) independently predicts cirrhosis progression in CHB patients with comorbid NAFLD (HR=1.885, p=0.010), emphasizing its role in hepatic fibrogenesis through systemic inflammation. This complements our observation that SII remains a robust mortality predictor even after adjusting for metabolic comorbidities, suggesting its broad utility across both liver-specific and systemic outcomes. Furthermore, a 2024 study reported that SII effectively distinguishes immune-active (EPCH/ENCH) from immune-tolerant (EPCI/ENCI) phases in untreated CHB patients, with higher accuracy than qHBsAg in HBeAg-positive subgroups. While our study focuses on mortality rather than disease phase classification, these results collectively highlight SII’s sensitivity to immune-inflammatory dynamics in HBV infection. Notably, our work diverges by evaluating SII in conjunction with HBsAb status—a novel approach that revealed high SII/HBsAb-negative individuals as the highest-risk subgroup, a finding unreported in prior literature. Importantly, in advanced HBV-HCC cohorts, Yao et al. (2024) identified SII ≤539.47 as an independent protective factor for progression-free survival (HR=0.54, p=0.03) during immunotherapy, with longitudinal SII reductions correlating with improved survival. Though our population lacks cancer diagnoses, this reinforces the clinical relevance of SII monitoring across HBV disease spectra. Together, these studies position SII as a transdiagnostic biomarker, bridging hepatic and systemic inflammatory risk assessment in HBV management.**

Comment 2:  To better align with the authors' statement, I suggest replacing reference number 8 with another source, such as this one (DOI: 10.4103/tjem.tjem_198_23)

Reply 2: We appreciate the reviewer’s suggestion. We have replaced reference number 8 with the recommended source to better align with the statement.

Changes in the text: Based on Comment 2, we have replaced reference number 8 with the recommended source (DOI: 10.4103/tjem.tjem_198_23).

Comment 3:  I suggest that the authors clarify how they categorized the SII quartile from Q1 to Q4 in the method section to improve readers' comprehension.

Reply 3: We thank the reviewer for highlighting the need for methodological clarity. In our study, SII was categorized into quartiles (Q1–Q4) based on its distribution within the NHANES population. Quartile thresholds were defined as Q1 (<25th percentile), Q2 (25th–50th percentile), Q3 (50th–75th percentile), and Q4 (≥75th percentile), and participants were assigned to the corresponding group accordingly.

Changes in the text: Based on Comment 3, we have revised the Methods section**: SII was categorized into quartiles (Q1–Q4) based on its distribution in the NHANES population: Q1 (<25th percentile), Q2 (25th–<50th percentile), Q3 (50th–<75th percentile), and Q4 (≥75th percentile)**

Comment 4: I think it would be more comprehensive to provide proposed explanations based on existing literature or the authors' insights into why the protective effect of HBV surface antibody positivity decreased after adjusting for other factors in their study.

Reply 4: Thank you for the valuable suggestion. We have expanded the discussion to provide a more detailed explanation for the attenuation of HBV surface antibody's protective effect after full adjustment. The decrease in protective effect likely reflects the dominant influence of age-related comorbidities (e.g., hypertension, CKD) and metabolic dysfunction (e.g., dyslipidemia, obesity) on mortality risk. Chronic inflammation and immune senescence associated with these conditions may mask the modest protective effect of HBV surface antibodies. For instance, elevated pro-inflammatory cytokines (e.g., IL-6, TNF-α) in metabolically compromised populations may impair immune regulation, reducing the protective impact of HBV immunity. This aligns with evidence that metabolic syndrome accelerates immunosenescence, diminishing the efficacy of vaccine-induced immunity in older adults. We have incorporated this discussion in the revised manuscript to enhance clarity and depth.

Changes in the text: Based on Comment 4, we have added the following paragraph to Discussion part: "**The observed attenuation of HBVAb’s association with mortality after full adjustment likely reflects the dominant influence of age-related comorbidities (e.g., hypertension, CKD, COPD) and metabolic dysfunction (e.g., elevated BMI, dyslipidemia) on mortality risk. These factors collectively contribute to systemic inflammatory states and physiological frailty, which may overshadow the independent protective effects of HBV immunity. For instance, aging is strongly associated with chronic low-grade inflammation (“inflammaging”) and reduced immune resilience, while comorbidities such as CKD and hypertension exacerbate oxidative stress and endothelial dysfunction—pathways that independently drive mortality risk. Furthermore, metabolic disturbances like obesity and dyslipidemia are linked to persistent immune activation and pro-inflammatory cytokine production, which may mask the modest anti-inflammatory benefits conferred by HBV surface antibodies. This aligns with prior studies demonstrating that metabolic syndrome and chronic inflammation attenuate vaccine-induced immune responses in older adults. Our findings suggest that in populations with high baseline inflammatory burden or multimorbidity, the protective role of HBVAb may be diluted by competing risk factors. Clinically, this underscores the importance of contextualizing HBV immune status within a patient’s broader metabolic and comorbidity profile when assessing mortality risk. Future studies should explore whether targeted interventions to reduce systemic inflammation or manage comorbidities could unmask or amplify the protective effects of HBV immunity in specific subpopulations.**

Comment 5: References numbered 18, 23, and 24 are not relevant to the authors' discussion in the text. The authors should review these references to ensure they are cited appropriately in relation to their discussion.

Reply 5:We appreciate the reviewer’s attention to detail. We have carefully reviewed references 18, 23, and 24 and agree that they were not appropriately aligned with the discussion. These references have been either revised or removed to ensure consistency and relevance in the revised manuscript.

Comment 6: I do not see any result tables (Table 1 to Table 4) in the submitted manuscript; therefore, I could not review and comment thoroughly on these results.

Reply 6: We will ensure that all the tables listed in the manuscript are properly included and visible in the revised submission. Data related to standard deviation could be found in the table.

Changes in the text: **We have included all the tables in the manuscript. Please refer to the end of the revised manuscript for details.**

**Reviewer #5**

Comment 1: The authors explored the combined impact of Hepatitis B antibody status and Systemic Immune-Inflammation Index on Mortality Risk. The author categorised the participants into groups based on the presence or absence of HBs antibodies and analysed the resulting data. As the author states, the HBs antibody-positive group includes both those who have previously been infected with hepatitis B and those who have received vaccination. The inability to distinguish between these two groups is a limitation of the study. This is a limitation that should be stated explicitly.

Reply 1: We appreciate the reviewer’s insightful comment. We agree that the inability to distinguish between individuals with prior HBV infection and those with vaccine-induced immunity is a notable limitation. This distinction could have important implications for interpreting the prognostic value of HBsAb positivity. We have explicitly acknowledged this limitation in the revised manuscript and discussed its potential impact on the study’s findings.

Changes in the text: Based on Comment 1, we have explicitly stated in the limitations section that the inability to distinguish between past HBV infection and vaccine-induced immunity is a limitation of the study and discussed its potential impact on the findings: **The inability to distinguish between past HBV infection and vaccine-induced immunity in the HBs antibody-positive group is a limitation that may influence the interpretation of the results.**

We would like to sincerely thank the editor and reviewers for their thoughtful and constructive feedback. Your insights have been invaluable in improving the clarity and quality of the manuscript. We appreciate the time and effort you have dedicated to reviewing our work, and we believe the revisions made have strengthened the manuscript. We are grateful for your support and look forward to your continued guidance throughout the publication process.
