# Supplementary material for: Exploring the combined impact of hepatitis B antibody status and systemic immune-inflammation index on mortality risk: A population-based study
Source: PLoS One. 2025 Jul 21;20(7):e0328400. doi: 10.1371/journal.pone.0328400 (PMC12279158; doi:10.1371/journal.pone.0328400)
Supplement: Attachment — Submitted filename: Response letter to Reviewer2.docx [file pone.0328400.s005.docx]

**Response letter to Reviewers**

Dear Editors and Reviewers,

We would like to thank you for reading our paper and sharing very valuable comments, concerning the manuscript we submitted entitled “Exploring the Combined Impact of Hepatitis B Antibody Status and Systemic Immune-Inflammation Index on Mortality Risk: A Population-Based Study”.

We have carefully looked through the points where you have made a comment, and a revision was made based on the original manuscript which we hope to meet with approval. Here, we would like to address point-by-point concerning the details (the replies are highlighted in blue).

**Reviewer #2**

Comment 1:  Please clarify that the proposed mechanisms linking elevated SII and the attenuation of HBsAb's protective effect are speculative, and clearly state the need for future mechanistic studies.

Reply 1:Thank you for this insightful comment. We agree that the mechanistic explanations proposed in the discussion remain speculative and should not be interpreted as definitive. While our findings highlight a potential interplay between systemic inflammation and HBV antibody status in shaping mortality risk, the biological underpinnings of these associations require further investigation. In response to your suggestion, we have revised the Discussion to explicitly acknowledge the speculative nature of the proposed mechanisms and to emphasize the need for future mechanistic and longitudinal studies to better elucidate these pathways.

Changes in the text:

**Based on Comment 1, we have added the following paragraph to the Discussion section:**

This finding underscores the importance of considering both immune status and inflammation when evaluating mortality risk(26). While SII provides insight into systemic inflammation, HBV surface antibody status reflects an individual's immune history and protective status against viral infections(27). By combining these two markers, we gain a more nuanced understanding of how immune and inflammatory responses contribute to overall mortality, including cardiovascular outcomes. **It is important to acknowledge that the proposed mechanisms linking elevated SII and the attenuation of the protective effect of HBsAb remain speculative. While our findings suggest a potential interplay between systemic inflammation and immune protection conferred by HBsAb, the underlying biological pathways have yet to be fully elucidated. Future mechanistic studies are warranted to explore how immune status and inflammatory responses interact to influence long-term health outcomes, particularly in HBV-affected populations.**

Comment 2: Discuss more thoroughly how the inability to distinguish vaccine-induced from infection-induced HBsAb may influence the interpretation of findings.

Reply 2: Thank you for this thoughtful comment. We agree that the inability to distinguish between vaccine-induced and infection-induced hepatitis B surface antibody (HBsAb) positivity represents an important limitation in interpreting our findings. These two forms of immunity may reflect different underlying biological contexts—vaccine-induced immunity typically occurs without prior liver inflammation, whereas infection-induced immunity may be associated with residual hepatic injury or systemic inflammation. This heterogeneity could influence both SII levels and mortality risk. In response, we have added a new paragraph to the Discussion section to elaborate on how this distinction may affect the interpretation of our results, particularly regarding the combined effect of HBsAb and SII. We also retained a concise acknowledgment of this limitation in the Limitations section.

Changes in the text:

Based on Comment 2, we have added the following paragraph to the Discussion section: **Moreover, it is important to consider that HBsAb positivity in this study likely includes both vaccine-induced and infection-induced immunity, which may represent biologically distinct immune profiles. Individuals with resolved HBV infection may exhibit different levels of residual inflammation or liver damage compared to those with vaccine-induced immunity, potentially influencing their SII levels and mortality risk. Without differentiation, these underlying differences may confound the interpretation of our findings. Future studies with serological markers such as anti-HBc are needed to disentangle these effects and refine the understanding of how HBV immune history interacts with systemic inflammation.**

We have also added the following sentence to the Limitations section: **Moreover, the NHANES dataset does not distinguish between vaccine- and infection-induced HBsAb positivity, which may obscure differences in inflammatory status and mortality risk related to the underlying source of immunity.**

Comment 3: Provide more detail on how missing data were handled or discuss whether sensitivity analyses were considered.

Reply 3: Thank you for this valuable suggestion. In our analysis, participants with missing key variables—including HBsAb status, SII components (platelet, neutrophil, lymphocyte counts), survival data, or essential covariates—were excluded from the final analytic cohort through listwise deletion. This approach was applied to ensure consistency across models and to minimize bias from partial data. While we did not perform formal sensitivity analyses due to the completeness of the retained dataset and large sample size, we recognize the importance of evaluating robustness in future work. In response to your comment, we have added a clarifying paragraph to the Methods section describing our approach to missing data

Changes in the text: Based on Comment 3, we have added the following paragraph to the Materials and Methods section, under "Study Population": **Participants with missing data on key variables—including HBV antibody testing, SII-related biomarkers (platelet, neutrophil, lymphocyte counts), covariates (age, sex, BMI, comorbidities), or survival outcomes—were excluded using listwise deletion. This method ensured that all models were run on a consistent and complete dataset, thereby improving the comparability and validity of the statistical results.**

Comment 4: Minor grammatical and stylistic revisions are advised to improve the overall clarity and flow of the manuscript.

Reply 4: Thank you for this helpful comment. We have carefully reviewed the entire manuscript and made multiple minor grammatical, stylistic, and wording improvements to enhance clarity, readability, and consistency throughout the text. These edits include refining sentence structure, correcting punctuation, improving transitions between paragraphs, and adjusting word choices for greater precision and academic tone. These revisions do not affect the results or interpretation of the study.

Comment 5: Kindly refer to Table 1, the first variable "Age". The mean age for the four groups are presented. The proportion of participants in each of the 4 groups is also presented. The proportions add up to 1.11 which translates to 111%. Kindly cross-check the proportion and effect correction. The proportions should add up to 1.

Reply 5: Thank you for your careful review. We apologize for the confusion. The numbers in parentheses in Table 1 represent the standard errors (SE) of the means, not proportions. We realize that this was not clearly indicated in the table originally, which may have caused misunderstanding. To clarify this, we have now added a note at the bottom of Table 1 explicitly stating that the values in parentheses represent standard errors.

Changes in the text: Based on Comment 5, we have added the following note to the bottom of Table 1: **Note: Values are presented as mean, with standard error shown in parentheses (e.g., 46.99 (0.19) indicates a mean of 46.99 and a standard error of 0.19).**

We would like to sincerely thank the editor and reviewers for their thoughtful and constructive feedback. Your insights have been invaluable in improving the clarity and quality of the manuscript. We appreciate the time and effort you have dedicated to reviewing our work, and we believe the revisions made have strengthened the manuscript. We are grateful for your support and look forward to your continued guidance throughout the publication process.
